
JOURNAL INFORMATION
==============================
NLM Title Abbreviation: Insights Imaging
Journal ID: Insights Imaging
NLM Journal ID: 101532453

Insights into Imaging

EISSN: 1869-4101
Publisher: Springer

ARTICLE INFORMATION
==============================
PMCID: PMC7231606
PMID: 32419107
DOI: 10.1186/s13244-020-00873-8
Article ID: 873
Article version: 2
Subjects: Meeting Abstracts

ESGAR 2020 Book of Abstracts

Electronic publication date: 2020 May 18
Publication date: 2020 May
Volume: 11
Issue: Suppl 3
Issue sponsor: Publication costs were not supported by sponsorship.
Electronic Location ID: 64
Copyright: © The Author(s) 2020
License: Open AccessThis article is licensed under a Creative Commons Attribution 4.0 International License, which permits use, sharing, adaptation, distribution and reproduction in any medium or format, as long as you give appropriate credit to the original author(s) and the source, provide a link to the Creative Commons licence, and indicate if changes were made. The images or other third party material in this article are included in the article's Creative Commons licence, unless indicated otherwise in a credit line to the material. If material is not included in the article's Creative Commons licence and your intended use is not permitted by statutory regulation or exceeds the permitted use, you will need to obtain permission directly from the copyright holder. To view a copy of this licence, visit http://creativecommons.org/licenses/by/4.0/.
License URL: https://creativecommons.org/licenses/by/4.0/

Conference name: ESGAR Annual Meeting and Postgraduate Course 2020
Conference Acronym: ESGAR 2020
Conference location: Amsterdam, The Netherlands
Conference date: 19-22 May 2020
issue-copyright-statement: © The Author(s) 2020

==============================
European Society ESGAR Gastrointestinal and Abdominal Radiology

### Insights into Imaging

Education and strategies in European radiology

ESGAR 2020 Book of Abstracts / Volume 11 / Supplement 3 / May 2020

ESGAR 2020 / May 19 – 22 / Amsterdam, The Netherlands
31st Annual Meeting and Postgraduate Course

ESR European Society of Radiology

European Society of Gastrointestinal and Abdominal Radiology
ESGAR

### ESGAR

### Annual Meeting and Postgraduate Course

### 2020

### May 19-22

VIRTUAL MEETING

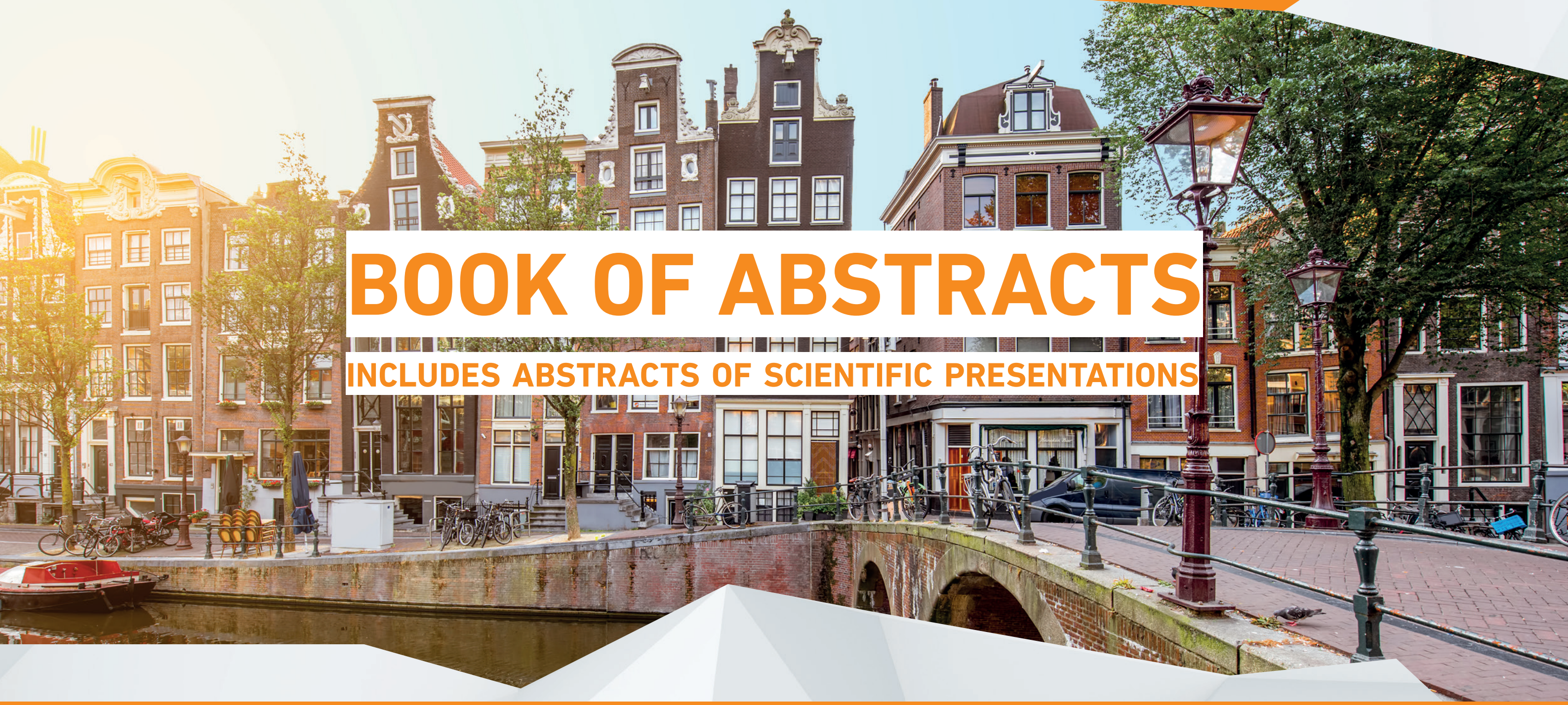

### BOOK OF ABSTRACTS

INCLUDES ABSTRACTS OF SCIENTIFIC PRESENTATIONS

European Society of Gastrointestinal and Abdominal Radiology | www.esgar.org

As we have seen in the recent past, the coronavirus pandemic has a huge impact on our welfare and educational activities. Statements of public health authorities worldwide recommend to not hold workshops or congresses and furthermore, to also not participate in such activities.

Due to an order by the Dutch Government, all gatherings (even of less than 100 people) are prohibited until June 1. The ESGAR 2020 Annual Meeting, with its planned date from May 19 - 22, falls under the scope of this ban. In anticipation of this aggravation of the COVID-19 pandemic and related restrictive measures by the Dutch Government, ESGAR was cancelled on March 20.

Thus, even though the ESGAR 31st Annual Meeting and Postgraduate Course cannot take place in the usual way this year, publishing the ESGAR 2020 Book of Abstracts is a great way to continue to showcase the research, despite the current situation.

European Society
ESGAR
Gastrointestinal and Abdominal Radiology

### TABLE OF CONTENTS

| | |
|---|---|
| Scientific Sessions, Wednesday, May 20 (SS 1 – SS 5) | 8-20 |
| Scientific Sessions, Thursday, May 21 (SS 6 – SS 10) | 21-32 |
| Scientific Sessions, Friday, May 22 (SS 11 – SS 14) | 33-43 |
| Authors' Index | 44-48 |

dates and times refer to the originally planned and cancelled face-to-face meeting in Amsterdam

Insights Imaging (2020) 11 (Suppl 3): 64
https://doi.org/10.1186/s13244-020-00873-8
Published Online: 18 May 2020

### ESGAR EXECUTIVE COMMITTEE

PRESIDENT
*R.G.H. Beets-Tan, Amsterdam/NL*

PRESIDENT-ELECT
*A. Laghi, Rome/IT*

VICE PRESIDENT
*M. Zins, Paris/FR*

SECRETARY/TREASURER
*S.A. Taylor, London/UK*

EDUCATION COMMITTEE CHAIR
*S. Jackson, Plymouth/UK*

MEMBERSHIP COMMITTEE CHAIR
*G. Brancatelli, Palermo/IT*

RESEARCH COMMITTEE CHAIR
*J. Stoker, Amsterdam/NL*

WORKSHOP COMMITTEE CHAIR
*M.A. Bali, Brussels/BE*

MEETING PRESIDENT
*J. Stoker, Amsterdam/NL*

PRE-MEETING PRESIDENT
*L. Curvo-Semedo, Coimbra/PT*

MEMBERS AT LARGE
*A. Ba-Ssalamah, Vienna/AT*
*S. Gourtsoyianni, Athens/GR*
*V. Vilgrain, Clichy/FR*
*C.J. Zech, Basel/CH*

ESGAR EXECUTIVE DIRECTOR
*B. Lindlbauer, Vienna/AT*

### LANGUAGE ABSTRACT EDITOR

*R. Karia, Nottingham/UK*

### ESGAR 2020 MEETING PRESIDENT

*Prof. Dr. Jaap Stoker*
*Professor of Radiology*
*University of Amsterdam*
*Head of Research*
*Vice Chair Department of Radiology and Nuclear Medicine*
*Chair Division Radiology and Nuclear Medicine, Radiotherapy, Biomedical Engineering and Physics, and Pharmacy*
*Amsterdam UMC*
*The Netherlands*

### ESGAR 2020 PROGRAMME COMMITTEE

CHAIR
*M. Zins, Paris/FR*

MEMBERS
*M.A. Bali, Brussels/BE*
*A. Ba-Ssalamah, Vienna/AT*
*R.G.H. Beets-Tan, Amsterdam/NL*
*G. Brancatelli, Palermo/IT*
*L. Curvo-Semedo, Lisbon/PT*
*S. Gourtsoyianni, Athens/GR*
*S. Jackson, Plymouth/UK*
*A. Laghi, Rome/IT*
*J. Stoker, Amsterdam/NL*
*S.A Taylor, London/UK*
*V. Vilgrain, Clichy/FR*
*C.J. Zech, Basel/CH*

### ESGAR 2020 LOCAL ORGANISING COMMITTEE

*R.G.H. Beets-Tan, Amsterdam/NL*
*T. Bollen, Nieuwegein/NL*
*R. de Haas, Groningen/NL*
*K. Horsthuis, Losser/NL*
*L. Oudenhoven, Apeldoorn/NL*
*M. Prokop, Nijmegen/NL*
*J.B.C.M. Puylaert, The Hague/NL*
*S. Robben, Maastricht/NL*
*B. Simons, Rotterdam/NL*

*O. Akhan, Ankara/TR*
*C. Aubé, Angers/FR*
*M.A. Bali, Brussels/BE*
*I. Bargellini, Pisa/IT*
*T. Bartolotta, Palermo/IT*
*A. Ba-Ssalamah, Vienna/AT*
*N. Bastati-Huber, Vienna/AT*
*R.G.H. Beets-Tan, Amsterdam/NL*
*E. Biscaldi, Genoa/IT*
*A. Blachar, Tel Aviv/IL*
*I. Boulay Coletta, Paris/FR*
*G. Brancatelli, Palermo/IT*
*D.J. Breen, Southampton/UK*
*R. Cannella, Palermo/IT*
*F. Caseiro Alves, Coimbra/PT*
*K. Cortis, Msida/MT*
*N. Courcoutsakis, Alexandroupolis/GR*
*L. Crocetti, Pisa/IT*
*L. Curvo-Semedo, Coimbra/PT*
*G. D'Assignies, La Roche-sur-Yon/FR*
*M. Dioguardi Burgio, Paris/FR*
*A. Dohan, Paris/FR*
*R. Dondelinger, Liège/BE*
*M. D'Onofrio, Verona/IT*
*H. Fenlon, Dublin/IE*
*A. Furlan, Pittsburgh, PA/US*
*Y. Gandon, Rennes/FR*
*V. Goh, London/UK*
*M. Gollub, New York, NY/US*
*S. Gourtsoyianni, Athens/GR*
*S. Gryspeerdt, Roeselare/BE*
*L. Guimaraes, North York, ON/CA*
*J.A. Guthrie, Leeds/UK*
*S. Halligan, London/UK*
*A. Hatzidakis, Heraklion/GR*
*T. Helmberger, Munich/DE*
*F. Iafrate, Rome/IT*
*D. Ippolito, Monza/IT*
*S. Jackson, Plymouth/UK*
*M. Karcaaltincaba, Ankara/TR*
*A. Laghi, Rome/IT*
*D. Lambregts, Amsterdam/NL*
*M. Laniado, Dresden/DE*
*P. Lebert, Lille/FR*
*J.M. Lee, Seoul/KR*
*M. Lewin, Villejuif/FR*
*O. Lucidarme, Paris/FR*
*M. Maas, Amsterdam/NL*
*A. Madureira, Porto/PT*
*M. Maher, Cork/IE*
*D. Malone, Dublin/IE*
*T. Mang, Vienna/AT*
*V. Maniatis, Sønderborg/DK*
*D. Marin, Durham, NC/US*
*L. Martí-Bonmatí, Valencia/ES*
*C. Matos, Lisbon/PT*
*Y. Menu, Paris/FR*
*G. Morana, Treviso/IT*
*G.H. Mostbeck, Vienna/AT*
*S. Mulé, Creteil/FR*
*E. Neri, Pisa/IT*
*A. Palkó, Szeged/HU*
*P. Paolantonio, Rome/IT*
*N. Papanikolaou, Lisbon/PT*
*S. Pötter-Lang, Vienna/AT*
*R. Pozzi Mucelli, Verona/IT*
*P. Prassopoulos, Thessaloniki/GR*
*E. Quaia, Padua/IT*
*G.A. Rollandi, Genoa/IT*
*M. Ronot, Clichy/FR*
*W. Schima, Vienna/AT*
*S. Schmidt Kobbe, Lausanne/CH*
*A. Schreyer, Regensburg/DE*
*O. Seror, Bondy/FR*
*S. Skehan, Dublin/IE*
*M. Staunton, Cork/IE*
*S. Stojanovic, Novi Sad/RS*
*J. Stoker, Amsterdam/NL*
*A. Taibbi, Palermo/IT*
*B. Taouli, New York, NY/US*
*J.P. Tasu, Poitiers/FR*
*S.A. Taylor, London/UK*
*S. Terraz, Geneva/CH*
*D. Tolan, Leeds/UK*
*C. Triantopoulou, Athens/GR*
*V. Valek, Brno/CZ*
*F. Vernuccio, Palermo/IT*
*V. Vilgrain, Clichy/FR*
*M.-P. Vullierme, Clichy/FR*
*M. Wagner, Paris/FR*
*D. Weishaupt, Zurich/CH*
*G. Zamboni, Verona/IT*
*C.J. Zech, Basel/CH*
*M. Zins, Paris/FR*

European Society

ESGAR

Gastrointestinal and Abdominal Radiology

**9:00 - 10:30** **Room E 102**

*Scientific Session SS 1*

### Optimising assessment of chronic liver diseases

### *SS 1.1*

### T1 relaxation times of the liver and spleen to predict significant liver fibrosis: is there an additional value of normalisation to blood pool?

V. Obmann, A. Christe, A. Berzigotti, L. Ebner, C. Gräni, A.T. Huber; Bern/CH

**Purpose:** To analyze liver and spleen native T1 relaxometry values to predict significant fibrosis and their additional value when normalized to the blood pool.
**Material and methods:** 156 patients without solid liver lesions underwent liver MR elastography (MRE) and shortened modified look-locker inversion recovery (shMOLLI)-based T1 relaxometry. T1 relaxation times were measured in the right liver lobe, in the spleen, in the aorta and in the vena cava. MRE liver stiffness was compared with T1 relaxation times alone and normalized to the blood pool in the vena cava and in the aorta using Pearson correlation and Student's t test. Prediction of significant fibrosis (based on an MRE cutoff value of 3.5kPa) was analyzed using receiver operation characteristics (ROC) analysis.
**Results:** Correlation between T1 relaxometry values and MRE liver stiffness was r=0.49-0.59 (p<0.001) for T1 of the liver and for T1 of the liver normalized to blood pool, while T1 of the spleen was less useful (r=0.11-0.17). Both normalized and not normalized T1 values of the liver allowed to significantly separate patients with significant liver fibrosis from those without significant liver fibrosis (p<0.001). In ROC analysis, T1 relaxometry values normalized to the blood pool did not perform better than T1 values alone.
**Conclusion:** Native T1 relaxation times of the liver allowed predicting clinically significant liver fibrosis, while T1 relaxation times of the spleen were less useful. There was no additional value of liver and spleen native T1 relaxometry values to predict significant fibrosis when normalized to the blood pool.

### *SS 1.2*

### Prevalence of significant hepatic fibrosis using MR elastography in a health check-up clinic population

K.A. Kang; Seoul/KR

**Purpose:** Using MR elastography (MRE), we investigated the prevalence of significant fibrosis in a community-based cohort.
**Material and methods:** From January 2015 to May 2018, 2,170 participants receiving a health examination at our institution were enrolled in this study. All participants underwent MR chemical shift technique and MRE to assess liver fat and fibrosis, respectively. We also conducted subgroup analyses for participants with nonalcoholic fatty liver disease (NAFLD), metabolic syndrome, and diabetes mellitus (DM). For generalization, sex- and age-standardized prevalence estimates of hepatic fibrosis and fatty liver were also calculated based on the Korean Statistical Information Service (KOSIS) during 2015-2018.
**Results:** The prevalence of F2 (≥3.0 kPa) and F3 fibrosis (≥3.6 kPa) in the overall cohort was 5.1% and 1.3%, respectively (sex- and age-adjusted prevalence of 3.8% and 1.3%). NAFLD prevalence (>5% fat fraction) was 20.8% in the average-risk population (after excluding alcohol use and viral hepatitis), and the prevalence of significant and advanced fibrosis in NAFLD participants was 7.5% and 1.1%, respectively. The prevalence of DM was 7.7% in the overall cohort, with significant fibrosis in 13.7% and advanced fibrosis in 4.8%. In participants with fatty liver (of any etiology) plus diabetes, 27.5% had ≥F2 and 7.2% ≥F3. Multivariate analyses indicated that older age, insulin, diabetes, and fatty liver were independently associated with significant fibrosis.
**Conclusion:** In a health check-up clinic setting, the prevalence of significant and advanced fibrosis was 5.1% and 1.3% in (sex- and age-adjusted prevalence of 3.8% and 1.3%, respectively) and increased three- to fourfold with diabetes.

### *SS 1.3*

### Prognostic role of spleen volume measurement using CT in patients with compensated chronic liver disease from hepatitis B viral infection

D.H. Lee, J. Yoo; Seoul/KR

**Purpose:** To determine prognostic value of spleen volume in patients with compensated chronic liver disease (cCLD) from chronic hepatitis B (CHB) viral infection.
**Material and methods:** A total of 584 patients were retrospectively enrolled. The cumulative incidence (CI) of development of decompensation, HCC occurrence, and overall survival (OS) were estimated by Kaplan-Meier method. Spleen volume was measured using semi-automated 3D volumetric software. The Cox proportional hazard regression model was used to evaluate prognostic factors. To obtain the optimal cut-off spleen volume to predict each outcome, minimal p value approach method was used.
**Results:** After median follow-up of 92 months, 114 patients developed HCC, and estimated 7-year CI of HCC occurrence was 17.2%. A larger spleen volume was a significant predictor of HCC occurrence (hazard ratio [HR]=1.01, p=0.007). Decompensation occurred in 30 patients with 7-year CI of 5.0%, and larger spleen volume was also significantly associated with the development of decompensation (HR=1.01, p<0.001). Twenty-three patients died, and their estimated 7-year OS was 96.4%. A larger spleen volume also significantly affected OS (HR=1.01, p=0.038). The optimal cut-off spleen volume was set at 532 mL for HCC occurrence (7-year CI: 15.9% of smaller volume vs. 25.4% of larger volume, HR=2.13, p=0.001), 656.9 mL for the development of decompensation (7-year CI: 3.7% of smaller volume vs. 19.9% of larger volume, HR=7.33, p<0.001), and 741.1 mL for OS (7-year OS: 97.8% of smaller volume vs. 86.4% of larger volume, HR=4.51, p=0.007).
**Conclusion:** A larger spleen volume was significantly associated with HCC occurrence, the development of decompensation, and poor OS in patients with cCLD from CHB.

### *SS 1.4*

### Comparison of liver stiffness measurement with MR elastography and liver and spleen volumetry for the prediction of disease severity and hepatic decompensation in patients with primary sclerosing cholangitis

I.S. Idilman[1], H.M. Low[2], S.K. Venkatesh[3]; [1]Ankara/TR, [2]Singapore/SG, [3]Rochester, MN/US

**Purpose:** The aim of this study was to evaluate liver stiffness measures (LSM) with MR elastography (MRE) and volumetry measurements of liver and spleen and their correlation with disease severity and prediction of hepatic decompensation.
**Material and methods:** This retrospective study was approved by the institutional review board. MRI and MRE studies were reviewed and mean liver stiffness of the, total liver, right lobe, left lobe and caudate lobe, and spleen volumes were calculated. Qualitative evaluation of lobar atrophy or hypertrophy and the presence of macronodular regeneration (MNR) was recorded. Statistical analysis was performed to evaluate correlations between LSM, volumetry measurements and Mayo risk score. Univariate and multivariate analyses were performed to predict hepatic decompensation.
**Results:** A total of 266 patients with PSC were included in the study. Lobar stiffness measures were higher in the presence of relative lobe atrophy. Mean LSM was higher in the presence of MNR. Significant correlations were observed between mean LSM and volumetry measurements with a fair correlation between LSM and spleen volume (rs=0.526, p<0.0001). Among the measurements, the best correlation was observed between mean LSM and Mayo risk score (rs=0.646, p<0.0001). In the multivariate analyses, mean LSM and Mayo risk score were significantly associated with the liver decompensation (hazard ratio, 1.18; 95%CI, 1.02-1.36 and hazard ratio, 1.65; 95%CI, 1.08-2.53, respectively).
**Conclusion:** LSM with MRE performs significantly better than liver and spleen volumes for the prediction of both disease severity and hepatic decompensation.

### SS 1.5

### True diffusion parameter of intravoxel incoherent motion MRI in hepatic fibrosis staging: we need a new approach

M. Gulbay, B. Sayin, D.T. Ocalan; Ankara/TR

**Purpose:** Hepatic myofibroblasts mediate both angiogenesis and fibrogenesis in cirrhotic process, and perfusion change accompanies diffusion restriction. The true diffusion (D) parameter is calculated using b values higher than 200 s/mm2 to filter perfusion effect in intravoxel incoherent motion MRI. However, no relationship between increased hepatic fibrosis severity and parameter D was found in previous studies using perfusion filtering. The aim of this study was to evaluate the efficacy of D parameter calculated with no filtering for perfusion and to compare the results with the classical method.
**Material and methods:** Respiratory triggered single-shot spin echo-EPI using 12b values (0-1000) was performed before the biopsy procedure in 37 chronic HBV patients that liver biopsy indicated. Patients were grouped as mild (stages F0-F1), moderate (stages F2-F3), and marked (stages F4-F5) fibrosis according to Ishak staging. D parameter maps were generated using all ($D_{0\text{-}1000}$) and only higher than 200 s/mm$^2$ ($D_{200\text{-}1000}$) b values. A 10-mm ROI was placed in the biopsy site in joint decision with interventional radiologist in three consecutive slices to calculate mean D value.
**Results:** D0-1000 showed better inverse correlation than D200-1000 with Ishak stages (Spearman's Rs= -0.685 and -0.485, respectively). All groups in D0-1000 and only mild and marked fibrosis groups in D200-1000 were significantly different (p<0.05, Tukey HSD). In terms of <F2 (mild) and ≥F4 (severe) disease discrimination, AUC was calculated as 0.848 and 0.935 for D0-1000 and 0.744 and 0.786 for $D_{200\text{-}1000}$, respectively.
**Conclusion:** D, calculated without filtering perfusion effect, is an effective and accurate parameter for demonstrating the severity of hepatitis B-induced liver fibrosis.

### SS 1.6

### Performance of liver surface nodularity for the differentiation between porto-sinusoidal vascular disease and cirrhosis

A. Calendra, R. Sartoris, P.-E. Rautou, A. Plessier, V. Vilgrain, M. Ronot; Clichy/FR

**Purpose:** To assess the performance of liver surface nodularity (LSN) quantification for the differentiation between porto-sinusoidal vascular disease (PSVD) and cirrhosis.
**Material and methods:** The study included patients from 2011 to 2018 with histologically proven cirrhosis (n=104) or PSVD (n=52) matched for severity who underwent hepatic CT or MRI during the initial diagnostic workup. Performance of LSN for diagnosing PSVD was assessed using a receiver operating characteristic curve (ROC) analysis. Student's t test and ANOVA were used.
**Results:** Patients were mostly men (110, 71%), with a mean age of 56±10 yrs (range 29-79). Overall, PSVD patients had a lower LSN value than patients with cirrhosis (2.70±0.63 vs. 3.23 ± 0.74, p<0.001). AUROC of LSN was 0.71±0.04. A cutoff value of 2.73 had a specificity of 74% for the diagnosis of PSVD. Stratifying cirrhosis patients by Child-Pugh score, LSN of patients with PSVD was lower in Child A (p=0.002) or Child B (p=0.005) patients, but not in Child C (p=0.40). Spleen diameter was significantly higher in patients with PSVD (mean 160 ± 47 vs. 138 ± 30mm, p<0.001). Combining LSN<2.73 and splenomegaly increased the diagnostic performance (AUROC=0.79±0.04).
**Conclusion:** LSN quantification is a promising new tool for the differentiation between PSVD and cirrhosis, especially in patients with compensated disease. Association of LSN and splenomegaly appears to improve the diagnostic performance.

### SS 1.7

### The performance of transient elastography for the detection of advanced fibrosis in non-alcoholic fatty liver disease

M. Duczkowska, M. Wawrzynowicz-Syczewska, J. Miętkiewski, K. Jurczyk; Szczecin/PL

**Purpose:** With obesity on the rise, non-alcoholic fatty liver disease (NAFLD) has become one of the most common liver diseases worldwide. The detection of advanced liver fibrosis is key in prognosis and long-time management. Large patient volume and the inarguable drawbacks of liver biopsy have led to the development of newer diagnostic techniques for the assessment of hepatic fibrosis. The aim of this study was to evaluate the accuracy of transient elastography for the diagnosis of advanced liver fibrosis and cirrhosis in NAFLD.
**Material and methods:** Forty-two patients with NAFLD were enrolled in this study. All subjects had satisfactory liver biopsy specimens and reliable liver stiffness measurements (LSM). The diagnostic performance of TE was determined using ROC curve analysis. Optimal cut-off points with the highest Youden index for the diagnosis of F >= 3 and F = 4 fibrosis were selected.
**Results:** For transient elastography, the calculated AUROCs for the detection of both advanced fibrosis and cirrhosis were 0.971 (95% CI = 0.928-1) and 0.984 (95% CI = 0.952-1), respectively. The optimal cut-off points were 8.9 kPa for F >= 3 (sensitivity 100%, specificity 90.3%) and 13.4 kPa for F = 4 (sensitivity 100%, specificity 94.3%).
**Conclusion:** Transient elastography has an excellent diagnostic accuracy for the detection of both advanced fibrosis and cirrhosis in NAFLD. It can be successfully used to rapidly identify patients with a higher risk of liver-related complications.

### SS 1.8

### Quantitative MRI characterisation of non-alcoholic steatohepatitis in a dietary rodent model

M. Dioguardi Burgio[1], P. Garteiser[1], F. Julea[2], A. Abyzov[2], V. Paradis[1], V. Vilgrain[1], B. Van Beers[1]; [1]Clichy/FR, [2]Paris/FR

**Purpose:** To assess the diagnostic performance of quantitative MRI parameters for the diagnosis of early non-alcoholic steatohepatitis (NASH) in a dietary rodent model.
**Material and methods:** We included 74 mice (C57bl6) with 2 control groups fed with normal diet (for 5 and 17 weeks, N=7), 3 dietary groups (5, 11 and 16 weeks, N=10) fed with high-fat diet deficient in choline and supplemented with methionine and 3 dietary groups (5, 11 and 16 weeks, N=10) fed with high-fat diet. The liver was scanned with a 7T system. Proton density fat fraction (PDFF), R2*, mechanical properties at 400, 600 and 800Hz including storage modulus (G'), loss modulus (G"), damping ratio, shear modulus (G*) and G* frequency dispersion coefficient were obtained with dedicated imaging sequences. At histopathology, the grade of steatosis, ballooning, inflammation, and the fibrosis stage were assessed. NASH was diagnosed according to the FLIP algorithm. Receiver operating characteristic (ROC) analysis, Spearman correlations and multivariate regression were used to test the variables.
**Results:** Nineteen mice had NASH including 15 with stage 1 fibrosis. Thirty-two had simple steatosis. G" at 400Hz had the largest AUROC for NASH (0.84; p<0.01) and for diagnosing NASH versus simple steatosis (0.80; p<0.01). Cutoff of G">0.38 kPa had 89% sensitivity and 79% specificity for NASH diagnosis. PDFF correlated with steatosis % (rho 0.81; p<0.01). In multivariate analysis, inflammation was the only determinant of the frequency dispersion coefficient.
**Conclusion:** At quantitative MRI, the loss modulus had the best diagnostic performance for diagnosing early NASH. Frequency dispersion coefficient might be a marker of liver inflammation.

### SS 1.9

### 2D shear wave elastography for significant liver fibrosis in patients with chronic hepatitis B: a systematic review and meta-analysis

H. Wei, H.-Y. Jiang, B. Song; Chengdu/CN

**Purpose:** To determine the diagnostic performance and cutoff of 2D shear wave elastography (2D SWE) for detecting significant liver fibrosis in patients with chronic hepatitis B (CHB).
**Material and methods:** A systematic literature search of the PubMed, EMBASE, Cochrane Library databases and Web of Science was conducted. Bivariate modelling and summary receiver-operating characteristic (ROC) modelling were constructed to summarize the diagnostic performance of 2D SWE. Meta-regression analyses were performed to explore the source of heterogeneity.
**Results:** Eleven eligible studies with 2623 patients were included. 2D SWE showed a summary sensitivity of 88% (95% CI: 83–91), specificity of 83% (95% CI: 78–88) and area under the ROC curve (AUC) of 0.92 (95% CI: 0.89-0.94) for detecting significant fibrosis in CHB patients. The mean cutoff of 2D SWE was 7.91 kPa (range: 6.73-10.00 kPa). Notably, the cutoffs of studies excluding patients with a history of prior antiviral therapy were generally lower than that of studies without excluding those who had received antiviral treatment, with an average of 7.15 kPa and 8.87 kPa, respectively (p < 0.01). Meta-regression analysis revealed that enrollment of consecutive patients was the only significant factor influencing heterogeneity (p < 0.01). Specifically, studies recruiting consecutive patients with CHB had significantly lower sensitivity than those with the absence of consecutive enrolment (0.83 vs 0.92, p < 0.01).
**Conclusion:** 2D SWE is an excellent modality for the prediction of significant liver fibrosis in CHB populations. Further work is required to establish the cutoffs that account for antiviral treatment as a potential confounding factor.

### SS 1.10

### CT and MRI features of non-cirrhotic portal fibrosis in comparison with liver cirrhosis: emphasis on focal hepatic lesions

J.H. Kang, D.H. Kim, S.Y. Kim, J.B. Lee, K.W. Kim, S.S. Lee, J. Choi, Y.-S. Lim; Seoul/KR

**Purpose:** To describe CT and MRI features of non-cirrhotic portal fibrosis (NCPF) in comparison to liver cirrhosis (LC), and to describe the characteristics of focal hepatic lesions detected in NCPF.
**Material and methods:** In this retrospective case-control study, 43 patients with pathologically proven NCPF (24 men; mean age, 50.7 years) from 2005 to 2018 were included. The control group with pathologically proven LC consisted of 129 patients (97 men; mean age, 51.7 years) matched with liver function. We evaluated the presence of liver surface nodularity and grades of portal hypertension, and compared them between the two groups using generalized linear mixed model. In addition, the presence and characteristics of focal hepatic lesions detected in NCPF were analyzed.
**Results:** Most patients with NCPF (36/43, 83.7%) showed no liver surface nodularity, while the majority of patients with LC (115/129, 89.2%) showed nodularity (P < 0.001). Grade 2 or 3 portal hypertension was significantly higher in the NCPF group than in the LC group (P ≤ 0.003). In the NCPF group, six patients (14.0%) had at least one focal hepatic lesions with arterial hyperenhancement. The most common pathologic diagnosis (4/6, 66.7%) was focal nodular hyperplasia (FNH). No malignant lesion was detected during the follow-up period (mean, 60.7 months).
**Conclusion:** In patients with portal hypertension, the absence of liver surface nodularity with higher grade portal hypertension is a suggestive feature of NCPF rather than LC. Benign focal lesions, most commonly FNH, were commonly accompanied in NCPF.

**9:00 - 10:30** **Room E 105 / 106**

*Scientific Session SS 2*

### CT colonography and colorectal cancer imaging

### SS 2.2

### Performance and evaluation in CTC screening: a cluster randomised trial

A. Obaro[1], D. Burling[1], R. Baldwin[2], C. Ugarte-Cano[1], P. Bassett[1], S. Halligan[3], A. Plumb[3]; [1]Harrow/UK, [2]Middlesex/UK, [3]London/UK

**Purpose:** To determine if a structured CTC training programme for experienced CTC readers increases interpretation accuracy.
**Material and methods:** We recruited representative, experienced CTC radiologists (median: 500-999 CTCs interpreted) who interpret CTC in their routine practice (median: 151 to 200 cases/year) from 72 NHS hospitals. We cluster randomised them into intervention (one-day training plus regular feedback) or control (no training or feedback) groups. Participants' sensitivity for colorectal cancer (CRC) and 6mm+ polyps was tested at 0, 1, 6 and 12 months post-training (intervention group) or post-enrollment date (control group), via interpretation of 10 CTC examinations with endoscopic validation. The primary outcome was the difference in sensitivity for 6mm+ polyps at the 1-month test between arms, analysed using analysis of covariance (ANCOVA). Secondary outcomes include diagnostic sensitivity at 6 and 12 months.
**Results:** 139 radiologists were randomised (intervention: 79; control: 60); currently, data regarding the primary outcome are available for 89 (due to ongoing follow-up). Mean baseline sensitivity was similar between the two groups (intervention: 49.7% ±17.4%; control: 45.1% ±13.4%, p=NS). At 1 month post-intervention, sensitivity was significantly greater in the intervention arm than the control group (intervention: 66.3% ±16.8%; control: 46.8% ±19.7%; difference = 16.9% [95%CI 9.9 to 23.8%], p<0.001). This effect persisted at 6 months (intervention: 65.9% ±14.6%; control: 55.3% ±16.5%; difference = 10.0% [95%CI 2.4 to 17.6%], p=0.01).
**Conclusion:** An easily administered intervention of one-day training with regular ongoing feedback significantly increases experienced CTC radiologist sensitivity for 6mm+ polyps, an effect sustained to at least 6 months.

### SS 2.3

### CTC post-imaging colorectal cancer rate in a large single-centre retrospective study: using flythrough may reduce the rate of interval cancers

T.M. Osborne, V. Shah, K. Mulcahy, R. Ahmad, J. Mullineux, S. Jepson, V. Billimoria, R. Verma, J.A. Stephenson; Leicester/UK

**Purpose:** To determine the rate of CTC post-imaging colorectal cancer (CRC) in a large teaching hospital in the United Kingdom and to examine possible contributing factors in 'missed' cancer cases.
**Material and methods:** All CTCs performed in our centre between January 2014 and September 2016 were retrospectively reviewed. The local cancer registry was used to determine the number of CTC-diagnosed CRCs during the study period, and the number of post-imaging CRCs in 36 months of follow-up.
**Results:** Over the study period, 4878 CTCs were performed on a total of 4797 patients. CTC detected 173 CRCs. Over 36 months of follow-up, we found 12 post-imaging CRCs. Thus, the post-imaging CRC rate was 6.49 (95% CI 3.40 - 11.10) per 100 cancers, and 2.46 (1.27 - 4.29) per 1000 CTCs. In retrospect, nine lesions were present on the index examination, two had sub-optimal CTC examinations, and one case was not visible on retrospective review. In cases of missed CRC, a) the initial study was not reported using flythrough and b) mean reporting times were significantly lower than the global mean.
**Conclusion:** We suggest that routine use of virtual flythrough when reporting CTC may reduce the rate of interval colorectal cancers. In sub-optimal studies, the report should be explicit that a repeat examination should be considered.

### SS 2.4

### Low-volume reduced bowel preparation for CTC: a randomised controlled trial

N. Panvini[1], D. Bellini[1], M. Rengo[1], S. Vicini[1], I. Carbone[1], A. Laghi[2]; [1]Latina/IT, [2]Rome/IT

**Purpose:** To investigate the feasibility and patient tolerance of a reduced bowel preparation for CTC.
**Material and methods:** Asymptomatic and symptomatic patients were enrolled in this multicentric randomised trial. All patients were randomly assigned (1:1 ratio, blocks of ten) to receive a reduced (52.5 g of Macrogol dissolved in 500 mL of water, RBP) or full (105 g of Macrogol in 1000 mL, FBP) bowel preparation and faecal tagging. Five readers performed a blinded subjective image analysis, by means of four-point Likert scales from 0 (highest score) to 3 (worst score). Endpoints were the quality of large bowel cleansing and tolerance to the assigned bowel preparation regimen.
**Results:** Seventy-eight patients were randomly allocated to treatments (44 in FBP group, 34 in RBP group). Both groups resulted in optimal colon cleansing. Homogeneity of fluid tagging (median score 0 vs 0, p=0.075), volume of residual stools (median score 0 vs 0, p=0.082), and colonic distension (median score 0 vs 0, p=0.073) were similar for both groups. RBP resulted in better patient tolerance.
**Conclusion:** Reduced bowel preparation may provide better tolerance for patients undergoing CTC without affecting colon cleansing and image quality.

### SS 2.5

### Faecal immunochemical test for colon cancer and its implications on symptomatic CTC requests

J.A. Stephenson, T.M. Osborne, V. Shah, R. Verma, B. Singh, D. Barnes; Leicester/UK

**Purpose:** Due to overwhelming burden on local colonoscopy services, we have used straight-to-test (STT) CTC as the primary investigation for patients >60 years old referred from primary care with change in bowel habit (CIBH) since 2016. This led to a significant increase in the numbers of CTC performed. In line with national guidance, we now use faecal immunochemical test (FIT) to triage patients prior to investigation. We assess the impact of FIT on CTC referrals.
**Material and methods:** FIT test was introduced in February 2018 and symptomatic patients with CIBH and a FIT <4ug/ml did not progress to CTC for exclusion of colon cancer. We compared the number of CTC requests from the STT pathway and from outpatients pre- and post-implementation of FIT.
**Results:** The mean number of total CTC performed per month pre-FIT was 307 (range 256-340) dropping to 263 (212-320) post-implementation (p = 0.0041). The number referred under the STT pathway was 213 (161-248) dropping to 142 (111-183) (p = 0.0001); however, there was a corresponding rise in the number of non-STT referrals from outpatients 87 (69-111) to 111 ( 86-152) (p = 0.0127).
**Conclusion:** FIT has the potential to reduce the burden on secondary care investigations to exclude bowel cancer. Our experience has shown that a FIT level of <4ug/ml reduced the number of STT referrals by ~25%. A FIT level at 10ug/ml would lead to a reduction of ~75% with significant beneficial cost and service provision impact. Interestingly, we have seen corresponding increases in non-STT referrals for CTC, a number of which had, had a negative FIT.

### SS 2.6

### The impact of mismatch repair status on the preoperative staging of local colon cancer

E. Erbs, S. Rafaelsen, J. Lindebjerg, L.H. Jensen, T.F. Hansen; Vejle/DK

**Purpose:** CT scan is standard in preoperative local staging of colon cancer. Tumours with a deficient mismatch repair (dMMR) system are characterised by unique clinical and pathophysiologic aspects that may impact the accuracy of the preoperative CT staging.
**Material and methods:** Data from the Danish Colorectal Cancer Group national clinical database addressing a cohort of patients operated for stage I-III colon cancer during 2010-15 were analysed. The analyses of MMR status had been conducted consecutively through means of immunohistochemistry. All CT scans were blindly assessed by a certified radiologist.
**Results:** Data from 590 patients, operated at a specialised cancer centre, were available for analyses. 135 (22.9%) of the patients had tumours demonstrating dMMR. The overall correlation of the clinical and pathological T-category was significant for both groups. There was an inferior correlation between clinical (cN) and pathological (pN) N-category (p>0.05) in pMMR cancers with a higher degree of over-staging assessed by CT scan compared to a significant correlation between cN and pN stages in pMMR cancers (p<0.01). Of the 91 dMMR tumours assessed node-positive by the preoperative CT scan, 59 (64.8%) showed no sign of metastatic involvement at the postoperative assessment.
**Conclusion:** Preoperative CT staging of dMMR colon cancer seems to overestimate nodal involvement and two-thirds of the patients preoperatively judged node-positive are false positive. The overall clinical value of a routine assessment of MMR status in the diagnostic setup is evident and the current study argues for implications to treatment decisions as well.

### SS 2.7

### Positron emission tomography may be useful to stage patients with rectal cancer and lateral pelvic lymph nodes with a short axis of at least 5 mm

C.D. Ortega, M.A. Queiroz, A. Naves, M.S. Rocha, S.C. Nahas, C.A. Buchpiguel; São Paulo/BR

**Purpose:** To verify the minimum size of lateral pelvic lymph nodes (LPLNs) that can be considered metastatic by positron emission tomography (PET) when integrated 18F-fluorodeoxyglucose (FDG)-PET/MRI is used to evaluate patients with rectal cancer at baseline staging.
**Material and methods:** IRB-approved retrospective analysis of prospectively collected data between Nov 2016 and Apr 2018 of 99 consecutive patients with rectal cancer at baseline staging (NCT02537340) was done. Inclusion criteria: >18 years with biopsy-proven rectal adenocarcinoma. Exclusion criteria: previous treatment, known neoplasia, PET/MRI contraindications, failure to consent and unavailable images. Images were reviewed in consensus by radiologist with 10years of experience in reading MRI and nuclear medicine physician with 3years of experience in reading PET. Assessment included a) MRI-identified LPLNs with malignant features (mixed signal intensity/border irregularity); b) the short axis (SA) of the largest LPLNs showing no malignant features in internal, external iliac or obturator compartments. Volume of interest (VOI) was drawn over the selected LPLNs to measure SUV; c) PET-identified LPLNs, considered metastatic when SUVmax was higher than 2.5. The SA of PET-positive nodes was recorded. The median node size was calculated.
**Results:** Fourteen patients (30 nodes) were positive on both PET and MRI components, five (5 nodes) were MRI-positive and PET-negative and two (2 nodes) were PET-positive and MRI negative. Median SUVmax uptake of PET-positive nodes was 4.9(2.6-17.6). Median size of PET-positive nodes was 10mm (5-18mm). Median size of MRI-positive nodes was 10mm (5-18mm). Twenty patients had no visible LPLN. Fifty-eight remaining patients had metabolic and morphological negative nodes with median size of 5.1mm (2-8mm).
**Conclusion:** The smallest PET-positive node had a short axis of 5mm.

### SS 2.8

### Prediction value of CT texture analysis to differentiate V-Ki-ras-2 Kirsten rat sarcoma viral oncogene homolog mutation status in colorectal cancer

F. Landolfi, G. Guido, D. Caruso, E. Pilozzi, E. Iannicelli, A. Laghi; Rome/IT

**Purpose:** To investigate the value of CT texture analysis in the prediction of V-Ki-ras-2 Kirsten rat sarcoma viral oncogene homolog (KRAS) mutation status in patients with lung metastases from colorectal cancer.
**Material and methods:** Eighteen patients with pathologically proven lung metastases from colorectal cancer were retrospectively enrolled. All patients underwent contrast-enhanced CT before the resection of metastases and KRAS mutation testing was performed on surgical specimen. Each metastasis was manually segmented from portal venous-phase CT images by an expert radiologist and analyzed with a dedicated software (TexRAD Ltd, Somerset, UK), which extrapolated the following texture parameters: mean, standard deviation of the pixel histogram (SD), skewness, kurtosis, mean value of positive pixel (MPP) and entropy. Mean value of texture parameters was calculated for each spatial spacing factor (SSF 0-6) and compared with KRAS mutation status. P values <0.05 were considered statistically significant.
**Results:** Nine patients (50%) had mutant KRAS and nine patients (50%) had wild-type KRAS. SD, entropy, MPP and kurtosis resulted significantly different (SD: p = 0.0016; entropy: p = 0.042; MPP, p = 0.0025; kurtosis, p = 0.05) between mutant and wild-type patients at medium and high filter levels (SSF 4-6). Mean and skewness showed no significant differences between the two groups of patients.
**Conclusion:** Texture parameters are significantly different between mutant and wild-type patients; texture analysis, providing a quantitative assessment of tumor microenvironment, may represent a non-invasive tool in the early prediction of KRAS mutation status in patients with metastatic colorectal cancer, allowing to customize treatment according to the predicted outcome.

### SS 2.9

### Diagnostic performance of dedicated rectal CT for staging rectal cancer: comparison with high-resolution rectal MRI and histopathology

S.H. Kim, S.Y. Son, Y.S. Suh, J.H. Yoon, B.Y. Hur, J.S. Bae; Seoul/KR

**Purpose:** To evaluate the diagnostic performance of dedicated rectal CT compared with high-resolution rectal MRI and histopathology in assessing rectal cancer.
**Material and methods:** Sixty-seven rectal cancer patients, who underwent dedicated rectal CT with rectal distension using 80-100ml of sonographic gel and high-resolution MRI within a month, were enrolled. For rectal CT, axial, coronal, and sagittal images were acquired with 3-mm reconstruction interval. For CT and MRI, the following items were analyzed by an experienced gastrointestinal radiologist: distance from anal verge and anorectal junction, shortest distance to mesorectal fascia, extramural depth (EMD), extramesorectal lymph node (LN) involvement, extramural venous invasion (EMVI), and T and N stages. CT and MRI findings were compared. Of 67 patients, 20 underwent radical surgery without concurrent chemoradiation therapy for whom CT findings were also compared with histopathology. Interclass correlation (ICC) and kappa statistics were used for statistical analysis.
**Results:** For all patients, distance from anal verge and anorectal junction showed strong correlation between CT and MRI (correlation coefficient, 0.972 and 0.955) (P<0.001). For EMD (cut-off: 5mm), threatened circumferential resection margin (cut-off: <2mm), LN metastasis, extramesorectal LN, and EMVI, kappa values between CT and MRI were 0.944, 0.931, 1.000, 0.892, and 0.884, respectively (P<0.001). For T stage, a weighted-kappa value was 0.954 (P<0.001). For 20 patients who received surgery, CT showed good agreement for T staging with histopathologic staging (kappa value, 0.865) (P<0.001). For involvement of CRM, EMVI, and LN metastasis, rectal CT showed acceptable concordance rates with histopathology in 75% (15/20), 95% (19/20), and 60% (12/20), respectively.
**Conclusion:** Dedicated rectal CT shows acceptable diagnostic performance comparable with MRI and histopathology for evaluating rectal cancers.

**11:00 - 12:30** **Room E 102**

*Scientific Session SS 3*

### Diagnosis and staging of pancreatic cancer: new trends

### SS 3.1

### Staging of pancreatic ductal adenocarcinoma: comparison between CT and MRI for accurate clinical practice

A. Lucarelli[1], A. Drudi[1], A. Beleù[1], A. Giaretta[1], G. Rizzo[1], G. Giannotti[1], F. Urraro[2], M. D'Onofrio[1]; [1]Verona/IT, [2]Caserta/IT

**Purpose:** To compare CT and MRI for the staging of pancreatic ductal adenocarcinoma (PDAC); assessing differences between the evaluations of radiologists with different degrees of expertise.
**Material and methods:** 38 patients with PDAC were prospectively enrolled between May 2018 and February 2019. All patients underwent contrast-enhanced CT and MRI performed close to each other, both before and after chemotherapy treatment. Main quantitative and qualitative CT and MRI parameters have been evaluated by two radiologists with different degrees of expertise in pancreatic imaging. Concordance between CT and MRI was tested both pre- and post-chemotherapy. Each CT parameter was compared with the corresponding MRI, considering one of them as the reference standard. Cohen's K-coefficient (k) was calculated to evaluate the inter-observer agreement.
**Results:** CT and MRI agree in the evaluation of tumor size (p<0.01), with a mean discrepancy of 15%. Tumor dimension in diffusion-weighted imaging (DWI) was correlated to the CT lesion diameter with a variation within 15%. Concordance in the assessment of vessel involvement and retroperitoneal infiltration was good (p<0.01), higher for the more experienced radiologist. In the evaluation of hepatic metastases, there was 94% concordance, with differences between the two radiologists (k=0.59, p<0.01). CT and MRI reported good correlation in the assessment of lymphadenopathies at expert's evaluation. An excellent correlation was observed between the two methods in the evaluation of healthy pancreatic parenchyma (p<0.01). Concordance values between CT and MRI were generally higher for the more experienced radiologist.
**Conclusion:** In PDAC staging, the pancreatic expert radiologist can use CT and MRI alternately, while a less skilled radiologist may prefer their parallel use.

### SS 3.2

### Prediction of survival in pancreas cancer patients who underwent surgery after neoadjuvant FOLFIRINOX therapy using CT and histopathological findings

S.J. Park, J.H. Kim, I. Joo; Seoul/KR

**Purpose:** To investigate important factors for recurrence-free survival (RFS) and overall survival (OS) in pancreas cancer with neoadjuvant therapy.
**Material and methods:** 69 patients with pancreas cancer underwent FOLFIRINOX therapy before surgery were retrospectively included. All patients performed baseline and follow-up CT. Two reviewers assessed CT findings. They also graded extra-pancreatic perineural invasion (EPI) using a 3-point scale focused on 5 routes. Clinical characteristics and histopathological results including tumor regression, using College of American Pathology (CAP) grade system, were investigated as well. Kaplan-Meier methods were used for RFS and OS. Cox proportional hazard regression model was used to find out significant predictive factors.
**Results:** 57 patients (82.6%, 57/69) had no residual tumor (R0) and 12 patients (17.4%, 12/69) had residual tumor (R1 or R2). Median RFS was 13 months (range 0–22). The RFS rates for 1 and 2 years were 53.1% and 35.9%, respectively. For RFS, EPI in the baseline CT (odds ratio (OR) 2.53, 95% CI: 1.116–5.733, p=0.026) and tumor regression grade (OR 1.76, 95% CI: 1.000–3.076, p=0.046) were important predictors of early recurrence in multivariate analysis. Mean OS was 48 months (range 11–35). The OS rates for 1 and 2 years were 98.5% and 72.5%, respectively. For OS, tumor regression grade (OR 1.05, 95% CI: 1.251–6.559, p=0.013) was significant factor. However, there were no independent predictors for residual tumor in CT findings.
**Conclusion:** EPI in the baseline CT was an important prognostic factor for tumor recurrence. Especially, CAP was an important prognostic factor for not only RFS but also OS.

Scientific Sessions

Authors' Index

### SS 3.3

### Pancreatic cancer diagnosis in patients with chronic pancreatitis with a new approach of mathematical postprocessing 128-row MDCT results

K.A. Lesko, J.V. Kuleznewa, D.S. Bordin, N.V. Orlova, E.A. Dubtsova, B.S. Nikitin, E.Y. Tyulyaeva, L.V. Vinokurova; Moscow/RU

**Purpose:** To explore the diagnostic value of a new approach of pancreatic cancer (PC) diagnosis in patients with chronic pancreatitis (CP) by mathematical postprocessing of 128-row MDCT results.
**Material and methods:** We analyzed results of contrast-enhanced MDCT from 122 patients with CP: 71 (58.2%) males and 51 (41.8%) females, mean age 53.7±13.03 (30-83), who underwent MDCT from January 2018 to October 2019. All cases were pathologically proved by core biopsy or surgery. We calculated the median of enhancement gradient in Hounsfield units (HU) between the region of interest and intact parenchyma (Mgrad) based on multiphase MDCT results. Correlation was assessed by Pearson's correlation coefficient (r). The receiver operating characteristic (ROC) curves were performed to evaluate the diagnosis potential of Mgrad.
**Results:** There were 46 (37.7%) patients with PC and 76 (62.3%) patients with only CP. Mean Mgrad value was higher in PC group—26.5±8.6 HU—than in CP group—13.9±5.1 HU (p=0.0001); Mgrad cut-off value for PC was 20 HU (p=0.001). There was positive correlation of Mgrad in patients with PC (r=0.68, p=0.0001) and negative correlation in patients with CP (r=-0.68, p=0.0001). Sensitivity, specificity, accuracy, positive and negative predictive value of PC diagnosis were 89.1%, 96.1%, 93.4%, 93.2%, 93.6% for Mgrad and 69.5%, 92.1%, 83.6%, 84.2%, 83.3% for MDCT, respectively; area under ROC for Mgrad was higher (0.927) than for MDCT (0.863).
**Conclusion:** Mgrad assessment based on MDCT results is more effective than only MDCT for PC diagnosis in patients with CP. Mgrad value positively correlates with PC existence; therefore, it could be useful for PC diagnosis.

### SS 3.4

### MRI screening in hereditary pancreatic cancer: findings in early pancreatic cancer

B. Boekestijn[1], S. Shahbazi Feshtali[2], M. Wasser[1]; [1]Leiden/NL, [2]Oegstgeest/NL

**Purpose:** In CDKN2A-p16-Leiden mutation carriers with 15-20% lifetime risk of pancreatic ductal adenocarcinoma (PDAC), annual MRI surveillance is able to detect PDAC at an early stage. In a retrospective study, findings on different MRI sequences were analysed, including a T1-weighted turbo field echo (TFE) with inversion recovery.
**Material and methods:** Sixteen subjects were included with PDAC detected during an observation period from January 2012 until March 2019 out of 218 mutation carriers enrolled in the program since 2000. MRI at 3.0T included T2-weighted, fat-suppressed T1-weighted dynamic contrast-enhanced (DCE), MRCP and T1-TFE sequences. Diffusion-weighted imaging (DWI) was added in 2015. Two readers independently analysed the current and past MRI examinations for lesions and other findings.
**Results:** Fourteen of 16 PDAC were seen on T1-TFE and T1-DCE by both readers. Thirteen tumors demonstrated increased enhancement in the delayed phase of T1-DCE. Dilatation of the pancreatic duct was present in 5 subjects. One PDAC was in proximity to an intraductal papillary mucinous neoplasm. DWI was available in 11 subjects, with restricted diffusion in 7 subjects. In 9 of 13 subjects with an incident tumor, a measurable lesion was detected on previous imaging. The average growth rate of those lesions was 6.7 mm/year (s = 3.0) with an estimated volume doubling time of 157.5 days (s = 62.7).
**Conclusion:** Knowledge of imaging features and recognition of subtle changes could improve detection of hereditary pancreatic cancer in a screening setting. Relatively slow growth rates of PDAC justify short-term follow-up in uncertain MRI findings.

### SS 3.5

### Detection of extrapancreatic perineural invasion in pancreatic head ductal adenocarcinoma: utility of four MDCT patterns of spread

A. Ivanovic, Belgrade/RS

**Purpose:** To determine sensitivity and specificity of four MDCT patterns of perineural spread in pancreatic ductal adenocarcinoma: linear, reticular, mass-like and nodular.
**Material and methods:** This study included 50 adults (28 men and 22 women, mean age, 66 years; range 44–88 years) with pancreatic head mass who underwent MDCT. Three blinded radiologists independently reviewed each MDCT exam and four patterns of perineural spread were assessed: (1) linear, (2) reticular, (3) mass-like and (4) nodular. Sensitivity, specificity, and positive and negative predictive values of the patterns were calculated, as well as interobserver agreement.
**Results:** Sensitivity, specificity, and positive and negative predictive values of the "linear" pattern of perineural spread for the three readers were 25–51%, 77–85%, 54–78% and 71–81%, respectively ($\kappa$ = 0.51); for the "reticular" pattern, they were 48–63%, 82–90%, 56–88%, and 75–78%, respectively ($\kappa$ = 0.6); for the "mass-like" pattern, they were 52–71%, 85–92%, 64–87%, and 62–68%, respectively ($\kappa$ = 0.64) and for the "nodular" pattern, they were 21–24%, 66–72%, 52–75%, and 32–35%, respectively ($\kappa$ = 0.44).
**Conclusion:** The "reticular" and "mass-like" MDCT patterns have high specificity for detecting extrapancreatic perineural invasion in PDAC.

### SS 3.6

### Audit and service evaluation: improving outcomes in pancreatic cancer surgery: does the use of a standardised reporting template improve report quality in pancreatic and peri-ampullary malignant tumours in a tertiary referral centre?

R.M. Hodnett, R.A.K. Ibrahim, G. Miles, M. Puckett, E.M. Armstrong, S. Aroori; Plymouth/UK

**Purpose:** At our tertiary hepato-pancreatico-biliary surgical centre, imaging of pancreatic and periampullary malignancy is performed and reported at patients' local hospitals before referral, and reviewed for multi-disciplinary team meeting (MDTM) by specialist GI radiologists at our centre. There is resultant heterogeneity of imaging and reporting practice. We assess whether using a reporting template reduces heterogeneity and improves report quality and, ultimately, patient outcomes.
**Material and methods:** This audit was registered with our centre's audit and assurance team; ethical approval was not required. A surgical database search identified all surgically managed patients with confirmed diagnosis of pancreatic/peri-ampullary malignancy over 18 months. The CT imaging available at time of decision to operate was anonymised; reports were audited against a modified National Comprehensive Cancer Network® (NCCN) reporting template. New reports were produced by two experienced GI radiologists utilising the same template and compared to the original reports. $\kappa$-values for interobserver relatability were calculated, and statistical significance assessed using Student's t test.
**Results:** 59 consecutive patients (37 male, 22 female), mean age 66 (36-83), were managed surgically during this period. Histology confirmed 49 adenocarcinoma (ductal 25, periampullary 8, unspecified 19), 6 neuroendocrine tumour, and 1 mixed adenocarcinoma/neuroendocrine. Original reports (n=59) contained mean key features ± standard deviation of 5.05±1.94 (range 1-9). Template reports (incomplete data, n=13) contained 13.69±0.63 features (range 12-14), P<0.005). $\kappa$-values and full results to follow.
**Conclusion:** Use of template reporting resulted in more complete and accurate disease evaluation and is likely to have improved interobserver relatability. It is, therefore, likely to facilitate better surgical planning and improve patient outcomes.

### SS 3.7

### Can CT texture analysis predict resectability and surgical outcomes after neoadjuvant radiotherapy in pancreatic cancer patients?

R. De Robertis, N. Cardobi, N. Simoni, S. Paiella, P. Capelli, R. Mazzarotto, S. Montemezzi, M. D'Onofrio; Verona/IT

**Purpose:** To evaluate changes in texture analysis parameters of CT images after adjuvant stereotactic body radiation therapy (SBRT) in patients with locally advanced pancreatic adenocarcinoma.

**Material and methods:** Pre- and post-SBRT contrast-enhanced CT examinations of 54 patients with locally advanced pancreatic adenocarcinoma were retrospectively evaluated. 3D regions of interest were manually drawn on arterial- and portal-phase CT images to comprise the whole tumor; 47 texture parameters were analyzed. Baseline and post-SBRT tumor volumes and texture parameters were compared between groups (resected versus non-resected and R0 versus R1-2 resection) using Wilcoxon test.

**Results:** Forty patients (72.7%) underwent surgical resection after SBRT; among them, 25 had R0 resection (62.5%). Baseline tumor volume was not significantly different between groups (p>.05). Resected and R0 patients had significant tumor volume change after SBRT compared to non-resected and R1/2 patients (-39.7% vs +22.6%, p=.005; and -47.1% vs -17.3%, p=.017). Texture analysis identified 31 arterial-phase parameters and 27 portal-phase parameters significantly different before and after SBRT in resected tumors (p<.05). Among resected tumors, 22 arterial-phase parameters and 28 portal-phase parameters were significantly different after SBRT compared to baseline (p<.05).

**Conclusion:** Texture analysis of contrast-enhanced CT images can identify changes after SBRT in locally advanced tumors that could be predictive of resectability and R0 resection.

### SS 3.8

### MR in the identification of mural nodules of intraductal papillary mucinous neoplasms of the pancreas

G. Giannotti, G. Tedesco, A. Beleù, N. Cardobi, R. De Robertis, M. D'Onofrio; Verona/IT

**Purpose:** To evaluate the diagnostic accuracy of MRI with diffusion-weighted imaging (DWI) sequences in the identification of mural nodules in pancreatic intraductal papillary mucinous neoplasms (IPMN).

**Material and methods:** 91 preoperative MRI scans with histopathological diagnosis of IPMN were reviewed by two radiologists. The following parameters were evaluated: presence, number and size of mural nodule, lesional signal intensity on T1-WI after contrast administration and on DWI (b 800 s/mm2), size of the cystic lesion and dilation of MPD. Each case was reviewed by two pathologists. They evaluated presence, number, size of papillary structures and the degree of epithelial dysplasia. Qualitative and quantitative analyses were performed. Inter-observer agreement was calculated.

**Results:** Statistically significant differences (p<0.0001) were found for the following correlation: presence of nodules >5mm and confirmation by pathological review, size and number of mural nodule evaluated on pathological review and the degree of dysplasia, size and number of mural nodule evaluated and tumoral dysplasia, presence of nodule >5mm with enhancement and hyperintensity on DWI and the degree of dysplasia, dilation of MPD and tumoral dysplasia. Inter-observer agreement was moderate for the presence of mural nodule (K=0.56), the presence of high signal intensity on DWI (K=0.60) and enhancement of mural nodule (K= 0.58). Histogram analysis of ADC map showed a correlation between entropy of the entire cystic lesion and the degree of dysplasia (p<0.0001).

**Conclusion:** Dynamic MRI with DWI has good accuracy in the detection of IPMN mural nodule >5mm. DWI and T1-WI after contrast medium administration can be useful in the evaluation of malignancy of IPMN.

### SS 3.9

### Incidentally detected pancreatic duct dilation on abdominal US: assessment of yield of subsequent MRCP

A.F. Bezuidenhout, K.J. Mortele; Boston, MA/US

**Purpose:** The clinical significance and current management of patients with an incidentally detected dilated pancreatic duct (PD) on abdominal US with no visualized underlying cause are not clear. Our purpose was to assess the diagnostic yield of subsequent MRCP and to identify additional predictors that may be associated with underlying pancreaticobiliary malignancy.

**Material and methods:** We included consecutive patients in whom MRCP was performed for further evaluation of an incidentally detected dilated PD on abdominal ultrasound from 2011 to 2017. We reviewed MRCP scans and retrieved clinical data retrospectively from our hospital information system. Histology or clinical follow-up was used as the reference standard. Chi-square test was performed to determine the association between categorical variables.

**Results:** 58 patients were identified with mean age of 65 years, 35 (60%) being female. 36/58 (62%) had PD dilation on MRCP, the others did not. In 17/36 (47%) patients, a specific pancreaticobiliary cause for PD dilation was identified on MRCP [7 (19%) malignant and 10 (28%) benign]. In the remaining 19/36 (53%) patients, no cause was identified. On bivariate analysis, patients with associated common bile duct (CBD) dilation were more likely to have underlying pancreaticobiliary malignancy (p = .002). No patient with initial negative MRCP had pancreaticobiliary malignancy on subsequent workup.

**Conclusion:** Incidentally detected PD dilation on US is not always due to benign causes. MRCP is an effective non-invasive method for identifying the underlying cause in these patients and in detecting life-threatening pancreaticobiliary malignancy. Patients with associated CBD dilation are more likely to have underlying pancreaticobiliary malignancy.

### SS 3.10

### Prediction of early recurrence after surgery in patients with pancreatic neuroendocrine tumour using preoperative MRI

S. Han, J.H. Kim, S. Jang, J. Yoo, J.K. Han; Seoul/KR

**Purpose:** To investigate important MRI features for predicting early recurrence in patients with pancreatic neuroendocrine tumor (PNET) after surgery.

**Material and methods:** A total of 100 patients (mean age 55.8 years; M:F, 49:51) with PNET who underwent MRI and first-line surgery from 2000 to 2018 were included. Two radiologists independently assessed MRI findings including size, location, margin, T1- and T2-signal intensity, enhancement patterns, CBD and pancreatic duct dilatation, vascular invasion, LN enlargement, DWI, ADC value, retrospectively. Survival and clinicopathologic data including underlying disease, tumor grade, TNM stage, resection margin and postoperative complications were collected. Image findings associated with disease-free survival and overall survival were assessed with Kaplan-Meier survival analysis and multivariate Cox proportional hazard regression analysis.

**Results:** The mean disease-free survival and overall survival of the patients were 115.3 [96.8-133.9] and 130.0 [119.4-140.7] months, respectively. Among the variables, arterial iso- to hypo-enhancement, portal iso- to hypo-enhancement, ductal dilatation, arterial invasion, venous invasion, lymph node enlargement, larger tumor size and higher histologic grade showed significant early recurrence (p< 0.05) and poor overall survival (p< 0.05) in univariate analysis. In multivariate analysis, portal iso- to hypo-enhancement (HR 23.12 [2.72-196.4] (p=0.004)), ductal dilatation (HR 4.76 [1.13-19.95] (p=0.033)), arterial invasion (HR 72.13 [4.63-1123.31] (p=0.002)), venous invasion (HR 6.35 [1.80-22.43] (p=0.004)) and tumor size (HR 1.03 [1.00-1.07] (p=0.046)) showed significant effect on early recurrence. However, there was no significant variable for overall survival.

**Conclusion:** MRI features including size, enhancement pattern, vascular invasion and ductal dilatation are useful in predicting early recurrence after surgery in patients with PNET.

**11:00 - 12:30** **Room E 105 / 106**

*Scientific Session SS 4*

### Imaging gastro-intestinal neoplasms: new insights

### SS 4.1

### Performance of CT imaging features for the identification of high-risk GI stromal tumours

R. Cannella[1], E. Tabone[2], C. Gozzo[1], G. Cappello[2], G. Porrello[1], D. Regge[2], T.V. Bartolotta[3]; [1]Palermo/IT, [2]Turin/IT, [3]Cefalù/IT

**Purpose:** To investigate the diagnostic accuracy of CT imaging features for the identification of high-risk GI stromal tumors (GISTs).
**Material and methods:** This retrospective dual-institution study included patients with pathologically proven GIST meeting the following inclusion criteria: i) preoperative contrast-enhanced CT performed between 2007 and 2019; ii) lack of neoadjuvant treatment; iii) pathological analysis through resection specimens. Tumor risk stratifications were determined according to the National Institutes of Health (NIH) 2008 criteria. Two radiologists evaluated the CT imaging features, including enhancement pattern and tumor characteristics in a blinded fashion. The distribution of CT features between high-risk and low-to-intermediate-risk GISTs was assessed using univariate and multivariate binary logistic regression analyses. Statistical significance was set at $p<0.05$.
**Results:** The final population included 86 patients (58 men, 28 women, mean age 60.1±10.9 years) with 37 high-risk and 49 low-to-intermediate risk GISTs (mean diameter 8.6±6.2 cm). High-risk GISTs demonstrated more frequently heterogeneous enhancement (91.9% vs 44.9%, $p<0.001$), lobulated contours (86.5% vs 32.7%, $p<0.001$), ill-defined margins (37.8% vs 0%, $p<0.001$), exophytic growth (73.0% vs 49.0%, $p=0.018$), intralesional necrosis (67.6% vs 32.7%, $p=0.001$), cystic degeneration (10.8% vs 0%, $p=0.031$), intratumoral vessels (37.8% vs 6.1%, $p<0.001$), and enlarged feeding vessels (67.5% vs 4.1%, $p<0.001$) compared to low-to-intermediate-risk GISTs. At multivariate analysis, lobulated contours (OR: 0.12, 95% C.I.: 0.03-0.78, $p=0.023$) and enlarged feeding vessels (OR: 16.07, 95% C.I.: 1.89-136.19, $p=0.010$) remained independently associated with high-risk GIST.
**Conclusion:** Morphologic contrast-enhanced CT features are significantly different depending on the risk status and may help to predict patients with high-risk GIST.

### SS 4.2

### CT texture analysis of GI stromal tumours

I. Ambrosini[1], G. Cappello[1], E. Tabone[1], A. Timpani[1], R. Cannella[2], C. Gozzo[2], V. Giannini[1], A. Defeudis[1], T.V. Bartolotta[2], D. Regge[1]; [1]Turin/IT, [2]Palermo/IT

**Purpose:** To evaluate the association between radiomic biomarkers extracted from baseline CT imaging, mitotic count, tumor mutational profile and prognostic Miettinen classification.
**Material and methods:** This retrospective multicenter observational study includes 63 histologically proven gastrointestinal stromal tumors (GISTs). Each lesion was manually segmented; 37 texture features were extracted either on a single slice and on the entire tumor volume. Reference standards: pathological findings and Miettinen classification. Patients were dichotomized with mitotic count (≤5/50HPF vs >5/50HPF), mutational status (c-KIT mutation vs PDGFRα and wild-type), patients prognosis (good prognosis class: none, very low and low risk vs poor prognosis class: intermediate and high risk). Univariate analysis using the Mann-Whitney test and multivariate analysis were performed; a stepwise logistic regression model was developed to predict patient's prognosis using 70% of patients as the training set and the remaining 30% as the test set.
**Results:** Eight 3D features discriminated lesions with low or high mitotic count (best AUC 0.81, best sensitivity 86%, best specificity 93%). Six 3D parameters detected GISTs based on the mutational group (best AUC 0.77, best sensitivity 75%, best specificity 79%) and three parameters correlated with risk class (best AUC 0.76, best sensitivity 72%, best specificity 85%). To differentiate between GIST at lower or higher risk of recurrence, the regression model used 6 different features with AUC 0.78, sensitivity 65%, specificity 79%, VPN 71% and VPP 73% on the training set, and AUC 0.83, sensitivity 88% and specificity 75% on the test set.
**Conclusion:** A good correlation between radiomics features, disease aggressiveness, mutational profile and risk of recurrence was observed. Results are promising; validation on external datasets is necessary to confirm the role as imaging biomarker.

### SS 4.3

### Imaging differentiation of intraabdominal desmoid tumour from peritoneal seeding in patients with a previous history of cancer surgery

S.H. Kim, J. Suh, H.-J. Kang; Seoul/KR

**Purpose:** To investigate whether there are differential imaging features of intraabdominal desmoid tumor from peritoneal seeding in patients with a history of previous cancer surgery.
**Material and methods:** From January 2000 to June 2019, 32 patients who had pathologically proven intraperitoneal lesions developed after cancer surgery were enrolled. There were 17 desmoid tumors and 16 peritoneal seedings. Portal-phase CT and/or positron-emission tomography (PET) findings were analyzed by two board-certified radiologists in consensus for the following items: size, shape, margin, degree of enhancement and fluorodeoxyglucose (FDG) uptake, homogeneity, presence of intralesional fat, necrosis, calcification, adjacent organ invasion, peritoneal thickening, and mass effect. For quantitative analysis, the Hounsfield unit (HU) of lesions and psoas muscles as well as maximum standardized uptake value (SUVmax) of the lesions were measured. Imaging findings were compared between desmoid tumor and peritoneal seeding groups using statistical analysis methods.
**Results:** Desmoid tumors frequently showed iso-attenuation (14/17) while peritoneal seeding depicted high attenuation (12/16) compared to psoas muscle ($P=0.001$). Intralesional fat was more frequently found in desmoid tumors (8/17) than in peritoneal seeding (1/16) ($P=0.017$). Desmoid tumors frequently showed well-defined margin (9/17) and smooth contour (13/17) whereas peritoneal seeding had ill-defined margin (13/16) and lobuating contour (11/16) ($P=0.041$ and 0.009, respectively). HU ratio between the lesion and psoas muscle was not significantly different between desmoid tumor (1.15) and peritoneal seeding (1.23) ($P=0.570$). SUVmax (4.14) of desmoid tumor did not significantly differ from that (5.19) of peritoneal seeding ($P=0.519$).
**Conclusion:** Desmoid tumor can be non-invasively differentiated from peritoneal seeding based on CT findings in patients with a previous history of cancer surgery.

### SS 4.4

### Exploring the value of (semi-)quantitative variables from baseline F18-fluorodeoxyglucose-positron emission tomography/CT and MRI for the prediction of locoregional failure after chemoradiotherapy in patients with anal carcinoma

L.A. Min, Y.J.L. Vacher, N.W. Schurink, L. Dewit, M. Donker, B. van Triest, W.V. Vogel, M. Maas, R.G.H. Beets-Tan, D.M.J. Lambregts; Amsterdam/NL

**Purpose:** To explore if (semi-)quantitative parameters from baseline 18F-fluorodeoxyglucose-positron emission tomography (FDG-PET)/CT and MRI may predict locoregional failure in anal carcinoma patients treated with chemoradiotherapy (CRT).
**Material and methods:** We retrospectively analyzed n=108 patients with anal squamous cell carcinoma that had undergone baseline staging PET/CT and/or MRI (T2W-MRI + DWI with three b-values up to b1000) and underwent CRT. Of these, 99 had PET/CT and 38 MRI (29 had both). A pelvic MR-expert delineated the anal tumours on T2W-MRI and DWI to calculate T2W-volume and DWI-volume. Metabolic tumour volume on PET was defined by a cutoff of 42% of the tumour's SUVmax (MTV 42%). The following additional parameters were calculated: SUVmax, SUVpeak, T2W-entropy, DWI-entropy and ADC-mean. Outcome was complete response (CR) versus locoregional failure (residual or recurrent tumour/nodes) within one year after CRT. Potential of each imaging parameter to predict CR vs. locoregional failure was explored using univariable logistic regression.
**Results:** Of the 99 patients in the PET analysis, 20 (20.2%) experienced a locoregional failure; of the 38 patients in the MRI analysis, this was 8 patients (21.1%). Of the PET variables, MTV 42% and SUVpeak were significantly associated with failure: odds ratio (OR) 1.03 (95%CI: 1.0-1.06; P= 0.014) and OR 1.15 (95%CI: 1.0-1.32, P=0.046). In the MRI analysis, only DWI entropy showed an association: inter-quartile OR 8.39 (95%CI: 1.14-61.92; P=0.037), albeit with a wide confidence interval.
**Conclusion:** Potential pre-treatment predictors of locoregional failure after CRT may be extracted from baseline staging PET/CT and MRI. However, considering the small patient series (especially for MRI), these findings clearly require further exploration and large-scale multivariate validation.

### SS 4.5

### Post-chemoradiotherapy assessment of anal carcinoma using MRI in biopsy-proven patients: preliminary results

R. Faletti, T. Perelli, M. Gatti, P. Fonio; Turin/IT

**Purpose:** To evaluate the role of MRI in local response assessment and detection of early local relapse after chemoradiotherapy (CRT) in patients with anal squamous cell carcinoma (AC).
**Material and methods:** Twenty-three patients with histologically proven AC who completed pre-(t0), post-CRT 4-week(t1) and 20-week(t2) MRIs and pre-(t0) and post-CRT 20-week(t2) PET scan were included. Tumor size (D, mm), volume (V, cc), apparent diffusion coefficient (ADC, mm2/s), time to peak (TTP, sec) and SUV were recorded. Lesion biopsy performed at 6 months was the gold standard test for the evaluation of response to treatments (responder, r, or non-responders, nr). Data were analyzed using non-parametric test and ROC curve.
**Results:** Twenty patients were classified as responders. In the responder group, all the parameters significantly changed (decrease of D, V and SUV; increase of ADC and TTP). Non-responders had significantly higher values of D and V than responders at any time: $D_{t0}$[nr 80mm vs r 50mm, p=0.03]; $D_{t1}$[nr 50mm vs r 26mm, p=0.02]; $D_{t2}$[nr 28mm vs r 4mm, p=0.03; $Vt_0$[180cc vs 35cc, p=0.008]; $V_{t1}$[nr 79cc vs r 4cc, p=0.006]; $V_{t2}$[nr 13cc vs r 0.13cc, p=0.01]. There were no significant differences of ADC, TTP and SUV in the two groups at any time. ROC curve identified as a threshold for responders: $V_{t0}\leq 120$ cc (AUC=0.99), $V_{t1}\leq 40$ cc (AUC=1.00), $\Delta V\%_{t1}\geq 80\%$ (AUC=0.925) and $D_{t1}\leq 40$ mm (AUC=0.92).
**Conclusion:** $V_{t0}\leq 120$ cc, $V_{t1}\leq 40$ cc, $\Delta V\%_{t1}\geq 80\%$ and $D_{t1}\leq 40$ mm seem to be markers of good response to treatment. After therapy, there is a significant increase of ADC and TTP in all patients, without a significant difference between the two groups: in the future, the addition of more non-responder patients could confirm this difference.

### SS 4.6

### Positron emission tomography/CT performed at 3 months following chemo-radiotherapy predicts outcomes in anal cancer

J. Mercer, H. Mistry, P. Manoharan, T. Westwood, A. Renehan, R. Kochhar; Manchester/UK

**Purpose:** Early salvage surgery after locoregional failure of chemoradiotherapy (CRT) for anal squamous cell carcinoma (ASCC) is associated with better outcomes. Previous data suggest equivocal findings on 3 months post-treatment MRI in more than half of cases, potentially delaying curative surgery or leading to unnecessary EUA and biopsy. We aimed to evaluate the effectiveness of 3m post-treatment positron emission tomography (PET)/CT in predicting outcomes in ASCC.
**Material and methods:** 257 patients were consecutively managed for ASCC from January 2012 to January 2018. With local ethical approval, reports for 18F-fluorodeoxyglucose (FDG)-PET/CT performed 3 months following CRT were placed into 5 tumour regression categories based on combined qualitative and semiquantitative assessment of anal canal metabolic activity. Outcome measures were overall survival (OS) and locoregional treatment failure/time to progression. Univariable and complete case multivariable landmark analyses, 3-month landmark time, of the covariates of interest were conducted using the Cox proportional hazards model. Presentation of survival curves using the Kaplan-Meier method and p-values from the log-rank test is reported.
**Results:** 243 patients were included. Patients who had residual disease at 3 months as suggested by the PET/CT Tumor regression grading (TRG) score and confirmed on EUA/biopsy had poorer prognosis. Multivariable analysis showed that PET/CT response was a strong predictor of locoregional failure (HR 7.14 (3.89-13.11), p<0.001) and overall survival (HR 7.55 (3.90-14.61), p<0.001).
**Conclusion:** FDG-PET/CT is highly accurate in assessing early response to treatment in ASCC, an excellent predictor of outcomes and streamline management pathways. This study supports the inclusion of PET/CT in response assessment for anal cancer.

### SS 4.7

### Segmentation of anal cancer on T2-weighted and diffusion-weighted MRI by radiologists and radiation oncologists: how does it affect tumour volumes?

L.A. Min[1], Y.J.L. Vacher[1], C. Sofia[2], L. Dewit[1], M. Donker[1], B. van Triest[1], P. Bos[1], M. Maas[1], R.G.H. Beets-Tan[1], D.M.J. Lambregts[1]; [1]Amsterdam/NL, [2]Messina/IT

**Purpose:** Use of diffusion-weighted imaging (DWI) (vs. T2W-MRI) has been shown to affect tumour segmentation and volumetry in different tumour types, including anal cancer. Segmentations in research are typically done by radiologists, while clinical segmentations (radiotherapy planning) are done by radiation oncologists. The aim was to evaluate how use of DWI vs. T2W-MRI affects tumour segmentation in anal cancer and compare results between radiologists and radiation oncologists.
**Material and methods:** The staging MRIs (T2W-MRI and b800-DWI) of 25 anal cancer patients were retrospectively analyzed by two radiologists and two radiation oncologists. Readers manually segmented the tumour volumes, first on T2W-MRI (without seeing the DWI) and subsequently on b800-DWI. Mean segmented volumes were compared between T2W-MRI and DWI and between readers. Inter-reader agreement was calculated using the Dice similarity coefficient (DSC).
**Results:** Overall, tumour volumes measured by the radiologists were smaller than those measured by the radiation oncologists, both on DWI (mean 18.7 vs 24.2 cm3; P=0.001) and on T2W-MRI (21.0 vs 24.3 cm3; P=0.04). Compared to T2W-MRI, mean tumour volumes on DWI were smaller for the radiologists (18.7 vs. 21.0 cm3; P=0.03), but not for the radiation oncologists (24.2 vs 24.3 cm3; P=0.58). Inter-reader agreement was good, with slightly better results for DWI vs. T2W-MRI, both for the radiologists (DSC 0.68 vs. 0.63) and radiation oncologists (DSC 0.70 vs. 0.68).
**Conclusion:** Segmentations by radiation oncologists result in significantly larger tumour volumes than segmentations by radiologists. Use of DWI may slightly improve inter-reader reproducibility and can lead to smaller volumes when segmented by radiologists.

### SS 4.8

### CT prediction of the margin-positive resection and poor survival of perihilar cholangiocarcinoma after surgery

H.-J. Kang[1], J.H. Kim[1], I. Joo[1], W. Chang[2], J.K. Han[1];
[1]Seoul/KR, [2]Seongnam-si/KR

**Purpose:** To evaluate preoperative CT findings to predict margin-positive resection and poor survival in perihilar cholangiocarcinoma.
**Material and methods:** From 2006 to 2012, 139 patients with perihilar cholangiocarcinoma who underwent preoperative multi-phase CT and subsequent curative-intent surgery were included. Two radiologists assessed CT findings including likelihood of bile duct (BD) involvement from intrapancreatic common bile duct (CBD) to bilateral second-order branches and peritumoral fat stranding using 5-point scale, vessel involvement (no, abutment, encasement), BD anatomy variation, parenchymal atrophy and lymph node (LN) involvement. Residual tumor categorized into complete resection (R0) and margin-positive resection (R1 or R2) based on histopathology. Chi-square test and logistic regression were used for analyzing predictive factors of margin-positive resection. Also, survival analysis by Kaplan-Meier analysis and Cox regression analysis were performed with CT findings and laboratory results.
**Results:** Seventy-one patients were R0 and sixty-eight patients were margin-positive resection. For resectability evaluation, mid-CBD involvement (hazard ration [HR] = 3.09, 95% CI: 1.44 - 6.67, p=0.004) in preoperative CT was a significant factor for margin-positive resection in multivariate analysis (P<0.01). In multivariate survival analysis, intrapancreatic CBD involvement (HR = 1.81, 95% CI: 1.03 - 3.16, p < 0.01) as well as elevated total bilirubin (HR = 1.53, 95% CI: 1.02 - 2.29, p < 0.01) and CA19-9 level (HR = 1.75, 95% CI: 1.12 - 2.65, p < 0.01) were significant predictors for poor survival.
**Conclusion:** Distal longitudinal extent of perihilar cancer on preoperative CT is a significant factor for margin-positive resection and poor survival on curative intent surgery.

### SS 4.9

### CT-based radiomics for the prediction of outcome after somatostatin analogues in patients with liver metastases of a neuroendocrine tumour: an exploratory study

F. Staal[1], M. Taghavi[1], M. van Treijen[2], M. Tesselaar[1], R.G.H. Beets-Tan[1], M. Maas[1]; [1]Amsterdam/NL, [2]Utrecht/NL

**Purpose:** To explore the value of CT-based whole-liver radiomics for response evaluation and grade assessment in patients treated with somatostatin analogues (SSAs) for liver-metastasized neuroendocrine tumours (NET).
**Material and methods:** Thirty-eight consecutive patients with pathologically confirmed small intestine or pancreatic NET (grade 1=24/grade 2=14), treated with SSAs underwent contrast-enhanced CT. The whole liver was semi-automatically delineated on arterial phase with Philips Intellispace Portal, excluding main vessels and bile ducts. Five histogram features were extracted with Pyradiomics, using different Laplacian-of-Gaussian (LoG) filters. Response (progressive disease (PD) vs. stable disease (SD) or partial response (PR)) was assessed with RECIST1.1. Response and grade (1 vs. 2) were compared with Mann–Whitney U or independent t test. Univariate Cox regression was used for overall survival (OS) analyses.
**Results:** 10/38 patients had PD (median follow-up 53 months) and 28 had PR/SD (median follow-up 65 months, p=0.065). Radiomics features did not significantly differ between patients with PD compared to patients with PR/SD. Kurtosis (unfiltered+LoG0.5) was significantly different between patients with grade 1 and 2 NET (p=0.05). Skewness, mean (LoG2.5) and kurtosis (LoG2.5) were significantly associated with OS (HR 0.76 (95%CI: 0.61-0.95); HR 1.96 (95%CI: 1.07-3.60) and HR 1.07 (95%CI: 1.02-1.12), respectively).
**Conclusion:** These preliminary data show no value for whole-liver CT-based radiomics to identify NET patients treated with SSAs that will show progression. Whole-liver CT-based radiomics might show the potential to identify patients at risk of impaired outcome (i.e. higher grade and OS). These findings need to be validated in a larger cohort and lesion-based analysis will be explored to investigate whether this leads to better results.

### SS 4.10

### Evaluation of predictive factors of radioembolisation-induced liver disease in patients with liver metastases treated with Y90-radioembolisation

R.H. Hayoz, A. Digklia, C. Dromain, A. Hocquelet, A. Denys, J. Prior, N. Schaeffer, J.-F. Knebel, R. Duran; Lausanne/CH

**Purpose:** Y90-radioembolization is increasingly used in cancer patients. Even though Y90-radioembolization demonstrated to be safe and effective, in some cases it may lead to the so-called radioembolization-induced liver disease (REILD) that is characterized as the occurrence of jaundice and ascites 1-2 months after treatment in patients without tumor progression or bile duct obstruction. The purpose of our study was to identify risk factors of REILD in patients with liver metastases treated with Y90-radioembolization.
**Material and methods:** A retrospective analysis of a prospectively collected database was performed. 116 patients with liver metastases (mean age: 66 years, range 24-91y) were treated with Y90-radioembolization (2010-2017). Demographics, radiological, functional and clinical data, number and treatment parameters of SIRT, and administered treatments (chemo-/immunotherapy, liver-directed locoregional therapies) were analyzed. The effect of those parameters was determined by univariate analysis.
**Results:** 78 patients were included in this ongoing analysis. Mean administered dose was 1.9UKq (0.3-5.87). The administered dose correlated with a significant increase in bilirubin and ASAT levels at 1 month post-SIRT (r=0.358, p=0.017 and r=0.39, p=0.006). A trend was observed for bilirubin at 3 months (r=0.288, p=0.068). 9 REILD were observed. Univariate logistic regression demonstrated that baseline y-GT was predictive of REILD (OR=1.302, 95%CI=1.045-1.66; p=0.022). None of the other investigated factors were predictive of the occurrence of REILD.
**Conclusion:** REILD is an uncommon but potentially severe complication that can happen in patients with liver metastases treated by Y90-radioembolization. Patients with elevated baseline y-GT are at increased risk of REILD.

**14:30 - 16:00** **Room E 102**

*Scientific Session SS 5*

### Machine learning and radiomics: current applications in GI imaging

### SS 5.1

### Radiomics machine-learning model for the prediction of local tumour progression after thermal ablation for colorectal liver metastases

M. Taghavi[1], F. Staal[1], F. Gómez Muñoz[2], E.G. Klompenhouwer[1], F. Imani[1], D. Meek[1], U.A. Van der Heide[1], R.G.H. Beets-Tan[1], M. Maas[1]; [1]Amsterdam/NL, [2]Barcelona/ES

**Purpose:** Knowledge about the risk of local tumor progression (LTP) after thermal ablation for colorectal liver metastases (CRLM) patients is critical to optimize percutaneous ablation results and for subsequent follow-up. The aim of this study was to develop and validate a machine-learning radiomics model to predict LTP based on pre-ablation CT in CRLM patients.
**Material and methods:** 93 CRLM patients (143 lesions) treated by means of ablation were included and divided into a training (n=65 patients, n=102 lesions) and validation (n=28 patients, n=41 lesions) set. The validation set was independent of the training set. After manual segmentation and preprocessing, 1,316 radiomics features were extracted for each lesion. Three predictive models were constructed based on (1) radiomics features, (2) clinical features and (3) a combination of clinical and radiomics features to predict two-year LTP-free survival (LTPFS). Bayesian-optimized gradient boosting with wrapper feature selection was trained on the training set and optimized with sequential model-based optimization for prediction models based on radiomics features.
**Results:** Median follow-up was 24 months (range 6-115). 26 patients had LTP in 32 lesions. The concordance_index in the validation set to predict LTPFS was (0.76; 95% confidence interval [CI]: 0.75-0.77, p=0.01 risk stratification) for the radiomics model, (0.60; 95%CI: 0.58-0.61, p=0.69; risk stratification) for the clinical model and (0.76; 95%CI: 0.75-0.77, p=0.01 risk stratification) for the combined model.
**Conclusion:** Machine learning-based predictive models incorporating pre-ablation radiomics features showed good prognostic potential and allowed significant stratification for LTPFS after thermal ablation in CRCLM patients.

### SS 5.2

### Combining radiomics and deep features in positron emission tomography improves accuracy in tumour response to neo-adjuvant chemoradiotherapy in oesophageal squamous cell carcinoma

J.T.J. Van Lunenburg, W.H.K. Chiu; Hong Kong/HK

**Purpose:** This study examined whether radiomics and deep learning are predictive of tumour response in patients with oesophageal squamous cell carcinoma (OSCC) treated by neoadjuvant chemoradiotherapy (nCRT) and surgery.
**Material and methods:** Ninety-five patients (65 training and 30 validation cohorts) who had undergone pre-treatment fluorodeoxyglucose positron emission tomography (18F-FDG-PET) studies were included and classified as those who achieved complete pathological response (pCR) and those who did not. The primary tumours were segmented using a fixed threshold approach and radiomics features extracted to construct a hand-crafted radiomics (HCR) signature. The contoured volume of interest (VOI) and the PET images were input into a fully convolutional neural network (CNN) for volumetric medical imaging (VNet) for the prediction of pCR. A third model was constructed combining both the radiomics and deep features.
**Results:** pCR was achieved in 36 (37.9%) patients. The HCR signature achieved an area under the receiver operating characteristic curve (AUC) of 0.787. We observed substantial overfitting in our deep learning model and instead, the CNN generated VOI of the primary tumour and 8 stable deep features. A single deep feature was shown to be predictive of pCR with an AUC of 0.741. The model was improved when both the HCR and deep features were combined (AUC 0.839).
**Conclusion:** HCR features provide incremental value to deep learning for predicting pCR in OSCC patients undergoing nCRT. Our approach not only has the potential to provide a personalised management plan for these patients but also demonstrates a novel approach in tackling small medical imaging datasets using deep learning.

### SS 5.3

### Radiomic analysis of hepatobiliary-phase primovist MRI is associated with disease-free survival in patients with surgically resectable colorectal liver metastases

J. Shur[1], M. Orton[1], A. Connor[2], S. Fischer[3], C.-A. Moulton[3], S. Gallinger[3], D.-M. Koh[4], K. Jhaveri[3]; [1]London/UK, [2]Durham, AL/US, [3]Toronto, ON/CA, [4]Sutton/UK

**Purpose:** Colorectal cancer with liver metastases (CRLM) is potentially curable with surgical resection; however, clinical prognostic factors can insufficiently stratify patients. This study aims to assess whether radiomic features from CT and MRI are prognostic and can inform clinical decision-making.
**Material and methods:** This single-site retrospective study included 102 patients who underwent CRLM resection with pre-operative CT and MRI with gadoxetic-acid (EOB). A lasso-regularized multivariate Cox proportional hazards model was applied to 3 sets of 104 radiomic features derived from the portal-venous CT, unenhanced T1-weighted fat-suppressed (T1FS) and hepatobiliary phase (HBP) data, respectively, to determine association with disease-free survival (DFS). A prognostic index was derived using the significant Cox regression coefficients and their corresponding input features and a threshold was determined to classify patients into high- and low-risk groups, and DFS compared using log-rank tests.
**Results:** Two radiomic co-variates were significantly associated with DFS; minimum pixel value (MIN) (HR=1.66, p=0.00016) and small area emphasis (HR=0.62, p=0.0013) from the EOB-MRI data. Radiomic T1FS and CT features were not prognostic. The prognostic index stratified high- and low-risk prognostic groups, although this was not significant (HR 0.251, p=0.096). MIN was positively associated with delayed tumour enhancement (r= 0.77, p< 2 x 10-16).
**Conclusion:** Radiomic HBP primovist MRI features are associated with DFS, but not those derived from CT or T1FS data, and are partly explained by delayed tumour enhancement, likely due to post-treatment tumour fibrosis. This merits further validation for potential clinical implementation to inform patient management.

### SS 5.4

### Deep learning for fully automated segmentation of rectal tumours on MRI in a multicentre setting

J. Van Griethuysen[1], N.W. Schurink[1], M.J. Lahaye[1], F. Bakers[2], R.F.A. Vliegen[3], R. Geenen[4], V. Cappendijk[5], S. De Bie[6], H.J.W.L. Aerts[7], R.G.H. Beets-Tan[1], D.M.J. Lambregts[1]; [1]Amsterdam/NL, [2]Maastricht/NL, [3]Heerlen/NL, [4]Alkmaar/NL, [5]'s-Hertogenbosch/NL, [6]Deventer/NL, [7]Boston, MA/US

**Purpose:** To explore the performance of deep learning to perform fully automated segmentation of rectal tumors on baseline MRI using a multicenter dataset (with variations in acquisition protocols and scan quality reflecting daily practice).

**Material and methods:** Baseline staging MRIs of 355 patients (from 6 centers) were analyzed. Overall scan quality was assessed using a 5-point Likert score (0=poor) to 4=excellent). Data were randomly split into train:validation:test cohorts (ratio of 5:1:4). An expert-radiologist manually delineated all rectal tumours to serve as training input. Using the T2-weighted, diffusion-weighted imaging (DWI)-b1000 and apparent diffusion coefficient (ADC)-maps from the training+validation cohorts, an attention-gated u-net was trained, with optimal hyperparameters (including learning rate, optimizer algorithm and batch-size) determined via grid search. A second expert-radiologist independently resegmented all patients in the test cohort to calculate inter-reader agreement using the dice similarity coefficient (DSC). Agreement (DSC) between the trained network and the expert-segmentations was compared to the expert inter-reader agreement (serving as the standard of reference).

**Results:** Average DSC between expert-readers was 0.75 (±0.18). Average DSC for the network-generated segmentations was 0.64 (±0.22) and 0.59 (±0.22) compared to expert-reader 1 and 2, respectively. When excluding poor quality (score ≤ 2) scans, DSC between the network and expert-readers increased to 0.68 (±0.17) and 0.63 (±0.17).

**Conclusion:** Despite large variations in scan protocol/image quality, deep learning networks achieved a promising overall performance to automatically segment rectal tumours on MRI. Better results were achieved after exclusion of poor-quality scans, with agreement levels up to 0.63-0.68 (versus 0.75 between expert-radiologists).

### SS 5.5

### Assessment of malignant potential in intraductal papillary mucinous neoplasms of the pancreas using MR findings and texture analysis

S.K. Jeon, J.H. Kim, J. Yoo, J.K. Han; Seoul/KR

**Purpose:** To investigate the usefulness of MR findings and texture analysis for predicting the malignant potential of pancreatic intraductal papillary neoplasms (IPMNs).

**Material and methods:** 248 patients with surgically confirmed IPMNs (106 high grade (HG; invasive carcinoma and high-grade dysplasia) and 142 low grade (LG; low/intermediate-grade dysplasia)) and who underwent preoperative MRI with MRCP were included. MR findings suggestive of high-risk stigmata or worrisome features based on the international consensus Fukuoka guidelines 2017 were analyzed. Quantitative features were extracted using texture analysis of T2-weighted MRCP. Multivariate analysis was used to identify independent predictors for HG IPMNs. Diagnostic performance was also analyzed using receiver operating curve analysis.

**Results:** Among MR findings, enhancing mural nodules ≥5mm, main pancreatic ductal (MPD) dilatation ≥10mm, and abrupt change of MPD with upstream parenchymal atrophy were significant predictors for HG IPMNs (all Ps <0.05). Among texture variables, the significant predictors for HG IPMNs were lower sphericity (P=0.004) and lower compactness (P<0.001). At multivariate analysis, enhancing mural nodule ≥5mm (odds ratios (ORs), 7.97; 95% confidence interval (CI), 4.10-15.52; P<0.001), MPD dilatation ≥10mm (OR, 2.59; 95% CI, 1.16-5.79; P=0.021) and lower compactness on texture analysis (OR, 0.81; 95 % CI, 0.67-0.98; P=0.032) were significant factors for predicting HG IPMNs. Addition of texture variable to MR findings showed better diagnostic performance for predicting HG IPMNs than using MR findings only (AUC, 0.83 vs. 0.79, P=0.008).

**Conclusion:** MRCP-derived texture features are useful for predicting malignant potential of IPMNs and addition of texture analysis to MRI features may improve diagnostic performance for predicting HG IPMNs.

### SS 5.6

### Reproducibility of radiomics in pelvic MRI: effect of variations between readers, segmentation methodology and software

N.W. Schurink, J. van Griethuysen, L.A. Min, R.G.H. Beets-Tan, S. van Kranen, D.M.J. Lambregts; Amsterdam/NL

**Purpose:** Although several studies have investigated the reproducibility of radiomics data derived from CT and PET/CT, data on the reproducibility of MR-based radiomics are scarce. This study assesses the reproducibility of radiomic features from pelvic MRI data and investigates the effects of variations between readers, segmentation methodology and software packages.

**Material and methods:** 25 pelvic MRIs (T2W-MRI of anal cancer) were retrospectively analyzed and segmented by two readers to include the [1] whole-tumour volume, and [2] largest single axial tumour slice. Pixel intensities were normalized to mean=300/SD=100, and images were resampled isotropically (2x2x2mm3). Radiomic features were extracted using 2 open-source packages (PyRadiomics-v2.2.0, CaPTk-v1.7.3), using comparable settings without image filtration. A fixed histogram bin of 5 was used. Only features defined in both packages were extracted (first-order, shape, GLCM, GLRLM, GLSZM and NGTDM features, 51 total). For each feature, the intra-class correlation coefficient (ICC) was calculated between the [1] two readers, [2] two segmentation methods (whole-volume vs. single-slice) and [3] two software packages.

**Results:** Inter-reader reproducibility was moderate (20/51 features; 0.5<ICC<=0.75) to good (15/51; 0.75<ICC<=0.9). Between segmentation methods, most features (in particular GLRLM, GLSZM, NGTDM) showed poor reproducibility (31/45; ICC<0.5), though first-order features showed good (7/15; 0.75<ICC<=0.9) to excellent (2/15; ICC>0.9) reproducibility. Between software packages, most first-order, shape, GLCM and GLRLM features showed excellent reproducibility (23/30; ICC>0.9). The remaining higher order features (GLSZM, NGTDM) were all poorly reproducible (21/21; ICC<0.5).

**Conclusion:** Variations in software and segmentation methodology negatively affected measurement reproducibility in MRI-based radiomics, especially higher order features. Inter-reader reproducibility was moderate-to-good.

### SS 5.7

### Gadoxetic acid-enhanced MRI radiomics signature: prediction of clinical outcome in HCCs after surgical resection

Z. Zhang, B. Song; Chengdu/CN

**Purpose:** To evaluate the efficiency of gadoxetic acid-enhanced MRI-based radiomics features for the prediction of overall survival (OS) in HCC patients after surgical resection.

**Material and methods:** 120 patients with surgically confirmed HCC were enrolled. Three different regions of interests were delineated on each phase with automatic and manual approach: 1) a region of the entire tumor ($ROI_{tumor}$); 2) a 1-cm-wide radius surrounding the tumor boundary ($ROI_{penumbra}$); and 3) a region of liver parenchyma excluding intratumor and peritumoural region ($ROI_{liver}$). Radiomics signatures (Rad-scores) were built from radiomics features in different ROIs with LASSO Cox regression analysis. Preoperative clinical characteristics and semantic imaging features potentially associated with OS were evaluated to develop a clinical-radiological model. Then the radiomics features and clinic-radiological predictors were integrated into a combined model using multivariable Cox regression analysis. Kaplan-Meier analysis and log-rank test were performed to compare the discriminative performance, and evaluated on the validation cohort.

**Results:** The combined Rad-score showed significant association with OS in both cohorts (all p< 0.001) and could successfully stratified patients into higher risk or lower risk groups. The Barcelona clinic liver cancer (BCLC) stage, arterial peritumoral enhancement and the combined Rad-score were independently associated with OS. Moreover, the combined model incorporating with clinical-radiological and radiomics features showed an improved predictive performance in estimation of OS (C-index, 0.92, 95%CI: 0.87-0.97), compared to the clinical-radiological model (C-index, 0.80, 95%CI: 0.72-0.88; p=0.039). Decision curve analysis revealed the combined model we constructed performed better than clinical-radiological and radiomics models.

**Conclusion:** Radiomics features along with clinical-radiological predictors can assist in preoperative HCC prognosis prediction and enable a step-forward precise medicine.

### *SS 5.8*

### Texture analysis of preoperative CT images of mass-forming cholangiocarcinoma: 2D and 3D texture analysis with disease-free survival

S. Park, J.M. Lee, J. Park, J.K. Han; Seoul/KR

**Purpose:** To determine whether CT texture analysis (CTTA) has a value in the prediction of disease-free survival (DFS) in patients with mass-forming type intrahepatic cholangiocarcinoma (mICC) undergoing surgical resection.

**Material and methods:** The late arterial-phase CT scans of 89 patients with mICC who underwent surgical treatment were retrospectively analyzed. CTTA was performed using a software (Radiomics, Syngo.via Frontier, Siemens Healthineers, Forchheim, Germany) that employed a first-order and second-order texture analysis by drawing a region of interest of 1) the largest cross-sectional area of the tumor (2D) and 2) whole tumor volume (3D). Patients were followed up until disease progression. Cox proportional hazard models were used to determine the relationship between texture features and DFS.

**Results:** Univariate analysis of 2D texture identified that first-order mean ($p=.001$), energy ($p=.037$), kurtosis ($p=.001$), and shape-flatness ($p=.006$) were significant univariate markers of DFS. Univariate analysis of 3D texture yielded mean ($p<.001$) as a significant factor. Among clinicopathologic parameters, size ($p <.001$), extrahepatic involvement ($p=.006$), multiplicity ($p=.016$), lymph node involvement ($p=.000$), and CEA ($p=.003$) were significant univariate markers. A Cox regression model including all significant univariate markers identified no significant texture factors on 2D analysis but first-order mean ($p=.006$) on 3D analysis. Size and lymph node (LN) involvement were significant factors on 2D and 3D analyses and CEA was a significant factor in 2D analysis on multivariate analysis.

**Conclusion:** The mean of the 3D texture parameters is independently associated with poorer DFS in patients with mICC, while other texture parameters did not show correlation with DFS.

### *SS 5.9*

### Influence of different adaptive statistical iterative reconstruction levels on CT radiomic features

F. Pucciarelli, T. Polidori, E. Muscogiuri, M. Zerunian, B. D'Arrigo, D. Caruso, A. Laghi; Rome/IT

**Purpose:** To evaluate the influence of different levels of adaptive statistical iterative reconstruction (ASIR) on CT radiomic features.

**Material and methods:** 38 patients who underwent unenhanced CT scans of the abdomen with the same scanner (Revolution Evo, GE Healthcare, USA) were analyzed. Subsequently, raw data of filtered backprojection (FBP) were reconstructed with 10 levels of ASIR (from 10 to 100). Two radiologists analyzed texture features of liver and kidney tissues using two different regions of interest (ROIs) that were cloned for all eleven different iteration level datasets. Data were elaborated with TexRad Medical Imaging Software. Six different radiomic features (mean, sd, entropy, mpp, skewness, kurtosis) were extrapolated and compared between FBP and all ASIR levels.

**Results:** Texture analysis of the liver revealed significant differences between FBP and all ASIR reconstructions for mean (all $p<0.002$), sd (all $p<0.0001$), entropy (all $p<0.0001$) and mpp (all $p<0.0001$), while no significant differences were observed for skewness and kurtosis between FBP and all ASIR reconstructions (all $p>0.45$ and all $p>0.58$, respectively). Similar results were obtained for kidney analysis with no significant differences for skewness and kurtosis (all $p>0.053$ and all $p>0.176$, respectively) and significant changes for mean (all $p<0.0001$), sd (all $p<0.0001$), entropy (all $p<0.0036$) and mpp (all $p<0.0001$).

**Conclusion:** No influence of iterative reconstruction algorithm was reported for skewness and kurtosis compared to FBP in liver and kidney analysis whereas mean, sd, entropy and mpp were significantly affected by ASIR. Skewness and kurtosis may be reliable quantitative parameters.

### *SS 5.10*

### Prediction of splenomegaly in >100,000 structured oncologic radiology reports using natural language processing

S. Sun[1], K. Lupton[2], K. Batch[2], N. Gangai[1], J. Cho[1], L. Gazit[1], H. Nguyen[1], F. Zulkernine[2], A.L. Simpson[2], R.K.G. Do[1]; [1]New York, NY/US, [2]Kingston, ON/CA

**Purpose:** To develop and assess the accuracy of a natural language processing (NLP) model to identify splenomegaly from structured CT radiology reports at a tertiary cancer center.

**Material and methods:** In an IRB-approved, retrospective study, all CT chest/abdomen/pelvis reports (July 2009 to April 2019) adhering to departmental structured template were included. The SPLEEN subsection was extracted and those with default 'unremarkable' text were excluded from training. For patients with colorectal cancers (CRC), hepatobiliary cancers (HB), leukemia, Hodgkin's lymphoma (HL) and non-HL (NHL), 1920 of 105,042 reports were annotated as positive or negative/uncertain for splenomegaly. Model training was performed on 1536 and model accuracy was tested on 384 reports. The prediction model was then applied to the remaining reports to calculate frequencies of splenomegaly.

**Results:** In the annotated reports, splenomegaly was present in 42.2%. After training, the splenomegaly classifier achieved 94% overall accuracy, 94.6% precision (positive predictive value) and 94% recall (sensitivity). When the model was applied to all unannotated reports, the predicted frequency of splenomegaly for CRC patients was 8.7% (5275/60462), HB 17.7% (2210/12506), leukemia 31.5% (1684/5340), HL 6.1% (390/6386) and NHL 9.2% (1866/20348).

**Conclusion:** NLP can predict splenomegaly from structured radiology reports after training from a limited sample of annotated text. At our institution, the frequency of splenomegaly in CRC patients was similar to HL and NHL patients, and lower than both patients with HB cancers and leukemia. Validation with splenic volumetry is ongoing.

9:00 - 10:30 Room E 102

*Scientific Session SS 6*

### Inflammatory bowel disease and luminal imaging

### *SS 6.1*

### MRI after ligation of the intersphincteric fistula tract for high perianal fistulas in Crohn's disease: a retrospective cohort study

K. Van Rijn[1], E.M. van Praag[1], M. Monraats[2], C. Buskens[1], J. Stoker[1]; [1]Amsterdam/NL, [2]Delft/NL

**Purpose:** Surgical closure of high perianal fistulas using ligation of the intersphincteric fistula tract (LIFT) procedure is increasingly used in Crohn's disease. Currently, data on postoperative MRI findings are lacking, while important to assess deep healing. Therefore, we aimed to evaluate fistula characteristics on MRI and the relation with clinical outcomes after LIFT procedure.
**Material and methods:** Consecutive Crohn's patients treated with LIFT between 2007 and 2018 for high perianal fistulas who underwent baseline and follow-up MRI were retrospectively included. MRIs were scored by two radiologists according to items based on the original and modified Van Assche indices. MRI findings and the relation towards clinical healing were described.
**Results:** Twelve patients were included (4 male, median age 34 years (IQR 28-39)). Follow-up MRI was performed, median 5.5 months (IQR 2.5-6.0) after LIFT. At this time, eight patients (67%) showed clinical healing, which increased to ten (83%) during follow-up, none had a recurrence. Three patients (25%) needed a re-intervention due to inflammatory masses and/or persisting fistulas. At baseline, all patients showed a tract predominantly filled with granulation tissue, which changed to fibrotic in seven (58%). All clinically responding patients showed a decrease in tract volume and/or hyperintensity with the absence of hyperintensity (T1 and T2) in four (33%) patients.
**Conclusion:** Clearly decreased fistula activity can be observed on MRI after LIFT surgery in Crohn's patients. A large proportion of patients develops a fibrotic tract relatively soon after LIFT and shows no clinical recurrences, suggesting a highly effective therapy and prognostic value of MRI.

### *SS 6.2*

### MR enterography before treatment may predict response to tumour necrosis factor-alpha inhibitors in Crohn's disease

J. Rimola[1], N. Capozzi[2], A. Fernandez-Clotet[1], J. Castro-Poceiro[1], I. Ordás[1], J. Panes[1]; [1]Barcelona/ES, [2]Bologna/IT

**Purpose:** Identifying predictors of therapeutic response is the cornerstone of personalized medicine. We aimed to identify predictors of long-term healing of severe inflammatory lesions detected at MR enterography (MRE) in patients with Crohn's disease treated with tumor necrosis factor-alpha (TNF-α) inhibitors.
**Material and methods:** Prospective longitudinal single-center study including patients with clinically active Crohn's disease requiring treatment with TNF-α inhibitors with at least one intestinal segment with a severe inflammatory lesion detected by MRE (segmental MaRIA ≥ 11). MRE data were obtained at baseline, week-14, and week-46. The primary endpoint was the healing of severe inflammatory lesions (MaRIA < 11) in each segment. The secondary endpoint was healing of all severe inflammatory lesions in all intestinal segments.
**Results:** We included 58 patients with 86 intestinal segments with severe inflammatory lesions. At week-46, healing of severe lesions was found in 51 (59.3%) segments, and complete healing of inflammatory all lesions in all segments was found in 28 (48.6%) patients. Multivariate analysis found predictors of healing colonic location (as opposed to ileal) (OR 4.5 [1.2-17.2]; p=0.024) and lack of creeping fat at MRE (OR 4.3 [1.7–17.6]; p=0.04). Healing on MRE at week-14 is an independent predictor of sustained healing at week-46 (OR 11 [2.1-57.1]; p=0.004).
**Conclusion:** In patients with Crohn's disease, the absence of creeping fat detected at pretreatment MRE and location of severe inflammatory lesions are clinically relevant predictors of long-term healing of severe inflammation after treatment with TNF-α inhibitors.

### *SS 6.3*

### Diffusion-weighted MR enterography: quantitative analysis segmental apparent diffusion coefficient values in normal and abnormal bowel segments in patients with Crohn's disease

J. Pilipovic Grubor, S. Stojanovic; Novi Sad/RS

**Purpose:** To evaluate the mean segmental apparent diffusion coefficient (ADC) values in normal and abnormal bowel segments and statistically analysed differences between them in Crohn's disease during MR enterography.
**Material and methods:** We performed a retrospective–prospective study in which standardized MR enterography evaluation was used on 50 patients with histopathology-confirmed diagnosis of Crohn's disease who had either symptomatic disease or were monitoring disease activity during treatment. MR enterography included DWI (b =1400 sec/mm$^2$). For each of the seven bowel segments (jejunum, proximal ileum, distal and terminal ileum, right colon, transverse colon, left colon and sigmoid colon and rectum) per patient (n=350), we quantitative analysed segmental normal and pathological ADC values and statistically analysed differences between them. We used MaRIA score as the reference standard.
**Results:** The mean ADC value for normal 265 segments was 1.95x10$^{-3}$mm$^2$/s and for 85 abnormal segments it was 1.14x10$^{-3}$ mm$^2$/s. There were significant differences in segmental ADC values between normal segments: the highest normal ADC values were in segment 2 (2.03x10$^{-3}$ mm$^2$/s), the lowest in segment 7 (1.79x10$^{-3}$ mm$^2$/s). The lowest pathological ADC values were in segment 3 (1.07x10$^{-3}$ mm$^2$/s). The statistical differences between segmental ADC values were higher in normal than in abnormal segments (p<0.05).
**Conclusion:** There were significant differences in segmental ADC values between normal and abnormal bowel segments. The ADC values are statistically highly significantly lower in abnormal bowel segments.

### *SS 6.4*

### Expert consensus on acquisition and reporting of intestinal US activity in Crohn's disease: a prospective inter-rater agreement study

R. Wilkens[1], K. Nylund[2], F. Petersen[3], F. De Voogd[4], C. Maaser[3], T. Kucharzik[3], G. Maconi[5], M. Allocca[5], C. Lu[6], C. Palmela[7], R. Vaughan[8], B. Christensen[8], K. Novak[6]; [1]Hvidovre/DK, [2]Bergen/NO, [3]Luneberg/DE, [4]Amsterdam/NL, [5]Milan/IT, [6]Calgary, AB/CA, [7]Loures/PT, [8]Parkville, VIC/AU

**Purpose:** Intestinal US (IUS) is a promising cross-sectional imaging modality used to assess transmural disease and complications in Crohn's disease (CD). Standard measurements, reproducibility and nomenclature have not yet been clearly established. The aim of this study was to evaluate inter-rater agreement for important parameters by experts through Delphi consensus.
**Material and methods:** IUS parameters demonstrating inflammatory activity were selected by a blinded Delphi IUS experts consensus panel to establish relative contribution to inflammatory activity in CD. Phase 1: blind review by 8 readers of 20 de-identified CD cases. Cases with poor agreement were reviewed to clarify discrepancy and improve agreement. Phase 2: an additional 30 de-identified CD cases blindly were reviewed by 12 independent expert readers. Inter-rater agreement was evaluated for all 4 key parameters.
**Results:** The Delphi process reduced 12 activity parameters to 4 key contributors including BWT, color Doppler signal (CDI), inflammatory fat and bowel wall echo stratification. BWT was comprised of 2 measurements in cross section and 2 in longitudinal orientation. Interobserver agreement was almost perfect for BWT: ICC=0.91 (95%CI 0.83-0.96), p=0.001, moderate agreement for CDI, κ=0.60 (95%CI 0.48-0.72), p=0.001. Agreement for inflammatory fat was also moderate with κ=0.50 (95%CI 0.33-0.66), p=0.001, while stratification was fair κ=0.39 (95%CI 0.26-0.53), p=0.001.
**Conclusion:** This expert consensus-based IUS activity score clearly establishes the reproducibility of this standardized approach to measure inflammatory activity in CD. Using our method, BWT is highly reproducible with CDI and inflammatory fat demonstrating moderate reproducibility. This score may provide the foundation for the future incorporation of IUS in research studies and clinical trials.

### SS 6.5

### Feasibility and reliability of GI US in pregnant inflammatory bowel disease patients

F. De Voogd[1], H. Joshi[2], G.R.A.M. D'Haens[1], K. Gecse[1], E. van Wassenaer[1]; [1]Amsterdam/NL, [2]Mumbai/IN

**Purpose:** Disease activity during pregnancy in women with inflammatory bowel disease (IBD) is associated with miscarriage and low birth weight. GIUS has a high potential for monitoring disease activity. The aim of this prospective study is to determine the feasibility and the reliability of GIUS in pregnant IBD patients.
**Material and methods:** Patients were included to the study when visiting the IBD pregnancy clinic. At each trimester, clinical and biochemical disease activity was evaluated and GIUS was performed. Feasibility was assessed by the ability to visualize each bowel segment (terminal ileum (TI), ascending, transverse, descending and sigmoid colon). Reliability was evaluated using fecal calprotectin (FCP) as the gold standard. Bowel wall thickness (BWT) of >3 mm in the colon and >2mm in the TI was considered as active inflammation on US.
**Results:** Thirty-two IBD patients were studied. Eleven of 32 patients had clinically active disease at least at one time point during the pregnancy. Visibility of TI and sigmoid declined from 85% and 95% in the first trimester to 22% and 45% in the third trimester, respectively. FCP levels were higher in patients with active disease on GIUS (mean 1095.5 ± 1453.8 mg/g vs 265.25 ± 649.8 mg/g, p<0.0001). When active, disease was defined as a FCP ≥ 250 mg/g, GIUS could distinguish active from non-active disease in the first, second and third trimesters with a sensitivity of 80%, 75% and 75% and specificity of 85%, 86% and 100%, respectively.
**Conclusion:** GIUS is feasible and reliable to assess disease activity throughout pregnancy in IBD.

### SS 6.6

### Early detection of mucinous adenocarcinoma within fistulating peri-anal Crohn's disease

A. Corr, D. Burling, P. Lung, L. Reza, A. Hart, P. Tozer, I. Jenkins; London/UK

**Purpose:** Development of peri-anal fistula mucinous adenocarcinoma (MA) is a presumed rare but recognised event in patients with fistulating peri-anal Crohn's disease. The true incidence is unknown with a lack of robust registry data. Fistula mucinous cancers may evade early diagnosis, often necessitating morbid exenterative surgery. This study aimed to improve early detection of these cancers.
**Material and methods:** We performed a retrospective analysis of 8 cases of biopsy-proven MA that had developed within Crohn-related anal fistula. MRI studies pre-dating the diagnosis of malignancy were reviewed to establish whether there are early predictive features of MA. Demographic, disease and surgical history were collected.
**Results:** The median time to histological diagnosis of MA from the time of diagnosis of Crohn's disease was 426 (96-480) months and from diagnosis of fistulating disease 66 (24-156) months. On review of MRI, characteristic "budding" appearance within the T2 high-signal "sepsis"/fistula tract is noted in all of our MA fistula cancers. This finding pre-dates the histological diagnosis of MA by a median of 42 months (12-156). Metastatic disease was not detected in any of the 8 cases. Potentially curative pelvic exenteration was possible in 7 patients.
**Conclusion:** There is an opportunity for early detection of the development of MA in anal fistula by recognition of "budding" within the tract. Earlier diagnosis may lessen the magnitude of subsequent radical surgery following prolonged growth of these lesions. Long-standing Crohn's anal fistula should be surveyed with MRI and any finding of "budding' within the tract on T2 sequences should prompt urgent examination under anaesthetic and biopsy.

### SS 6.7

### Inter-observer agreement of an expert panel for GIUS in ulcerative colitis

F. De Voogd[1], R. Wilkens[2], K. Gecse[1], M. Allocca[3], K. Novak[4], C. Lu[4], G.R.A.M. D'Haens[1], C. Maaser[5]; [1]Amsterdam/NL, [2]Hvidovre/DK, [3]Milan/IT, [4]Calgary, AB/CA, [5]Luneberg/DE

**Purpose:** GIUS is increasingly performed in inflammatory bowel disease to assess disease activity and treatment response. It correlates well with endoscopy and other cross-sectional imaging modalities. However, the reliability of GIUS has poorly been investigated for ulcerative colitis (UC). Therefore, we conducted a study to assess inter-observer agreement in UC.
**Material and methods:** Thirty patients with UC were included. Cine-loops were recorded for the sigmoid in a longitudinal and cross-sectional axis. Cine-loops were scored by five independent raters blinded for clinical disease activity. The cine-loops were scored for bowel wall thickness (BWT), Doppler activity (0=no signal, 1=small spots limited to the wall, 2=long stretches within the wall, 3=long stretches reaching into the mesenterium), inflammatory fat, bowel wall stratification, loss of haustration and lymph nodes (present or absent). Intraclass correlation coefficient, Fleiss' kappa and weighted Cohen's kappa were used for statistical analysis.
**Results:** Inter-observer agreement was good for bowel wall thickness (ICC: 0.7, 95% CI: 0.51-0.83) and moderate for Doppler signal (k=0.57, 95% CI: 0.37-0.77) and inflammatory fat (k=0.42, 95% CI: 0.29-0.58). When Doppler signal was interpreted as absent (0) or present (1-3), the observed agreement was almost perfect (k=0.81, 95% CI: 0.69-0.92). Agreement was fair for the presence of lymph nodes (k=0.35, 95% CI:0.20-0.49) and loss of stratification (k=0.22 95% CI: 0.09-0.35) and slight for loss of haustrations (k=0.15, 95% CI: 0.00-0.29).
**Conclusion:** GIUS is a reliable imaging modality with good-to-moderate inter-observer agreement for BWT, vascularization and fatty wrapping in UC. These ultrasonographic parameters are important features to distinguish active from quiescent disease.

### SS 6.8

### Assessment of small bowel motility in chronic intestinal pseudo-obstruction using caloric stimulation and cine-MRI

K. Van Rijn, G. Bouma, K. Horsthuis, J. Tielbeek, A. Smout, A. Bredenoord, J. Stoker, C. de Jonge; Amsterdam/NL

**Purpose:** Chronic intestinal pseudo-obstruction (CIPO) is a severe digestive disorder, characterized by failure of intestinal motility. MRI has emerged as a non-invasive method for evaluating bowel motility. This study aimed to gain insight into fasted and fed small bowel motility in CIPO patients.
**Material and methods:** Eight CIPO patients underwent a cine-MRI protocol comprising of fasting state scans and subsequently postprandial scans after orally ingesting a small-volume, high-caloric-density, test meal (Nutridrink, 300 kcal). Small bowel motility was visually scored by an experienced abdominal radiologist as well as quantified by a validated post-processing technique (GIQuant, Motilent, UK) with a new edge-detection technique to account for dilated bowel, resulting in a motility score (arbitrary units (AU)). Motility scores were compared with healthy volunteers that previously underwent a similar cine-MRI protocol.
**Results:** Visually, motility was increased in four out of eight CIPO patients, six showed distended bowels filled with air and/or intestinal content. Motility quantitation demonstrated a median fasted small bowel motility of 0.21 AU (IQR 0.15-0.30) and directly after intake of the meal 0.23 AU (IQR 0.15-0.27). In healthy volunteers, corresponding fasted and fed motility were 0.18 AU (IQR 0.14-0.24) and 0.25 AU (IQR 0.20-0.29), respectively.
**Conclusion:** Surprisingly, we found hyperactive small bowel motility in half of the CIPO patients, suggestive of uncoordinated intestinal motility. Quantitation showed a wide variation in motility patterns, with both higher and lower motility than in healthy subjects, and an absence of postprandial activation. Dynamic MRI helps to gain insight into this complex disease and can potentially impact treatment decisions in the future.

### SS 6.9

### MR fistulography with percutaneous instillation of US gel versus conventional MR protocol: technique, advantages and role in the reduction of relapse rates

G. Fontanella[1], B. Brogna[1], S. Borrelli[2], M. Mancinelli[1], S. De Lucia[1], A. Festa[1], C.A.T. Manganiello[1]; [1]Benevento/IT, [2]Avellino/IT

**Purpose:** The aim of this study is to compare our gel-enhanced MR fistulography (MRF), with our conventional MR protocol, underlying its superiority in fistular detection and its role in the reduction of relapse rates in our institution.
**Material and methods:** We selected, from September 2018 to September 2019, 65 pre-surgery patients with patent fistular orifices, excluding those with Crohn's disease and scanned them both with our conventional MR and gel-enhanced T2w MRF protocol (injecting 3-10 cc of US gel per orifice). Conventional and gel-enhanced series were randomized and reported by three in-blind experienced radiologists. Results were then compared with the surgical reports, our gold standard.
**Results:** MRF detected 87 (95.6%) out of the 91 tracts reported at surgery, whereas our basal protocol detected just 78 (85.7%). Considering the definition of fistular grade, MRF and surgery were substantially comparable. MRF and basal MR were comparable in sensibility, specificity and accuracy of the definition of internal openings, with rates ranging from 90.2% to 98.1%. Relapse rate in Sept. '18-'19 was 3.07%, substantially consistent with that of the same period a year before (2.89%) and significantly lower than relapse rates of similar periods (22.43%, Sept. '16-'17), before the introduction of MRF in our clinical practice (Sept. 2017).
**Conclusion:** MRF has shown to be superior to conventional MR protocols in sensitivity and specificity of fistular tract detection. Its introduction in our institution in Sept. 2017 prompted a drop in fistular relapses (-19.54%) and kept the relapse rate steadily at ca. 3% ever since.

### SS 6.10

### Hounsfield unit-negative bowel filling agent in oncologic patients

F.-T. Fork, I. Adnerhill, P. Leander, L. Casal-Dujat, G. Stathis; Malmö/SE

**Purpose:** To improve the detection of diseases arising from the intestinal mucosa, a Hounsfield unit (HU)-negative drinkable contrast was invented, and successfully tested in 25 healthy volunteers. After securing necessary allowances, a slightly modified HU-negative agent, Lu44, could be tested in severely ill oncologic patients without known bowel involvement.
**Material and methods:** Oncologic patients sent for abdominal CT were randomized so that 20 were administered equal volume of either Omnipaque 10%, or polyethylene glycol, and 40 Lu44. Examinations were anonymized. Small bowel luminal HU in 6-mm ROIs was independently read by two experts ignorant of patient's medical history. Patients' acceptance, adverse effects, extension and distension of filling agents were recorded.
**Results:** A half-time interim check was performed. In comparison, Lu44 revealed significant superiority in bowel wall-to-lumen contrast, high patients' acceptance, and few adverse effects. Consequently, the series was terminated after 45 patients. Mean difference between bowel wall and lumen was 86HU for Omnipaque, 63HU for polyethyleneglycol, and 484HU for Lu44. No significant differences in extension and distension were noticed. Rank test showed no notable difference between the 2 investigators' assessments. Image reading did not negatively influence.
**Conclusion:** Lu44 offers CT images with augmented bowel wall-to-lumen contrast, a prerequisite for improved diagnostics like T1-sequence images of MRI. Filling properties were not influenced by ongoing cancer treatments. Absence of side effects and high acceptance is important for these patients with severe symptoms. An ongoing series is testing Lu44 in patients with Crohn's disease.

**9:00 - 10:30** **Room E 105 / 106**

*Scientific Session SS 7*

### Intervention in hepatobiliary and pancreatic diseases: new insights

### SS 7.1

### Stereotactic image-guided microwave ablation for malignant liver tumours: a multivariable accuracy and efficacy analysis

M. Maurer, A. Lachenmayer, V. Banz, S. Weber, J. Heverhagen, P. Tinguely; Bern/CH

**Purpose:** Therapeutic success of microwave ablation for liver lesions depends on precise placement of ablation probes and complete tumor destruction. We investigated factors influencing targeting accuracy and efficacy of percutaneous stereotactic image-guided microwave ablation (SMWA) for malignant liver tumors.
**Material and methods:** All malignant liver tumors treated with SMWA over 3 years were analyzed in this observational study. A computed tomography-based navigation system was used for ablation probe trajectory planning, stereotactic probe positioning and validation of probe positions and ablation zones. Factors potentially influencing target positioning errors (TPE) of ablation probes, and local tumor progression (LTP) within 6 months, were analyzed using a multivariable regression model including challenging lesion locations (liver segments I, VII and VIII; subphrenic location).
**Results:** 301 lesions (174 HCC, 87 CRLM, 17 NET, 23 other) were targeted in 191 interventions in 153 patients. Median TPE per ablation probe was 2.9 millimeters (0.2 - 14.1 mm) (n=384). Factors significantly influencing TPE were cirrhosis (R 0.668, CI 0.218 - 1.119) and targeting trajectory length in centimeters (R 0.205, CI 0.118 - 0.291). Overall technique efficacy was 96% and LTP within 6 months 22%. Factors significantly influencing early LTP were lesion size >30 mm (OR 5.221, CI 2.435 - 11.192) and TPE >5 mm (OR 2.480, CI 1.064 – 5.784). Challenging intrahepatic lesion locations had no significant influence on TPE or early LTP.
**Conclusion:** SMWA allows precise and effective treatment of malignant liver tumors, with targeting accuracy independently predicting efficacy, while unaffected by challenging lesion locations. This might broaden treatment eligibility for otherwise difficult-to-target liver tumors.

### SS 7.2

### Resection plus post-operative adjuvant transcatheter arterial chemoembolization compared with resection alone for HCC: a systematic review and meta-analysis

Y.R. Huo, M.V. Chan, C. Chan; Sydney, NSW/AU

**Purpose:** Multiple studies have demonstrated resection plus adjuvant transcatheter arterial chemoembolization (aTACE) has demonstrated improved survival outcomes compared to resection alone for patients with HCC. This systematic review and meta-analysis aims to quantify this survival and disease-free survival (DFS) benefit.
**Material and methods:** A search of five databases was performed from inception to August 20, 2019. Survival and DFS were extracted and pooled using RevMan.
**Results:** A total of 26 (6 RCTs) involving 7817 patients were included. Patients receiving resection plus aTACE showed significantly better 1-year survival (OR, 2.53 [95% CI, 1.70-3.76, p<0.001) and 1-year DFS (OR, 1.91 [95% CI, 1.60-2.28, p<0.001) compared to resection alone. The survival benefit remained significant for both 2-, 3- and 4-year survival and DFS (Table 1). Subgroup analysis showed significant survival benefit with aTACE following resection in microvascular invasion (MVI)-positive HCC, portal venous thrombus (PVT) positive, PVT negative, no satellite nodules, resection margin 1cm or more, and a resection margin <1cm (Table 2). No mortalities were reported with adjuvant TACE.
**Conclusion:** This meta-analysis demonstrates adjuvant post-operative TACE to the remnant liver via the hepatic artery with lower chemotherapy doses than standard TACE following resection is a safe intervention and can improve the overall and disease-free survival, particularly for patients with microvascular invasion-positive HCC. The current evidence weakly supports improvement outcomes of adjuvant TACE for patients with and without PVTT, with and without a resection margin <1cm, and patients with satellite nodules. Future randomised controlled trials are warranted to confirm these findings.

### SS 7.3

### Comparison of diffusion-weighted MRI and 68Ga-DOTATATE PET/CT to assess treatment response of hepatic metastases of primary neuroendocrine tumors undergoing selective internal radiotherapy with 90Yttrium-microspheres

M.K. Ingenerf, N. Fink, I. Harun, J. Ricke, C. Schmid-Tannwald; Munich/DE

**Purpose:** To compare SUV of 68Ga-DOTATATE PET/CT and ADC values of diffusion-weighted MRI (DWI) in assessing tumor response in patients with liver metastases of primary neuroendocrine tumors (NET) following radioembolization.
**Material and methods:** 30 patients with 80 hepatic lesions of primary NET who underwent 68Ga-DOTATATE PET/CT and abdominal MRI with DWI before and after selective internal radiotherapy (SIRT) were included. Tumor size, intralesional ADCmin and ADCmean, ADCmean of normal liver parenchyma, intralesional SUVmax and SUVmean, tumor to spleen ratio (T/S ratio) and tumor to liver ratio (T/L ratio) were measured. Treatment response was evaluated with respect to Response Evaluation Criteria in Solid Tumors v1.1 (RECIST) on follow-up examination.
**Results:** 67/80 metastases were classified as stable disease (SD) and 13/80 metastases as partial response (PR). Intralesional ADCmin and ADCmean values increased significantly ($p < 0.006$) after SIRT in the group of PR and SD with a significant higher increase of ADCmin values in the PR group (54.1 ± 14.6 % vs. 24 ± 4.9 %, $p = 0.02$). Currently used SUV measurements showed significant decrease in the PR group (including SUV max, SUVmean, T/S ratio and T/L ratio), while in the SD group, only SUVmax, SUVmean and T/S ratio (max/max) decreased significantly. Using ROC curves, SUVmean was found to be the best metric (AUC 0.75); however, similar results were found for ADCmin (AUC 0.7).
**Conclusion:** DW-MRI appears to perform similar to 68Ga-DOTATATE PET/CT for quantitative response assessment in patients with liver metastases of NET who undergo SIRT and may represent a valuable functional marker to guide further treatment decisions in these patients.

### SS 7.4

### Comparison of pain score according to infusion of artificial ascites in radiofrequency ablation of subcapsular HCC

S.J. Park, D.H. Lee; Seoul/KR

**Purpose:** To determine whether there is a difference in the pain score after artificial ascites infusion when performing radiofrequency ablation (RFA) for subcapsular HCC.
**Material and methods:** From 2011 to 2016, 123 patients with single subcapsular HCC who underwent RFA were retrospectively included. Patients were divided into two groups according to the artificial ascites infusion. After RFA, the medical records were used to analyze the pain score during the 24-hour period and to see the dose and number of used opioid analgesics. We also conducted a subgroup analysis of the group closely located to the peritoneum or other solid organs. The CT or MRI taken were analyzed to see if local tumor recurrence occurred. Mann–Whitney U test and Kaplan–Meier method were used to find out the difference.
**Results:** There were 76 patients (61.8%, 76/123) with artificial ascites infusion. Pain score using numeric rating scale (NRS) score was significantly lower in artificial ascites infusion group ($p=0.048$). Dose or number of opioid use, complication, duration of hospital stay and local tumor progression were not significantly different. No peritoneal seeding was observed. For subgroup analysis, NRS score was significantly lower in artificial ascites infusion group ($p=0.004$). Although dose or number of opioid use was not significantly different ($p=0.282$, $p=0.205$, respectively), there was a tendency to use less in artificial ascites infusion group (average 1.63mg vs 2.5mg, 0.39 vs 0.5, respectively).
**Conclusion:** Artificial ascites infusion was an effective and safe way to reduce the pain in patients when performing RFA for subcapsular HCC.

### SS 7.5

### US-guided percutaneous radiofrequency ablation of locally advanced pancreatic adenocarcinoma: a 5-year high-volume centre experience

A. Giaretta, A. Beleù, R. De Robertis, A. Drudi, A. Lucarelli, M. D'Onofrio; Verona/IT

**Purpose:** The aim of the study was to investigate the safety and the effectiveness of percutaneous radiofrequency ablation (RFA) in locally advanced pancreatic cancer (LAPC) of the pancreatic body, assessing overall survival of patients and evaluating the effects of the procedure in the clinical and radiological follow-up.
**Material and methods:** Patients with unresectable LAPC after the failure of at least six months of chemoradiotherapy have been retrospectively included. Percutaneous RFA was performed after a preliminary US feasibility evaluation. Contrast-enhanced CT and CA 19.9 sampling were performed before, 24-hours and 30-days after the procedure, to evaluate the effects of the ablation. Patients were followed up after discharge considering the two main endpoints: procedure-related complications and death.
**Results:** 35 patients have been included, five of them have been later excluded. All patients underwent RFA reporting no procedure-related complications. Mean size of tumors was 49 mm before treatment. The mean dimension of the ablated necrotic zone was 32 mm, with a mean extension of 65% compared to the whole tumor size. Tumor density was statistically reduced one day after the procedure ($p<0.001$). Mean CA 19.9 levels before, 24-hours and 30-days after the procedure were 285.8 U/mL, 635.2 U/mL, and 336.0 U/mL respectively, with a decrease or stability at 30-day evaluation in 80% of cases. Mean survival was 310 (65-718) days.
**Conclusion:** Percutaneous RFA of LAPC is a feasible technique in patients who cannot undergo surgery, with great debulking effects and a very low complication rate.

### SS 7.6

### Monitoring treatment response after CT-guided high-dose-rate brachytherapy in patients with cholangiocarcinoma using apparent diffusion coefficients

N. Fink, M.K. Ingenerf, S. Goller, J. Ricke, C. Schmid-Tannwald; Munich/DE

**Purpose:** The aim of this study was to evaluate the ability of apparent diffusion coefficient (ADC) measurements to predict treatment response after CT-guided high-dose-rate (HDR) brachytherapy in patients with cholangiocarcinoma.
**Material and methods:** This retrospective pilot study included 14 patients with 20 hepatic lesions of cholangiocarcinoma who were treated by HDR brachytherapy. Pre- and postinterventional MRI including diffusion-weighted imaging (b-values= 50, 400 and 800 $s/mm^2$, mean time before and after brachytherapy: 14 days and 99 days, respectively) were performed. Tumor size, tumor enhancement, minimum, maximum and mean ADC values (ADC min, max, mean) of the lesions as well as ADC mean of normal liver parenchyma were analyzed and compared before and after brachytherapy. Treatment response was evaluated based on modified Response Evaluation Criteria in Solid Tumors (mRECIST).
**Results:** According to mRECIST, 14 (70%) lesions were characterized as responder and 6 (30%) as non-responder at the time of the first MRI after brachytherapy. ADC values of responding lesions increased significantly after brachytherapy (ADC max $p<0.001$; ADC mean $p<0.001$; ADC min $p<0.05$), whereas non-responding lesions showed no significant change. Furthermore, the ADC max values after brachytherapy were significantly higher in lesions with response than in lesions with non-response ($p<0.05$).
**Conclusion:** In patients with cholangiocarcinoma, HDR brachytherapy enables local tumor control. By measuring ADC values, particularly ADC max and mean values, therapeutic response can be monitored. This may be a useful adjunct to morphologic changes such as size and enhancement.

### SS 7.7

### Sarcopenia as a predictor for 6-month mortality after transjugular intrahepatic portosystemic shunt creation in patients with refractory ascites

L. Maruzzelli[1], R. Miraglia[1], I. Petridis[1], R. Volpes[1], A. Berzigotti[2], J. Bosch[2], F. Tuzzolino[1], A. Luca[1]; [1]Palermo/IT, [2]Bern/CH

**Purpose:** Our study is aimed at evaluating whether CT-based assessment of skeletal muscle mass is associated with mortality after transjugular intrahepatic portosystemic shunt (TIPS) creation in patients with cirrhosis and refractory ascites.

**Material and methods:** We retrospectively reviewed the clinical information and pre-TIPS CT images of patients with cirrhosis and refractory ascites who underwent TIPS, using ePTFE-covered stent, at a single center in the period between 1/2015 and 11/2018. Evaluation of skeletal muscle mass was made by measuring the psoas muscle (PMA) and total abdominal muscles areas (TAMA), in a semi-automated way, using a specific software at the level of the third lumbar vertebra.

**Results:** 90 patients (mean age 61 ± 9 years) were included. Mean pre-TIPS MELD score was 12±4. The average time between CT and TIPS was 56 days. 16 patients (17%) died within 6 months of TIPS creation. Univariate analysis demonstrated that PMA, TAMA, hemoglobin level, white blood cell count, serum albumin, serum bilirubin, portal pressure gradient (PPG) and MELD score pre-TIPS were strongly associated with 6-month mortality after TIPS. PMA had an area under the ROC curve of 0.702 for 6 months mortality and a PMA cutoff of 16 $mm^2$ (sarcopenia) was correlated with mortality. At multivariate Cox regression analysis sarcopenia, leucopenia, MELD score and PPG were independent predictors of post-TIPS 6-month mortality.

**Conclusion:** Sarcopenia, assessed by PMA on standard CT images, should be considered a risk factor for 6-month mortality in patients with cirrhosis who undergo TIPS placement for refractory ascites.

### SS 7.8

### Transjugular intrahepatic portosystemic shunt creation using the new Gore Viatorr controlled expansion endoprosthesis in cirrhotic patients with refractory ascites: a single-centre experience

L. Maruzzelli[1], C. Cannataci[2], S. Caruso[1], G. Mamone[1], A. Di Piazza[1], M. Milazzo[1], I. Petridis[1], R. Volpes[1], R. Miraglia[1]; [1]Palermo/IT, [2]Msida/MT

**Purpose:** To retrospectively evaluate clinical outcomes after transjugular intrahepatic portosystemic shunt (TIPS) creation using Viatorr controlled expansion ePTFE-covered stent (VCX) in cirrhotic patients with refractory ascites (RA).

**Material and methods:** Between July 2016 and May 2019, 85 cirrhotic patients with RA (66/19 M/F; 61± 8.5 years) received TIPS using VCX.

**Results:** Mean pre-TIPS MELD score was 13.9 ± 3.5. Before TIPS, 18 patients (21%) had a history of portosystemic encephalopathy (PSE). Sixteen patients (19%) had severe portal vein thrombosis (PVT). After TIPS, the portosystemic pressure gradient (PSG) decreased from 15.6 ± 4.5 to 6.5 ± 2.6 mm Hg (p<0.001). In 3 patients (3.5%) with severe PVT, VCX was directly dilated to 10 mm diameter reaching the hemodynamic target (PSG < 12 mmHg and/or PSG reduction ≥ 40%). In 82 patients, VCX was initially dilated to 8 mm achieving hemodynamic success in 77; in the 5 remaining patients (6%) not reaching the hemodynamic target, VCX was further dilated to 10 mm with a final PSG < 12 mmHg. Mean follow-up was 10.5 ± 8.3 months (0.2-30). Ascites was controlled without any further need for paracentesis in 53 patients (62.4%); long-term (>3 months) need of paracentesis was observed in 15 patients (17.6%). Nineteen patients (22.4%) developed grade > I PSE; two patients (2.4%) underwent stent reduction for persistent PSE. Eight patients (9.4%) underwent TIPS revision. The overall mortality was 25.9% (n = 22). Thirteen patients (15.3%) underwent liver transplantation.

**Conclusion:** In cirrhotic patients with RA, VCX can help to optimize the hemodynamic target during TIPS creation, leading to effective ascites control with acceptable complication rates.

### SS 7.9

### Stereotactic microwave ablation of malignant liver lesions using MRI/CT fusion for targeting of “invisible” lesions

M. Maurer, M. Cathomas, C. Kim-Fuchs, N. Mertineit, J. Heverhagen, A. Lachenmayer; Bern/CH

**Purpose:** In thermal ablation for treating primary or metastatic liver neoplasms, advanced stereotactic navigation technology not only improves precise tumor targeting and antenna guidance but also allows approaching lesions that are not detectable on CT planning scans by fusion with a prior MRI. The purpose of our study was to assess the technical feasibility of MRI/CT fusion for lesions invisible on CT planning scans and its outcome in patients treated with stereotactic microwave ablation (SMWA).

**Material and methods:** Patients who underwent SMWA between January 2015 and December 2018 were retrospectively analyzed. All liver lesions for which MRI/CT fusion was performed due to invisibility on pre-interventional CT planning scans were included and reassessed. The endpoint was successful ablation of the lesion at first follow-up imaging.

**Results:** During the study period, 236 patients underwent 312 SMWAs with ablation of 496 lesions. Twenty-four lesions in 15 patients (mean age 62 years; range 43 - 80 years) were included. Following MRI/CT image fusion, all 24 lesions were sufficiently visible to perform SMWA. The first follow-up imaging showed complete ablation of 22 lesions. Two initially incompletely ablated lesions were HCCs, and were successfully re-ablated afterwards.

**Conclusion:** SMWA with MRI/CT image fusion is an efficient and safe treatment option for patients with liver lesions not detectable on contrast-enhanced CT planning scans. Using MRI/CT image fusion may allow more patients with malignant liver lesions to benefit from locally ablative therapies even if their lesions are not visible on CT planning examinations.

### SS 7.10

### US fusion imaging in the percutaneous ablation of hepatic malignancies: impact on clinical practice

A. Beleù, A. Drudi, A. Giaretta, G. Rizzo, A. Lucarelli, C. Longo, M. D’Onofrio; Verona/IT

**Purpose:** To investigate the potential and limits of US fusion imaging (US-FI) in the percutaneous ablation of hepatic malignancies, assessing when this technology is essential, calculating its impact on the timing of the procedure.

**Material and methods:** 16 consecutive patients were prospectively enrolled and a total of 34 lesions (28 hepatocellular carcinomas, 6 metastases) were treated by percutaneous radiofrequency ablation or ethanol injection with US-FI guidance. Main clinical, laboratory and CT/MR imaging parameters were evaluated before and after the procedure. At the end of every single ablation, a score survey (from 1 to 10) was submitted to the radiologist. Timings of the procedure were collected.

**Results:** 30% of the lesions were local recurrence of a previous ablation; in these cases, US-FI was judged to be more useful than in primary treated lesions (p=0.021). Complete ablation was achieved in 88% of cases. US-FI was more useful when the lesion was not conspicuous in B-mode (p<0.001). US-FI significantly changed the perception of lesion size in 70% of cases. The overall confidence of the operator was highly increased by US-FI in 68% of the procedures. Mean duration of the entire procedure was 17m and 50s. In 71% of cases, the radiologist had the perception of having spared time using US-FI, especially in local recurrence treatment (p=0.024).

**Conclusion:** US-FI increases the conspicuity of target lesions and the confidence of the operator. US-FI is particularly useful for the targeting of less conspicuous lesions and for the treatment of recurrences of previously ablated lesions. In these cases, US-FI is a time-sparing method.

**11:00 - 12:30** **Room Emerald**

*Scientific Session SS 8*

### Rectal cancer imaging to improve patient care

### *SS 8.1*

### Impact of new nodal staging guidelines in rectal cancer

A. Jankovic[1], N.W. Schurink[1], J. van Griethuysen[1], R. Geenen[2], V. Cappendijk[3], M. Maas[1], M.J. Lahaye[1], R.G.H. Beets-Tan[1], D.M.J. Lambregts[1]; [1]Amsterdam/NL, [2]Alkmaar/NL, [3]'s-Hertogenbosch/NL

**Purpose:** The ESGAR-guidelines on MRI of rectal cancer advise the use of specific nodal staging criteria to determine the N-stage for primary rectal cancer staging, mainly aiming to avoid overstaging. These criteria were adapted from the Dutch national colorectal cancer guidelines that were introduced in 2014. The aim was to explore the clinical impact of the implementation of these Dutch guidelines on primary nodal staging outcomes in the Netherlands.
**Material and methods:** The primary staging MRIs of n=96 rectal cancer patients (from 3 Dutch centers) were analyzed: 48 patients from <2014 (pre-guideline) and 48 from >2014 (post-guideline). A dedicated reader determined the N-stage (N0/N1/N2) for each case, blinded to the original reports, using the criteria from the Dutch/ESGAR guidelines, where nodes are considered positive when ≥9mm, 5-8 mm with two morphologically suspicious criteria (round/irregular/heterogeneous), or <5 mm with 3 suspicious criteria. Results were compared to the N-stage in the original clinical reports, derived from the hospitals' patient databases.
**Results:** Before 2014, the N-stage determined using the Dutch/ESGAR criteria was concordant with the original reports in 79% of the cases, the remaining 21% were downstaged when the guideline was applied. After 2014, scorings were concordant in 96% of the cases, 2% were upstaged, and 2% were downstaged. The difference in concordant/discrepant findings before and after 2014 was significant (P=0.014 Chi-square).
**Conclusion:** The results of this exploratory study suggest that the introduction of more strict nodal staging criteria has led to a significant reduction in overstaging of the nodal status in rectal cancer in the Netherlands.

### *SS 8.2*

### Value of high-resolution diffusion-weighted imaging for the evaluation of extramural tumour invasion in primary rectal cancer

S.H. Kim, E.J. Park, S.J. Jo; Busan/KR

**Purpose:** To determine the added value of diffusion-weighted imaging (DWI) to T2-weighted imaging (T2WI) for the evaluation of extramural tumor invasion (EMTI) in patients with primary rectal cancer.
**Material and methods:** Seventy-eight patients (51 men, 27 women; mean age, 67 years; range, 49-87 years) who had undergone 3.0-T MRI including high-resolution DWI (2x2x2 mm3 isovoxel, b=0, 1000 s/mm2) and subsequent surgery were included in this retrospective study. To evaluate the added value of DWI, two blinded radiologists independently read the T2WI first and 4 weeks later the combined (T2WI+ DWI) set. They recorded their confidence level using a 5-point scale (1, definitely negative; 2, probably negative; 3, equivocal; 4, probably positive; and 5, definitely EMTI positive). The diagnostic accuracy for each reviewer was calculated by ROC curve analysis. The added value of DWI to T2WI for EMTI evaluation was determined by pair-wise comparison of the ROC curves. The histopathological results served as the reference standard for EMTI.
**Results:** The study population consisted of T1 (n=9), T2 (n=25), T3 (n=38) and T4 (n=6) patients and sub-divided into positive EMTI (n=44) and negative EMTI (n=34) groups. The AUC was not significantly increased after adding DWI to T2WI (for reader 1, 0.868 (95% CI, 0.772 – 0.934) to 0.856 (95% CI, 0.758 – 0.925), P=0.5618; for reader 2, 0.848 (95% CI, 0.748 – 0.919) to 0.865 (95% CI, 0.769 – 0.932), P=0.4539).
**Conclusion:** Adding DWI to T2WI showed no additional diagnostic value for the evaluation of EMTI in patients with primary rectal cancer.

### *SS 8.3*

### The role of read-out segmented echo-planar imaging-based diffusion-weighted imaging in evaluating tumour response of locally advanced rectal cancer after neoadjuvant chemoradiotherapy

L. Yang, B. Wu; Chengdu/CN

**Purpose:** To evaluate the role of diffusion weighted imaging (DWI) based on read-out segmented echo-planar imaging (rs-EPI) in assessing tumor response after neoadjuvant chemoradiotherapy (CRT) in locally advanced rectal cancer (LARC).
**Material and methods:** 63 LARC who received neoadjuvant CRT and surgery were enrolled retrospectively. They all underwent pre- and post-CRT MR examinations, including DWI using rs-EPI. According to pathological results, patients were grouped as pathological complete responder (pCR, n=16) and non-pCR (n=47). Visual assessment of residual tumor and whole-tumor histogram analysis of pre- and post-CRT apparent diffusion coefficient (ADC) map was performed by two radiologists, and tumor volume on ADC map was also recorded.
**Results:** Overall interobserver agreement was good for histogram analysis (ICC=0.543-0.999). Tumor volume reduction rate on ADC map showed no significant difference between the two groups (P=0.468). Post-CRT mean, quantile values, and their percentage changes were higher in the pCR group (all P<0.001). Post-CRT mean value had good diagnostic power in selecting pCR (AUCs=0.855), with a cut-off value of 1.345 x 10-3mm2/s, yielding a sensitivity of 83% and specificity of 81.3%. Post-CRT quantile 95% value had the highest AUCs (AUCs=0.868) among quantile values and a higher specificity (87.5% vs. 81.3%) than mean value with comparable overall diagnostic performance (P=0.563). Visual assessment showed a sensitivity of 85.1% and specificity of 68.8% in selecting pCR.
**Conclusion:** Quantitative ADC value of rs-EPI DWI could reliably evaluate tumor response in patients with LARC. Post-CRT 95% quantile ADC value could help mean value to more accurately identify pCR.

### *SS 8.4*

### MRI predictors of positive radial margin in low rectal cancer patients treated with neo-adjuvant treatment for unsafe surgical planes

A. Chandramohan, A. Patra, D. Masih, R. Mittal; Vellore/IN

**Purpose:** To assess MRI predictors of positive circumferential resection margin (CRM) in patients with low rectal cancer (LRC) treated with neo-adjuvant chemo-radiotherapy (NACRT).
**Material and methods:** This is a retrospective study of patients with LRC (distal margin < 5 cm from anal verge) who underwent NACRT for unsafe surgical planes on staging MRI and subsequently underwent surgery. T2 high-resolution images (T2-HR) with diffusion-weighted imaging (DWI) on staging and re-staging MRI were reviewed. Imaging features were compared with pathological CRM (pCRM) to determine features that best predicted the surgical radial margin.
**Results:** 166 patients (M:F = 102:64) with age of 46 +/- 13.6 years, range of 21–86 years, were included. 17.5% had positive pCRM. There was a significant association between pCRM and extramural spread of tumor and CRM on staging MRI and similarly between pCRM and ymr-CRM, unsafe surgical planes, puborectalis infiltration, ymr-T and N stages, MR-tumor regression grade (MR-TRG) on restaging MRI, p<0.05. 34.1% and 31% of patients with positive pCRM had extramural vascular invasion and tumor deposits, respectively, on re-staging MRI with trend towards significant association (p=0.06, 0.07). With the use of both T2-HR and DWI for re-staging, CRM was correctly identified as involved by tumor or EMVI in 82.6%. MR-identified fibrosis had tumor at pathology in 10.2%. Re-staging MRI had 100% sensitivity, 76% specificity, 100% NPV, and 81% accuracy for predicting pCRM.
**Conclusion:** Re-staging MRI has high diagnostic accuracy for predicting CRM in high-risk low rectal cancer patients treated with neo-adjuvant chemo-radiotherapy.

### SS 8.5

### Reliability in villous tumours staging between preoperative MRI and histopathological examination

C. Vogrig, J. Leclerc, V. Haghnejad, X. Orry, T. Remen, A. Germain, V. Laurent; Vandoeuvre/FR

**Purpose:** This study aimed to assess the reliability of rectal villous tumors staging between rectal MRI and histological examination used as the gold standard and to investigate causes for discrepancies.
**Material and methods:** The rectal 1.5T MR scans of 40 patients followed for a histologically proven rectal villous adenoma were retrospectively included. Two independent experienced radiologists staged each tumor according to the TNM classification and described the occurrence of retraction of the rectal wall or spiculations within the associated mesorectum. A third radiologist collected tumor's morphological characteristics.
**Results:** The mean volume of tumors with spiculations and retraction was significantly greater ($p<0.05$) compared to tumors without these characteristics. Spiculations and retraction of the rectal wall were observed regardless of the definitive histological stage and did not represent a malignancy criterion. A weak inter-observer reliability (Gwet's AC2: 0.31 [0.04–0.57]) in T-staging was observed between the two readers. Reader 1 showed a high reliability (Gwet's AC2: 0.90 [0.81–0.99]) in T-staging between histopathological examination and preoperative MRI. In the opposite, reader 2 showed a weak reliability (Gwet's AC2: 0.31 [0.03–0.58]) in T-staging. He overstaged all tumors (100%) with spiculations ($p <0.05$).
**Conclusion:** MRI understaged rectal villous adenoma and was unable to detect degenerative criteria, along with slight interobserver agreement. The typical pejorative signs of rectal tumors, such as retractions and spiculations, occurred in all stages and were responsible for misstaging in most cases, in particular, bulky tumors.

### SS 8.6

### The value of intravoxel incoherent motion imaging and diffusion kurtosis imaging in assessing tumour regression and T stage after neoadjuvant chemoradiotherapy in locally advanced rectal cancer

L. Yang, B. Wu; Chengdu/CN

**Purpose:** To explore the effectiveness of intravoxel incoherent motion (IVIM) imaging and diffusion kurtosis imaging (DKI) in assessing tumor regression grade (TRG) and pathological T stage (ypT) of locally advanced rectal cancer (LARC) after neoadjuvant chemoradiotherapy (nCRT).
**Material and methods:** 111 LARC who received nCRT and subsequent surgery were enrolled prospectively. They all underwent pre- and post-CRT rectal MRI with IVIM and DKI sequences. According to the pathological TRG and ypT stage, patients were grouped as complete responders (CR, TRG0) and non-complete responders (non-CR, TRG1-3), low-T stage (ypT0-2) and high-T stage (ypT3-4) groups. ROIs were manually drawn on three largest slices of tumor on b value of 800s/mm2 imaging. Apparent diffusion coefficient (ADC), IVIM parameters (D,D* and f) and DKI parameters (MD and MK) were calculated.
**Results:** The CR group had higher post-CRT MD ($P=0.005$) and ADC value ($P=0.018$), and higher absolute change of f ($P=0.017$), MD ($P=0.018$), and ADC value ($P=0.018$) after nCRT than non-CR group. Post-CRT MD (AUC=0.718) had a similar performance in selecting CR to ADC (AUC=0.698, $P=0.930$). The absolute change of f (AUC=0.747, specificity=85.7%) and MD (AUC=0.767, specificity=85.7%) had higher specificities than ADC (AUC=0.743, specificity=71.4%), with comparable overall diagnostic power. After nCRT, the low-T stage group had higher post-CRT D ($P=0.01$), f ($P=0.042$), MD ($P=0.003$), and ADC value ($P<0.001$) than high-T stage group. These four parameters yielded respective AUCs of 0.693, 0.641, 0.723, and 0.723 in assessing ypT stage after nCRT in LARC.
**Conclusion:** IVIM and DKI could help to assess tumor regression grade and T stage after nCRT in patients with LARC.

### SS 8.7

### Predicting response to therapy in locally advanced rectal cancer: radiomic analysis from MRI

M. Micilotta[1], G. Cappello[2], V. Giannini[1], A. Defeudis[1], S. Mazzetti[2], C. Costanza[1], M. Gatti[1], S. Cirillo[1], R. Faletti[1], D. Regge[1]; [1]Turin/IT, [2]Candiolo/IT

**Purpose:** To predict the response to neoadjuvant therapy in patients with locally advanced rectal cancer (LARC) using texture features of MRI staging examination, to customize therapeutic management.
**Material and methods:** The dataset is composed of 96 patients acquired in three different institutions with different MR scanners. All colorectal cancers (CRCs) were manually segmented on the T2w images. CRCs were classified as non-responder (R-) if their TRG score was >=3 and responder (R+) otherwise. Seventy-seven radiomics features were extracted from voxels belonging to a 3D mask manually segmented in the T2w and apparent diffusion coefficient (ADC) and fed into a stepwise logistic regression model. Patients of two institutions were randomly assigned to the training set (TrS) (70%) and testing set (TeS) (30%), while patients of the third institutions were used as the validation set (VdS).
**Results:** The dataset included pR+ 44 and pR- 52 tumors. 10 features were kept by the logistic regression classifier that obtained an accuracy on the TrS of 0.8, with sensitivity and specificity of 0.83 and 0.86, respectively. Accuracy on the TeS was 0.76, with sensitivity, specificity, NPV and PPV of 0.86, 0.67, 0.86, 0.67, respectively. VaS reported accuracy of 0.68, sensitivity of 0.58, specificity of 0.78, NPV of 0.64 and PPV of 0.73.
**Conclusion:** This study demonstrates the possibility to recognize, analyzing MRI staging examination, patients that will not respond to conventional neoadjuvant therapies, to customize treatment and avoid the use of ineffective therapies.

### SS 8.8

### MR tumour regression grade with diffusion-weighted imaging in locally advanced rectal cancer compared with standard MR tumour regression grade and pathological tumour regression grade: feasibility and prognostic role

M. Franchi, B. Desana, M. Micilotta, L. Vercelli, A. Balbo Mussetto, A. Fornari, C.V. Lario, A. Macera, M. Petracchini, D. Molinar, S. Osano, S. Cirillo; Turin/IT

**Purpose:** Evaluate the accuracy and the prognostic role of MR tumor regression grade (TRG) after neoadjuvant radiochemotherapy (RCT) in locally advanced rectal cancers (LARC) using T2 plus diffusion-weighted imaging (DWI) sequences (mrTRG_DWI) comparing to standard T2-only mrTRG and pathological TRG (pTRG).
**Material and methods:** 36 patients were enrolled in this retrospective study, all underwent a pre- and post-CRT conventional MRI study followed by surgical resection with a median 36-month follow-up. A 5-point mrTRG and mrTRG_DWI were assessed by two expert radiologists and finally compared with pTRG.
**Results:** Concordance between mrTRG and pTRG was low (k=0.45); concordance between mrTRG_DWI and pTRG was good (k=0.73), with an AUROC of 0.85 and 0.92, respectively. The mrTRG_DWI had a sensibility and specificity, respectively, of 90% and 75%, with a correct diagnosis in 83% patients. Kaplan-Meier and log-rank tests showed a reduction of disease free survival for any increase of the TRG_DWI ($p=0.004$).
**Conclusion:** mrTRG_DWI is a valid system to assess chemoradiotherapy response and a prognostic factor. Our results suggest to implementing mrTRG_DWI in larger and prospective correlative studies as a tool to select patients for whom deferred surgery may be appropriate.

### SS 8.9

### Complete response evaluation of locally advanced rectal cancer to neoadjuvant chemoradiotherapy using textural features on MRI

S. Azamat, Ş. Karaman, C.B. Kulle, I.F. Azamat, M. Keskin, R.N. Dagoglu Sakin, M.G. Dagoglu Kartal, B. Bakir; Istanbul/TR

**Purpose:** To evaluate the diagnostic performance of T2-weighted imaging and apparent diffusion coefficient (ADC) textural features for the prediction of pathologic complete response after neoadjuvant chemoradiotherapy (CRT) in locally advanced rectal cancer (LARC).
**Material and methods:** Between April 2015 and April 2019, 44 patients (32 men, 12 women; median age: 57 years), who underwent MRI with ADC before and after CRT, were enrolled in this retrospective study. Histopathological findings were the standard of reference. Textural features of LARC on T2-weighted and ADC images were extracted and compared between pathologic complete responders versus partial responders using Student's t test or Mann-Whitney U test. Receiver operating characteristic (ROC) curves were calculated to detect the diagnostic performance of ADC.
**Results:** 22 of the 44 (50%) patients were complete responders, and 50% (22/44) were partial responders regarding pathological results after neoadjuvant CRT. Quantitive ADC changes correlated with treatment response and a cut off 1310 x 10-6 mm2/sn predicted pathologic total response with a sensitivity of 72% and a specificity of 77%. CRT induced a significant decrease in T2-weighted signal intensity (T2wSI) in all patients after neoadjuvant CRT (p=0.001 for complete responders vs p=0.003 for partial responders). Also, skewness changes of T2wSI between pre- and post-CRT showed a significant difference in complete responders in contrast to partial responders (p=0.002 for complete responders vs p=0.611 for partial responders).
**Conclusion:** In patients with LARC, changes in the skewness of the T2wSI and quantitive changes in ADC are useful for predicting a favorable response to neoadjuvant CRT.

### SS 8.10

### Selection of patients for organ preservation after chemoradiotherapy: MRI identifies poor responders who can go straight for surgery

H.E. Haak[1], M. Maas[1], M.J. Lahaye[1], T. Boellaard[1], A. Delli Pizzi[2], C. Mihl[3], D. van der Zee[4], C. Fabris[5], M. van der Sande[1], J. Melenhorst[3], R.G.H. Beets-Tan[1], G. Beets[1], D.M.J. Lambregts[1]; [1]Amsterdam/NL, [2]Chieti/IT, [3]Maastricht/NL, [4]Uden/NL, [5]Verona/IT

**Purpose:** To evaluate whether MRI is accurate to identify poor responders after chemoradiotherapy who will need straight surgery and to evaluate whether results are reproducible amongst radiologists with different levels of expertise.
**Material and methods:** Seven independent readers with different expertise retrospectively evaluated the restaging MRIs (T2W+DWI) of 62 patients to categorize them as [1] poor responders—highly suspicious of tumour, [2] intermediate responders—tumour most likely, and [3] good—potential (near) complete responders. Reference standard was histopathology after surgery (or long-term follow-up in case of a watch-and-wait program).
**Results:** Fourteen patients were complete responders, 48 had residual tumour. Median percentage of patients categorized as "poor", "intermediate" and "good" responders by the 7 readers was 21% (range 11-37%), 50% (range 23-58%) and 29% (range 23-42%). The vast majority of the poor responders had histopathologically confirmed residual tumour (of which 73% ypT3-4) with a low rate (0-5%) of "missed complete responders". Of the 14 confirmed complete responders, a median percentage of 71% were categorized in the MR-good response and 29% in the MR-intermediate response group.
**Conclusion:** Radiologists of varying experience levels should be able to use MRI to identify the subgroup of ±20% of poor responding patients who will unavoidably require surgical resection after CRT. This may facilitate a more selective use of endoscopy, particularly in general settings or in centers with limited access to endoscopy.

**11:00 - 12:30** **Room E 105 / 106**

*Scientific Session SS 9*

### Imaging the acute abdomen

### SS 9.2

### Non-occlusive mesenteric ischemia: CT features of reperfusion and their prognostic value

G. Bagnacci, S. Guerrini, V. Pelini, G. Zipoli, L. Volterrani, M.A. Mazzei; Siena/IT

**Purpose:** Non-occlusive mesenteric ischemia (NOMI) is a often a misdiagnosed and a dangerous condition. To our knowledge, an objective evaluation of CT parameters that could correlate with the outcome of patient suffering from NOMI is still missing. The study's aim was to investigate quantitative CT features of reperfusion in NOMI and their prognostic value.
**Material and methods:** Contrast-enhanced CT examination of 71 patients with a confirmed diagnosis of NOMI (37 with highly suggestive clinical and laboratory features and 34 biopsy proven) were retrospectively reviewed analyzing vessels, mesentery, bowel and peritoneal cavity CT features. Diameter of superior mesenteric artery (SMA), celiac trunk (CeT), inferior vena cava (IVC), superior mesenteric vein (SMV) and differences in CT Hounsfield units (Delta HU) of the bowel wall before and after intravenous contrast media (ICM) administration were correlated to the outcome using Mann-Whitney test. Receiver operating characteristic (ROC) curves were elaborated and combined using predicted probability.
**Results:** A significant correlation between diameters of SMA, CeT, IVC and SMV and outcome were found (Mann-Whitney p=0.015, p=0.13, p=0.01, and p=0.02 respecrively). Delta HU after ICM administration results also correlated (p=0,005). Area under the curve (AUC) of single variables results were as follows: CeT= 0.672; SMA= 0.667; SMV= 0.717; IVC= 0.728; Delta HU= 0.692. The combined AUC was 0.825.
**Conclusion:** SMA, IVC, SMV, CeT diameters and Delta HU results correlated with patients suffering from NOMI. A combination of those values allows an accurate prediction of NOMI.

### SS 9.3

### Porto-mesenteric venous gas: is surgery still mandatory?

L. Besnier, C. Lesage, J.F. Hamel, A. Hamy, C. Aubé, A. Paisant, A. Venara; Angers/FR

**Purpose:** Porto-mesenteric venous gas (PMVG) is associated with mesenteric ischemia in 40-60% of cases with a high mortality rate requiring emergency surgery. Studies reported a decrease in the mortality rate with the identification on CT of non-ischemic underlying causes and unnecessary surgery. The aim of this study was to identify prognostic factors of mortality in patients with PMVG and to assess factors leading to unnecessary surgery in these patients.
**Material and methods:** 88 patients with PMVG diagnosed by CT from January 2008 to December 2017 were retrospectively included. Clinical, biological and radiological findings were collected and their association with mortality and unnecessary surgery was assessed.
**Results:** The overall mortality rate was 45.5%. Etiologies of PMVG were bowel ischemia (42%), gastrointestinal (GIT) obstruction or dilatation (16%), post-abdominal surgery (14.7%), GIT infection or sepsis (8%), trauma (6.8%), unspecified (6.8%) and malignancy (5.7%). Mortality was significantly associated with bowel ischemia (p<0.001) and with radiological findings of bowel ischemia (lack of wall enhancement (p=0.005), pneumatosis intestinalis (p=0.001), arterial occlusion (p=0.021) and solid organ infarction (p=0.046)). In multivariate analysis, the absence of acidosis was the only sign associated with a reduction of mortality [OR=0.09; 95% CI: 0.02-0.52]. No independent factor was significantly associated with unnecessary surgery.
**Conclusion:** PMVG remains serious, especially when revealing bowel ischemia and when associated with acidosis. Unfortunately, no specific sign is associated with unnecessary surgery that can orientate the physician toward the decision or not of surgical management.

### SS 9.4

### Endovascular revascularisation of acute arterial mesenteric ischemia: 3-year experience report from a dedicated intestinal stroke centre unit

M. Najdawi[1], A. Nuzzo[1], L. Raynaud[1], L. Maggiori[1], A. Sibert[1], Y. Castier[2], O. Corcos[1], V. Vilgrain[1], M. Ronot[1]; [1]Clichy/FR, [2]Paris/FR

**Purpose:** To report the 3-year experience of endovascular revascularization of arterial acute mesenteric ischemia (AMI) from a dedicated intestinal stroke center unit (ISCU).
**Material and methods:** All patients admitted to the ISCU between January 2016 and January 2019 for arterial AMI who underwent endovascular recanalization were retrospectively analyzed. Patients' demographics, clinical and laboratory characteristics at presentation, together with CT scans were reviewed. The type (thrombolysis, angioplasty, stenting, vasodilators) and the outcome of endovascular procedures (technical success or failure, complications) were noted. Care pathways were described focusing on post-procedural treatments (endovascular or open revascularization, intestinal resection). The 3-month mortality rate was noted.
**Results:** 59 patients (35 men [59%], mean 69±12 yr) were included. Endovascular revascularization was technically successful in 55/59 (93%) patients, and 15 (25%) patients had post-procedural complications. Following this procedure, 33 patients (56%) were alive and recurrence free without further treatment, while 7 (12%), 4 (7%) and 6 (10%) patients underwent 2nd line endovascular revascularization, open revascularization, and resection, respectively. Seven patients (12%) underwent more than one 2nd line treatment. Overall, 48 patients (81%) were alive and recurrence free (mortality rate: 19%).
**Conclusion:** Endovascular revascularization is highly feasible for the treatment of arterial AMI, and is associated with an acceptable rate of complications. These results can only be interpreted in the context of an integrated multidisciplinary and dedicated management of patients that limits the need for bowel resection, and leads to good survival rates.

### SS 9.5

### CT angiography for overt GI bleeding: extravasation volume correlates with prognosis and bleeding rate

J.R. Tse, J. Shen, D. Fleischmann, A. Kamaya; Stanford, CA/US

**Purpose:** To measure the extravasation volume of first-pass CTA in patients with overt GI bleeding (GIB) and to correlate volume with clinical outcomes.
**Material and methods:** In this retrospective, IRB-approved and HIPAA-compliant study, we reviewed all patients from 1/2014 to 7/2019 who presented with overt GIB and had active extravasation on CTA. Extravasation volume (EV) was calculated using 3D segmentation software. Imaging data were correlated with clinical data, including if further workup was pursued, active bleeding was identified, and blood transfusions were required following CTA.
**Results:** 50 consecutive patients met inclusion criteria, corresponding to 6 (12%) upper, 18 (36%) small bowel, and 26 (52%) lower GIB. 42 patients underwent further workup with catheter angiography, endoscopy, or surgery, with a median elapsed time of 9 hours between CTA and further workup. Of 42, 16 (38%) had active bleeding intra-operatively and all underwent intervention with successful hemostasis. Arterial extravasation volume was 2.72±2.97 mL for those with active bleeding and 0.76±1.01 mL for those without (p=0.0009). Using a threshold volume of 0.80 mL, the odds ratio of requiring massive transfusion was 7.0 (95% confidence interval 1.9-26; p=0.004), identifying active bleeding was 11.8 (2.6-54; p=0.002), and therapeutic yield was 6.8 (1.5-30; p=0.01). Using a previously described mathematical model, bleeding rate can be estimated from extravasation volume and attenuation, and the lower limit of detected bleeding rate was calculated to be < 0.1 mL/minute.
**Conclusion:** Larger extravasation volumes may be used to triage patients who require additional management and improve therapeutic yield. Current CTAs can detect bleeding rates lower than 0.1 mL/minute.

### SS 9.6

### Severe ischemic colitis: CT findings and histopathological correlation

V. Fayeulle[1], M. Haberlay[1], O. Ernst[2]; [1]Valenciennes/FR, [2]Lille/FR

**Purpose:** To identify CT findings in patients with acute ischemic colitis (AIC) surgically treated and to correlate them with the severity of the colonic necrosis in histopathology.
**Material and methods:** CT data of 50 consecutive patients with AIC surgically treated were independently reviewed retrospectively by 3 radiologists. Histopathological slides were reviewed by an experimented pathologist to assess patients with no or limited necrosis to the mucosa or submucosa and those with extended necrosis and to compare CT features in each group. Interobserver agreement (K) was assessed for each CT finding.
**Results:** Twenty-five patients (50%) were classified as group limited necrosis and 25 patients (50%) as group extended necrosis. The most frequent CT signs observed were the abnormal wall enhancement (76%) and colon wall thickening or thinning (82 and 70%, respectively). Parietal pneumatosis (PP) was found in 8 patients (32%) of the group extended necrosis against 2 (8%) in the group limited necrosis (p: 0.024) with a sensibility for detection of extended necrosis of 0.32 (95% confidence interval [95% CI], 0.15 - 0.53) and a specificity of 0.92 (95% CI, 0.74 – 0.99).Portomesenteric venous gas (PVMG) was mostly seen in the group with limited necrosis (5/6 patients, K: 1). Three cases of increased unenhanced colonic wall attenuation and 3 of intra-luminal blood clot were observed.
**Conclusion:** PP is a significant sign of severe colonic necrosis in the clinical context of AIC. Isolated PVMG should be considered carefully because it could be associated with less extensive necrosis.

### SS 9.7

### CT diagnosis of closed loop bowel obstruction mechanism is not sufficient to indicate emergent surgery

C. Rondenet[1], I. Millet[2], A. Dallongeville[1], L. Corno[1], W. Khaled[1], I. Boulay Coletta[1], P. Taourel[2], M. Zins[1]; [1]Paris/FR, [2]Montpellier/FR

**Purpose:** To identify CT findings associated with successful conservative treatment of closed loop small bowel obstruction (CL-SBO) due to adhesions or internal herniation.
**Material and methods:** The local institutional review board approved this study while waiving informed consent. Clinical and CT data were collected retrospectively for 96 consecutive patients with a CT diagnosis of CL-SBO due to adhesions or internal herniation established by experienced radiologists who had no role in patient management. Mechanical obstruction with at least two transition zones on the bowel at a single site defined CL-SBO. Two radiologists blinded to patient data independently performed a retrospective review of the CT scans. The patient groups with successful versus failed initial conservative therapy were compared. Univariate and multivariate analyses were performed to look for CT findings associated with successful conservative therapy. Interobserver agreement was assessed for each CT finding.
**Results:** Of the 96 patients, 34 (35%) underwent immediate surgery and 62 (65%) received first-line conservative treatment, which succeeded in 19 (31%) and failed in 43 (69%). The distance between the transition zones was the only independent predictor of successful conservative therapy (odds ratio, 4.6 when ≥8 mm; 95% confidence interval [95%CI], 1.2-18.3). A distance ≥ 8 mm had 84% (95%CI, 60-97) sensitivity and 46% (95% CI, 31-62) specificity for successful conservative treatment. The correlation coefficient for the distance between transition zones was fair (r=0.46).
**Conclusion:** CL-SBO can resolve without surgery. When there is no CT sign of ischemia, the distance between the transition zones should be assessed.

### SS 9.8

### Artificial intelligence for small bowel obstruction on plain X-ray: a deep learning ensemble method

D.H. Kim[1], H. Wit[2], M. Thurston[1], M.G. Long[2], G.F. Maskell[1], M.J. Strugnell[1], D. Shetty[1], I.M. Smith[1], N.P. Hollings[1]; [1]Truro/UK, [2]Plymouth/UK

**Purpose:** Small bowel obstruction is a life-threatening surgical emergency. Abdominal X-ray is often the first imaging test but there is usually a delay between acquisition and interpretation by a radiologist. This can lead to delay in diagnosis and management which is known to lead to poor patient outcomes including higher mortality. The aim of this study was to investigate the potential for faster automated identification of small bowel obstruction on abdominal X-rays using deep learning.
**Material and methods:** A total of 990 abdominal X-rays were retrieved and labelled as either normal or demonstrating small bowel obstruction using a combination of the radiology report, surgical operation notes, subsequent CT scan reports, and additional radiology review. These were used to train, validate and test 5 convolutional neural networks. These were combined into a predictive ensemble model.
**Results:** The ensemble model achieved an area under the receiver operator curve (AUC) of 0.961, sensitivity of 91% and specificity of 93%. The model was able to make a prediction in approximately 146 milliseconds per image.
**Conclusion:** This study demonstrates that deep learning can be used with high accuracy and speed for the automated identification of small bowel obstruction on abdominal X-ray. This could be used to flag urgent findings to the radiologist or surgeon resulting in faster diagnosis and improved patient outcomes.

### SS 9.9

### Role of fast abdominal MRI protocol in emergency settings and new scanning techniques

E. Siopis, F. Monetti, P.E. Orlandi, R. De Domenico, A. Perrone, M. Imbriani; Bologna/IT

**Purpose:** Abdominal emergencies sometimes are a diagnostic challenge. CT is the imaging modality of choice in patients with uncertain US scan. Public awareness of ionizing radiation exposure has increased in recent years. The purpose of this study is to assess the role of a fast MRI protocol (about 10 minutes) using new technologies, as the first-line imaging method in abdominal emergencies and to show the radiological findings that allow the characterization of various pathologies seen in the emergency setting, particularly among a select patient population.
**Material and methods:** 6008 patients (mean age 26.8±13.2y) with acute abdomen (2068 pregnant patients) underwent fast protocol MRI (Philips 1.5T with multichannel coil: T2w FS cor, T2w ax, T1wFS GRE ax and steady-state free precession GRE cor, DWI) as first-line imaging modality, at our imaging department between March 2014 and November 2019. The final diagnosis was established by surgical findings in 4088 cases. The remaining patients underwent follow-up.
**Results:** MRI allowed correct therapeutic management in 6008 cases (68 acute pancreatitis, 46 choledocholithiasis with or without cholecystitis, 3002 acute appendicitis, 2880 bowel obstruction, 2 ectopic pregnancy, 10 deep endometriosis) in young, pregnant patients, and patients with Crohn's disease.
**Conclusion:** Our study demonstrated that fast MRI can play a role in providing accurate diagnosis in emergency settings, in selected patients with acute abdomen and pelvic pain, particularly among select patient populations, specifically paediatric and pregnant patients as well as patients with Crohn's disease, who are at risk of receiving repeated imaging examinations and large cumulative radiation doses over their lifetimes.

**14:30 - 16:00** **Room E 102**

*Scientific Session SS 10*

### Imaging of HCC: new trends

### SS 10.1

### Liver imaging reporting and data system category 5 HCC: preoperative MRI for the prediction of early recurrence after hepatectomy

H. Wei, H.-Y. Jiang, T. Zheng, Z. Zhang, B. Song; Chengdu/CN

**Purpose:** To identify preoperative gadoxetic acid-enhanced-MRI biomarkers for the prediction of early recurrence (2 years) after curative resection for liver imaging reporting and data system (LI-RADS) category 5 (LR-5) HCC.
**Material and methods:** Between July 2015 and July 2018, this retrospective study evaluated consecutive treatment-naïve high-risk patients who underwent gadoxetic acid-enhanced MR examination within 1 month before surgical resection for HCC. Tumor recurrence was determined by clinical and imaging follow-up. All MR images were reviewed by three independent radiologists regarding the LI-RADS v2018 imaging features, LI-RADS categories and several non-LI-RADS imaging features of all high-risk lesions. The Cox proportional hazards model was used to analyze the predictors for early recurrence after hepatectomy.
**Results:** A total of 125 patients (99 men and 26 women, mean age: 49.84 ± 11.83 years) with 164 LR-5 HCCs (mean size: 6.0 ± 3.4 cm) were included; 62 (49.6%) patients experienced tumor early recurrence. The multivariate Cox hazards model revealed that corona enhancement (hazard ratio (HR), 2.180; p = 0.008), peritumoral hypointensity on hepatobiliary phase (HBP) (HR, 2.904; p = 0.000), multifocality (HR, 2.179; p = 0.003) and serum alpha-fetoprotein (AFP) level > 400 ng/mL (HR, 2.182; p = 0.003) were independent risk factors for early recurrence. Further, AUC of the obtained predictive model was approximately 0.80 for the combination of three imaging biomarkers with or without the integration of AFP.
**Conclusion:** In high-risk patients with LR-5 HCC, preoperative HBP peritumoral hypointensity, corona enhancement, multifocality and serum AFP can be used to effectively predict early recurrence after curative hepatectomy.

### SS 10.2

### Hepatobiliary phase-abbreviated MRI for HCC detection: a five-year, single-centre, experience

A. Pecorelli, P.M. Dautt-Medina, A. Mamidipalli, J.Y. An, N. Layyous, R.L. Brunisng, M.T. Booker, C. Sirlin, K.J. Fowler; San Diego, CA/US

**Purpose:** To describe the five-year, single-center, clinical experience of abbreviated MRI (AMRI) protocol for HCC surveillance.
**Material and methods:** The radiology reports for all AMRIs performed through May 2019, at a single referral center, were retrospectively reviewed. Each AMRI examination was scored as negative (no or only definitely benign observations), subthreshold (observations <10 mm) or positive (observations ≥ 10 mm, not definitely benign). Each patient was categorized as surveillance-negative, surveillance-subthreshold or surveillance-positive according to their highest AMRI score they had during the inclusion period. Blinded to those categories, each patient was classified as positive or negative for HCC using a composite reference standard that integrated all available clinical, imaging and pathology data.
**Results:** 1160 AMRIs were performed in 561 patients. 185 (33%) patients were lost to follow-up. Of the remaining 376 patients, 295 (79%) were surveillance-negative, 23 (6%) surveillance-subthreshold, and 58 (15%) surveillance-positive. Based on the composite reference standard, 292/295 (99%) surveillance-negative and 20/23 (87%) surveillance-subthreshold patients were HCC-negative (true negatives); 29/58 (50%) of surveillance-positive patients were HCC-positive (true positives). Sensitivity, specificity, and accuracy of surveillance AMRI were 86%, 91% and 90%, respectively.
**Conclusion:** Hepatobiliary-phase AMRI surveillance is feasible clinically in patients in whom US surveillance is compromised. Our preliminary single-center experience suggests it provides higher sensitivity (86% vs 78%) and specificity (98% vs 89%) [4] with a lower loss to follow-up (33% vs 38%) [5] than historically reported US surveillance.

### SS 10.3

### Combined hepatocellular cholangiocarcinoma: changes in 2019 World Health Organization histological classification and their impacts on liver imaging reporting and data system classification

S.K. Jeon, J.M. Lee, S.S. Lee, S.H. Choi, H.-J. Kang, B.Y. Hur; Seoul/KR

**Purpose:** To investigate the impact of recently updated 2019 5th World Health Organization (WHO) classification of primary liver cancers on the performance of liver imaging reporting and data system (LI-RADS) v2018 for combined hepatocellular cholangiocarcinoma (cHCC-CCA) in differentiating from HCC at gadoxetic acid-enhanced MRI (Gd-EOB-MRI).
**Material and methods:** In this retrospective study, 209 patients with surgically proven cHCC-CCAs (according to the 4th WHO classification) who had undergone preoperative Gd-EOB-MRI between 2009 and 2018 were included. A retrospective review of all surgical specimens and histopathologic rediagnosis was performed by a pathologist according to the recently updated 5th WHO classification. LI-RADS category was assigned for each lesion by five abdominal radiologists in consensus. LI-RADS categories were compared across cHCC-CCA, HCC, and intrahepatic cholangiocarcinoma (IHCC) using a $\chi^2$ test.
**Results:** 15.3% (32/209) of previously diagnosed cHCC-CCAs were rediagnosed as HCCs according to the 5th WHO classification, and those HCCs were categorized as LR-5, LR-M, and LR-TIV in 53.1% (17/32), 37.5% (12/32), and 9.4% (3/32), respectively. 6.2% (13/209) of tumors were rediagnosed as IHCCs, and categorized as LR-M and LR-4 in 92.3% (12/13) and 7.7% (1/13). No IHCCs were categorized as LR-5. The remained 78.5% (164/209) was rediagnosed as cHCC-CCAs, and categorized as LR-5/4, LR-M, and LR-TIV in 47% (77/164), 43.9% (72/164), and 9.1% (15/164), respectively (P=0.005).
**Conclusion:** Although approximately 20% of cHCC-CCAs were rediagnosed as HCCs or IHCCs according to the recently updated 5th WHO classification of primary liver cancer, a substantial proportion of cHCC-CCAs can be categorized as non-LR-M, making it difficult to differentiate from HCC.

### SS 10.4

### CT-derived liver surface nodularity and sarcopenia as prognosticators in patients with resectable HCC developed on metabolic syndrome

M. Seror, R. Sartoris, F. Cauchy, M. Bouattour, P.-E. Rautou, V. Paradis, O. Soubrane, V. Vilgrain, M. Ronot; Clichy/FR

**Purpose:** To assess the prognostic value of liver surface nodularity (LSN) quantification and sarcopenia on routine preoperative CT in patients with resectable HCC developed on metabolic syndrome (MS).
**Material and methods:** Patients with MS undergoing hepatectomy for HCC between 2006 and 2018 at a single center were analyzed. LSN was assessed at the left liver-fat interface and sarcopenia was derived from the skeletal muscle surface on L3 on preoperative CT. The association between LSN and sarcopenia and major (Dindo-Clavien 3-5) postoperative complications (POC) was analyzed on multivariate analysis. The influence of LSN and sarcopenia on overall (OS) and recurrence-free survival (RFS) was assessed.
**Results:** 98 patients (81 [83%] men, mean 68±8 yr) were analyzed, including 22 (22%) being sarcopenic. Patients with major POC had significantly higher LSN (mean 2.60±0.26 vs. 2.46±0.34 without complication, p=0.049, AUROC 0.64±0.06, optimal cut-off >2.47 with 77% sensitivity and 52% specificity) and were more frequently sarcopenic (42 % vs. 13 % without complication, p=0.020). Sarcopenia was the sole factor associated with major POC on multivariate analysis (OR: 70.0 [95%CI 1.87–2626.0, p=0.022)). Combining LSN>2.47 and sarcopenia stratified patients in terms of risk of major POC from 9% (no sarcopenia and LSN<2.47) to 64% (both sarcopenia and LSN>2.47), and RFS (median 61 months (19-103) to 17 months (10-24), respectively, p=0.045), but not OS. Results remained significant in patients without advanced fibrosis.
**Conclusion:** LSN quantification and sarcopenia can be easily derived from preoperative CT. Their combination seems valuable in the anticipation of major postoperative complications and RFS stratification in patients with metabolic syndrome and resectable HCC.

### SS 10.5

### Can quantitative parameters derived from hepatobiliary phase predict tumour aggressiveness in patients with HCC?

Z. Ye, B. Song; Chengdu/CN

**Purpose:** To prospectively determine whether the quantitative imaging parameters derived from hepatobiliary phase (HBP) can be used for the preoperative prediction of HCC with highly aggressive characteristics.
**Material and methods:** 103 patients with surgically proven HCC were included from July 2015 to June 2018. Signal intensity (SI) of liver and tumor was measured by two independent reviewers and quantitative parameters including relative tumor enhancement (RTE), tumor to liver contrast ratio (TLR), tumor enhancement index (TEI) and relative enhancement ratio (RER) were calculated. The aggressive characteristics of HCC were identified using Ki-67 labeling index (LI) and patients were classified into low-aggressive (Ki-67 LI ≤ 10%) and high-aggressive (Ki-67 LI > 10%) groups. Difference of quantitative parameters between two groups were assessed, and the correlation was explored. ROC analyses were used to evaluate the predictive performance of quantitative parameters.
**Results:** RTE, TLR, TEI and RER were significantly lower in the high-aggressive group than the low-aggressive group ($p < 0.05$) and negative correlations were obtained between these quantitative parameters and Ki-67 LI (r ranges from -0.41 to -0.22, $p < 0.05$). TLR demonstrated the highest predictive performance with the AUC of 0.83 (95% CI: 0.75-0.90), sensitivity of 89.0% and specificity of 63.3%, and subsequent with RER, TEI, and RTE with AUC of 0.78 (95% CI: 0.68-0.85), 0.74 (95% CI: 0.64-0.82) and 0.68 (95% CI: 0.58-0.77), respectively. Good inter-observer and intra-observer agreements were found.
**Conclusion:** TLR showed the highest predictive performance in highly aggressive HCC. Quantitative parameters based on HBP could preoperatively predict the aggressiveness of HCC.

### SS 10.6

### Long-term evolution of LR-2, LR-3 and LR-4 observations in cirrhotic patients with hepatitis C treated with direct-acting antivirals

R. Cannella, F. Vernuccio, S. Greco, M. Midiri, G. Brancatelli; Palermo/IT

**Purpose:** To assess the long-term evolution of observations with low (LR-2), intermediate (LR-3) and high (LR-4) probability for HCC in cirrhotic patients with hepatitis C virus (HCV) treated with direct-acting antivirals (DAA).
**Material and methods:** This retrospective study assessed 2017 consecutive HCV patients treated with DAA between 2015 and 2019. Inclusion criteria were i) cirrhosis or prior history of HCC; ii) available contrast-enhanced liver imaging studies (CT or MRI), iii) multiple follow-ups before and after DAA; iv) at least one indeterminate lesion before DAA. Two radiologists reviewed imaging studies, recorded major imaging features and categorized each lesion according to LI-RADSv2018. Differences in evolution before and after DAA were evaluated using the Pearson $\chi^2$ or Fisher's exact test. Cumulative risk of LR-5 was calculated using the Kaplan-Meier method.
**Results:** Final population included 67 patients (mean age 69.5±10.8 years) with 109 observations (mean size 11.8±6.7mm), including 31 (28.4%) LR-2, 67 (61.5%) LR-3 and 11 (10.1%) LR-4 with a mean follow-up of 44±23 months. Evolution of indeterminate observations to LR-5 was more common before DAA than after DAA (before DAA: 0 (0%) LR-2, 11 (16.4%) LR-3 and 8 (72.7%) LR-4 evolved to LR-5 vs. after DAA: 1 (3.2%) LR-2, 10 (14.9%) LR-3 and 1 (9.1%) LR-4 evolved to LR-5; p<0.001). Cumulative risk for LR-5 evolution was 15.5% at 6 months, 23.8% at one year and 37.6% at two years. LI-RADS category was significantly associated with risk of progression into LR-5 (p<0.001).
**Conclusion:** The use of DAA does not increase the progression of indeterminate lesions into definitively HCC.

### SS 10.7

*Withdrawn by the authors*

### SS 10.8

### Prognostic value of liver imaging reporting and data system classification on gadoxetic acid-enhanced MRI and 18F-fluorodeoxyglucose positron emission tomography/CT in patients with primary liver carcinomas

J.H. Min[1], S.H. Kim[1], S.-Y. Choi[2], J. Lee[3]; [1]Seoul/KR, [2]Bucheon/KR, [3]Cheonju-si/KR

**Purpose:** To evaluate the postsurgical prognostic value of the liver imaging reporting and data system (LI-RADS) categories on gadoxetic acid-enhanced MRI and 18F-fluorodeoxyglucose positron emission tomography with computed tomography (FDG PET/CT) in patients with primary liver carcinomas.
**Material and methods:** Two hundreds and four patients (154 men and 50 women; mean age, 58.2 ± 9.3 years) with chronic liver disease and surgically proven single primary liver carcinoma (136 hepatocellular carcinomas, 43 intrahepatic cholangiocarcinomas, and 25 combined hepatocellular-cholangiocarcinomas) were retrospectively evaluated with gadoxetic acid-enhanced MRI and PET/CT between 2008 and 2019. Two independent reviewers assigned an LI-RADS category and measured the maximum standardized uptake value (SUVmax) for each observation. Overall survival (OS), recurrence-free survival (RFS), and their associated factors were evaluated using the Kaplan-Meier method and Cox proportional hazard model.
**Results:** In multivariable analysis, LI-RADS category (LR4 or LR 5 vs. LR-M; hazard ratio [HR], 1.8; 95% confidence interval [CI], 1.0, 3.0; P = 0.035) and SUVmax (low, < 5.8 vs. high, > 5.8; HR, 4.0; 95% CI, 2.3, 7.0; P < 0.001) were independent factors for OS. The SUVmax (HR, 2.8; 95% CI, 1.9, 4.1; P < 0.001) and CA 19-9 elevation (HR, 2.3; 95% CI, 1.4, 4.0; P = 0.002) were independent factors for RFS. Primary liver carcinomas with both LR-M category and high SUVmax (≥ 5.8) showed the worst OS and RFS.
**Conclusion:** LI-RADS category and SUVmax on PET/CT were associated with postsurgical prognosis of primary liver carcinomas, regardless of pathologic diagnosis.

### SS 10.9

### Value of washout appearance in transitional and hepatobiliary phases: diagnosis of small or atypical hepatic observations at CT

Z. Zhang, B. Song; Chengdu/CN

**Purpose:** To determine the added value of gadoxetic acid-enhanced MRI for contrast-enhanced CT (CECT)-detected small (<20 mm) or atypical hepatic observations (≥20 mm) for diagnosis of HCC.
**Material and methods:** This retrospective study included 172 patients at high risk for HCC with 214 nodules (HCCs,n=177; non-HCCs,n=37). Imaging diagnoses of CT criterion for HCC were made based on imaging features of arterial phase hyperenhancement (APHE) and portal or delayed washout at CT. And three diagnostic MRI criteria for HCC were defined according to the phases during which washout appearance was observed: 1) APHE plus washout confined to the portal venous phase(PVP), 2) APHE plus washout during the PVP or transitional phase (TP), and 3) APHE plus washout during the PVP, TP or hepatobiliary phase (HBP). The sensitivities and specificities of those criteria were compared using McNemar's test.
**Results:** Among 135 small observations measuring <20 mm, APHE plus washout extended to TP and HBP showed higher sensitivities (97.47% for both) than APHE plus washout on PVP (79.75%) and CT (46.84%; p values<0.001). The specificity of washout extended to TP was better than that with washout extended to HBP (75.00% vs. 64.6%; p=0.003). Moreover, for atypical hepatic observations at CT, APHE plus washout extended to HBP showed significantly higher sensitivity of 78.18% than that with washout on PVP and extended to TP (63.64% and 74.55%, respectively; p values<0.001), with compromising specificity of 73.91%.
**Conclusion:** Extending washout appearance to the transitional or hepatobiliary phase (instead of restricting it to the portal venous phase) provided added value to CECT for the diagnosis of HCCs in small or atypical observations with higher sensitivity.

### SS 10.10

### Direct-acting antiviral agents in hepatitis C virus patients and risk of occurrence and recurrence of HCC: is it still a clinical dilemma?

F. Vernuccio, R. Cannella, S. Greco, M. Midiri, G. Brancatelli; Palermo/IT

**Purpose:** To evaluate the clinical impact of direct-acting antivirals (DAA) therapy in patients with hepatitis C virus (HCV) in terms of HCC risk. Specifically, our objective was to evaluate intra-individual rates of occurrence and recurrence of HCC in HCV patients before and after sustained virologic response to DAA.
**Material and methods:** We retrospectively included consecutive HCV patients with sustained virologic response to DAA between 2015 and 2019 imaged with CT or MRI. We excluded patients lacking pre-DAA and post-DAA imaging follow-up. A combined reference standard (imaging and pathology) was used for HCC diagnosis. Two radiologists reviewed imaging studies, categorized each lesion and assessed treatment response according to LI-RADSv2018. Incidence rates of HCC before and after DAA therapy were calculated. Intra-individual differences in HCC occurrence and recurrence were evaluated with the McNemar test.
**Results:** Final cohort included 65 patients (mean age: 70±10 years). Overall, pre-DAA and post-DAA mean follow-ups were 47±26 months, 25±25 months and 24±15 months, respectively (p=0.472). HCC occurrence was demonstrated in a significantly larger number of patients before DAA than after DAA (n=48 (73.8%) vs. n=25 (38.5%), respectively; p=0.002). Of the 25 patients with HCC occurrence post-DAA, 18 had indeterminate lesions (LR-2, LR-3, LR4) at baseline imaging. After DAA, HCC occurred with a mean interval of 18±16 months, and 60% (n=15) of HCC occurred after one year. HCC recurrence was similar before and after DAA (p=0.092).
**Conclusion:** HCC occurrence is significantly reduced in patients with sustained virologic response to DAA; conversely, HCC recurrence is not significantly affected by DAA.

**9:00 - 10:30** **Room E 102**

*Scientific Session SS 11*

### Diffuse liver diseases: assessment of portal hypertension and liver function

### SS 11.1

### First report of the International Registry for Congenital Portosystemic Shunts

S. Franchi-Abella[1], D. Debray[2], S. Korff[3], F. Guerin[1], V. Casotti[4], M. Colledan[4], L. D'Antiga[4], J. De Ville De Goyet[5], E. Lurz[6], X. Stephenne[7], N. Rock[3], L. Hierro[8], I. Kanavaki[9], J. Lipsich[10], M. Lopez-Santamaria[8], M. Magnusson[11], Y. Mozer-Glassberg[12], M.D. Ponce[8], O. Durmaz Ugurcan[13], S. Varma[14], T.L. Pop[15], B. Wildhaber[3], E. Gonzales[1], V. Mclin[3]; [1]Le Kremlin-Bicêtre/FR, [2]Paris/FR, [3]Geneva/CH, [4]Bergamo/IT, [5]Palermo/IT, [6]Munich/DE, [7]Brussels/BE, [8]Madrid/ES, [9]Athens/GR, [10]Buenos Aires/AR, [11]Stockholm/SE, [12]Petach Tikvah/IL, [13]Istanbul/TR, [14]New Dehli/IN, [15]Cluj-Napoca/RO

**Purpose:** To describe patients followed by centres participating in the first International Registry for Congenital Portosystemic Shunts (IRCPSS).
**Material and methods:** Retrospective analysis of patients with CPSS from 15 centres. Porto-hepatic shunts were defined as intrahepatic (IH-CPSS) and others were defined as extrahepatic (EH-CPSS).
**Results:** 242 patients were analysed with 122 IH-CPSS, 120 EH-CPSS. 24% were identified pre-natally with 75% IH-CPSS. 189 (76%) patients were diagnosed post-natally at a mean age of 39.1mo (0-200) for IH-CPSS and 61.9mo (0-192) for EH-CPSS. Both forms were equally frequent when diagnosed post-natally. Among them, symptoms were equally frequent among IH-CPSS (57%) or EH-CPSS (61%) CPSS. When comparing IH-CPSS and EH-CPSS, IH-CPSS were more likely to have neonatal hypoglycemia (9.8%vs 5%) and cholestasis (22.9% vs 15%) and EH-CPSS were more likely to have cardiopulmonary (33.3% vs 10.6%), neurocognitive (31.6% vs 9%) complications and liver nodules (39% vs 18%). Patients with EH-CPSS were more likely to have several symptoms than patients with IH-CPSS. Closure: 38.5% of IH-CPSS closed spontaneously vs 3% of EH-CPSS. When persistent, IH-CPSS were mostly treated using embolization and EH-CPSS using surgery with about 40% preventive closure. 8 patients had liver transplantation.
**Conclusion:** Prenatal diagnosis shows higher prevalence of IH-CPSS. Frequent spontaneous closure of IH-CPSS explains the equal frequency of both forms on post-natally diagnosed group. CPSS should be sought in infants with hypoglycemia or cholestasis and in children and adults with liver nodules, cardiopulmonary symptoms or neurocognitive deficits. IRCPSS will be helpful for further studies.

### SS 11.2

*Withdrawn by the authors*

### SS 11.3

### Comparison between transient elastography and liver surface nodularity for the detection of clinically significant portal hypertension

A. Souhami, R. Sartoris, P.-E. Rautou, F. Cauchy, O. Soubrane, V. Vilgrain, M. Ronot; Clichy/FR

**Purpose:** To compare the performance of liver surface nodularity (LSN), liver stiffness (LS) measured by transient elastography (TE) and derive a score for the detection of clinically significant portal hypertension (CSPH) in patients with HCC developed on cirrhosis.
**Material and methods:** Patients with HCC developed on cirrhosis who underwent CT, LS and hepatic venous pressure gradient (HVPG) measurements within 30 days between 2015 and 2018 were included. Accuracy of LSN, LS, and LS-spleen-size-to-platelet ratio score (LSPS) for predicting CSPH was evaluated with the area under ROC curve (AUROC).
**Results:** A total of 140 patients underwent both tests (109 men [78%], mean 63±9 yo), including 39 (28%) with CSPH. LSN was valid in 130 patients (93%) and correlated with HVPG (r=0.68; P<.001). Patients with CSPH had a higher LSN than those without (3.1±0.4 vs. 2.5±0.3, p<0.001; AUROC: 0.87±0.31). LS measurement was valid in 132 patients (94%) and correlated with HVPG (r=0.75, P<.001; AUROC 0.87±0.04). In patients with both valid tests (n=122), there was no significant difference in terms of diagnostic performance between LSN, LS and LSPS (DeLong, P=.28 to .65). A two-step algorithm combining LSN and LSPS resulted in 108/140 patients (77%) correctly classified with 8% error.
**Conclusion:** LSN score showed similar diagnostic performance and feasibility as LS for detecting CSPH. Combination of LSN and LSPS may improve non-invasive patient classification.

### SS 11.4

### Comparison of MR elastography and US shear wave elastography in hepatic fibrosis

N. Kalra, J. Singh, A. Gulati, A. Duseja, A. Das, M.S. Sandhu; Chandigarh/IN

**Purpose:** To compare the accuracy of MR elastography (MRE) and shear wave elastography (SWE) for staging hepatic fibrosis in patients with chronic liver disease (CLD) using liver stiffness measurement (LSM).
**Material and methods:** In this prospective study, both MRE and SWE were performed in 40 patients with CLD within 2 weeks of a liver biopsy. There was a technical failure of MRE acquisition in 2 patients (5%). SWE measurement was successful in all patients.
**Results:** 6 patients (15.8%) had no fibrosis (F0), 12 patients (31.6%) had stage F1, 7 patients (18.4%) had stage F2, 7 patients (18.4%) had stage F3 and 6 patients (15.8%) had stage F4 on histopathology. A significant positive correlation was seen between LSM by MRE and fibrosis stage (ρ=0.844, p<0.001) and LSM by SWE and fibrosis stage (ρ=0.556, p<0.001). AUROC values of MRE for detection of any fibrosis (F≥1), significant fibrosis (F≥2) and severe fibrosis (F≥3) were 0.966, 0.928, and 0.902, respectively, with optimal cut-off values being 3.15 kPa, 3.30 kPa, and 3.65 kPa, respectively. AUROC values of SWE for the detection of any fibrosis (F≥1), significant fibrosis (F≥2) and severe fibrosis (F≥3) were 0.768, 0.737, and 0.809, respectively, with optimal cut-off values being 5.46 kPa, 7.50 kPa, and 7.67 kPa, respectively. Statistically significant difference was seen between the AUROC values of MRE and SWE for detecting significant fibrosis (0.928 vs 0.737, p=0.038). Sub-group analysis, based on the aetiology of CLD, showed no statistically significant difference between MRE and SWE.
**Conclusion:** MRE is better than SWE for the detection of significant fibrosis irrespective of the aetiology of CLD.

### SS 11.5

**Head-to-head comparison of liver stiffness measurement using transient elastography, 2D-shear wave elastography and MR elastography for non-invasive evaluation of clinically significant portal hypertension**

F. Andrade, M. Ronot, P. Garteiser, L. Elkrief, R. Sartoris, M. Dioguardi Burgio, M. Zappa, L. Castera, B. Van Beers, P.-E. Rautou, V. Vilgrain; Clichy/FR

**Purpose:** To compare technical success rate and diagnostic performance of transient elastography (TE), 2D-shear wave elastography (2D-SWE) and MR elastography (MRE) for the detection of clinically significant portal hypertension (CSPH) in patients with compensated cirrhosis.

**Material and methods:** A monocentric prospective study was conducted including patients with biopsy-proven cirrhosis, without history of liver decompensation, undergoing TE, 2D-SWE, MRE and hepatic venous portal gradient (HVPG) measurements within <30 days. Technical success rate was defined for TE according to European Association for the Study of the Liver (EASL) guidelines; for 2D-SWE as homogenous color with stability for ≥3 seconds in the region of interest, and for MRE as any value obtained. Ability of estimating HVPG and identifying patients with CSPH was compared between TE, 2D-SWE and MRE using area under ROC curve (AUROCs).

**Results:** We included 44 patients (32 men; median 58 yr; 26 (59%) with CSPH). Technical success rate was higher with MRE (100%), than with TE (89%; p=0.024) or 2D-SWE (93%; p=0.075). HVPG correlated with TE (r=0.692; p<0.0001), 2D-SWE (r=0.623; p<0.0001), and MRE parameters [storage modulus (Gd), r=0.522, p=0.001; loss modulus (Gl) r=0.540, p=0.0007; shear modulus (Gabs) r=0.529, p=0.0009)]. Patients with CSPH had higher liver stiffness by TE (32 vs. 17kPa; p=0.01), 2D-SWE (20 vs. 14kPa; p=0.008) or MRE (Gd 5.9 vs. 3.8kPa, p=0.01; Gl 3.2 vs.1.9kPa, p=0.005; Gabs 7.0 vs. 4.5kPa; p=0.008) than patients without. AUROCs ranged from 0.87 to 0.81 and did not significantly differ between the three methods.

**Conclusion:** In patients without any history of cirrhosis decompensation, liver TE, 2D-SWE and MRE correlated well with HVPG and had similar performance for detecting CSPH.

### SS 11.6

*Withdrawn by the authors*

### SS 11.7

**The role of gadoxetic acid-enhanced MRI in predicting post-hepatectomy liver failure after major hepatic resection for colorectal cancer metastasis after chemotherapy and diagnosing chemotherapeutic-associated liver injuries**

L. Vercelli, M. Micilotta, M. Franchi, B. Desana, A. Balbo Mussetto, A. Fornari, C.V. Lario, A. Macera, D. Molinar, M. Petracchini, S. Cirillo; Turin/IT

**Purpose:** To investigate whether preoperative gadoxetic acid-enhanced MRI predicts post-hepatectomy liver failure (PHLF) after major hepatic resection (MHR) for colorectal cancer (CRC) metastasis after chemotherapy and diagnosing chemotherapeutic-associated liver injuries.

**Material and methods:** 48 patients with CRC metastasis underwent gadoxetic acid-enhanced MRI after chemotherapy and after MHR. The signal intensity of liver parenchyma was measured using regions of interest at four segments on pre-contrast T1w imaging (SIpre) and on hepatocyte phase 20 minutes after gadoxetic acid infusion (SIhp); the mean value was calculated at each phase. The relative liver enhancement (RLE) was calculated with the formula: (SIhp - SIpre)/SIpre.

**Results:** There was a significant correlation between aspartate aminotransferase (AST) (p<0.00001), alanine aminotransferase (ALT) (p<0.00001), indocyanine green retention test (p=0.0005), number of chemotherapy cycles (p=0.001) and RLE. 17 patients (35.42%) experienced PHLF. A significant difference of RLE in the two groups (p=0.002) was found, with a cut-off value of 0.81 (sensitivity: 64.7 %, specificity: 32.2%). 2 patients (4.17%) had certain diagnosis of nonalcoholic steatohepatitis (NASH), 15 (31.25%) uncertain and 24 (50%) had no NASH. 7 patients' data were not available. No significant difference of RLE was found between groups with and without diagnosis of NASH. Sinusoidal dilatation grade 1 was present in 13 (27.08%) patients, grade 2 in 22 (45.83%) and grade 3 in 11 (22.92%). 1 patient (2.08%) had no sinusoidal dilatation. 1 patient's data were not available. No significant correlation was found between sinusoidal dilatation grade and RLE.

**Conclusion:** Preoperative gadoxetic acid-enhanced MR can predict PHLF after MHR, but cannot diagnose chemotherapeutic-associated liver injuries.

### SS 11.8

### The value of MRI-defined sarcopenia in predicting hepatic decompensation and mortality in patients with chronic liver disease

L. Beer, N. Bastati-Huber, M. Mandorfer, S. Pötter-Lang, K. Lampichler, D. Lauber, Y. Bican, B. Simbrunner, G. Semmler, M. Trauner, T. Reiberger, A. Ba-Ssalamah; Vienna/AT

**Purpose:** To explore whether sarcopenia, diagnosed by an abbreviated MRI protocol is a risk factor for hepatic decompensation and mortality in patients with chronic liver disease (CLD).

**Material and methods:** In this retrospective single-centre study, we included 265 patients (164 men, mean age 54 ±16 years) with CLD who had undergone MRI of the liver between 2010 and 2015. Transverse psoas muscle thickness (TPMT) was measured on unenhanced and contrast-enhanced T1-weighted and T2-weighted axial images. Sarcopenia was defined by height-adjusted and gender-specific cut-offs in women as TPMT <8mm/m and in men as TPMT <12mm/m, respectively. Patients were further stratified into three prognostic stages according to the absence of advanced fibrosis (FIB-4<1.45, non-advanced CLD), compensated-advanced CLD (cACLD); and decompensated-advanced CLD (dACLD).

**Results:** The inter-observer agreement for the TPMT measurements (κ=0.98; 95% confidence-interval [CI]: 0.96-0.98), as well as the intra-observer agreement between the three image sequences (κ=0.99; 95%CI: 0.99-1.00) were excellent. Sarcopenia was not predictive of further hepatic decompensation. In patients with cACLD and dACLD, sarcopenia was a risk factor for mortality (cACLD: hazard ratio (HR): 3.13, 95%CI: 1.33-7.06; dACLD: HR: 2.34, 95%CI: 1.29-4.26) on univariate analysis. After adjusting for sex, Child-Turcotte-Pugh score, serum creatinine, and sodium levels, sarcopenia (adjusted HR: 2.66, 95% CI: 1.05-6.75) remained an independent risk factor for mortality in patients with cACLD.

**Conclusion:** Sarcopenia can be easily evaluated by a short MRI exam without the need for contrast injection. Sarcopenia is a risk factor for mortality, especially in patients with cACLD.

### SS 11.9

### Diagnostic performance of contrast-enhanced CT for the diagnosis of porto-sinusoidal vascular disease: a case-control study

S. Pellegrino, A. Nivolli, S. Valainathan, M. Ronot, O. Bruno, A. Plessier, V. Paradis, P.-E. Rautou, V. Vilgrain; Clichy/FR

**Purpose:** To identify features at CT that can be used to raise suspicion of porto-sinusoidal vascular disease (PSVD) in patients with signs of portal hypertension.

**Material and methods:** This retrospective monocentric study included patients with PSVD who underwent a liver biopsy between 2011 and 2018 and performed CT within a year from biopsy. Each patient with PSVD was matched on severity of ascites with two patients with histologically proven cirrhosis. CT images were reviewed by two independent radiologists, not aware of the liver disease, and classified according to pre-specified criteria.

**Results:** 52 patients with PSVD [23 women; 60 years (43-68)] and 104 patients with cirrhosis [23 women; 62 years (52-68)] were included. Patients with PSVD had more significantly frequent abnormalities of intrahepatic and/or extrahepatic portal vein, mesenteric and/or splenic vein thrombosis, inter-hepatic vein collaterals, hepatic veins near the capsulae and had larger spleen. Patients with PSVD had less frequently an association of atrophy of segment IV with hypertrophy of segment I, a nodular liver surface and fibrous bands. Using binary logistic regression, imaging features associated with PSVD were the absence of combined atrophy of segment IV-hypertrophy of segment I, and absence of nodular liver surface (OR=4.7 (IC95% 1.8-12.3) p=0.002 for both). Sensitivity and specificity of the association of these two criteria for the diagnosis of PSVD was 67% and 84%, respectively.

**Conclusion:** In patients with signs of portal hypertension, absence of combined atrophy of segment IV with hypertrophy of segment I and absence of nodular surface of the liver should raise suspicion of PSVD.

### SS 11.10

### Chronic diffuse liver disease multiparametric US imaging: attenuation imaging and dispersion imaging role in the quantitative evaluation of steatosis and viscosity

M. Radzina, Y. Solskaya, P. Prieditis, M. Tirane, A. Lioznovs, A. Staka, S. Pavlovics; Riga/LV

**Purpose:** The aim of the study was to analyze the diagnostic value of liver US attenuation imaging (ATI) and dispersion imaging in the quantitative evaluation of steatosis and viscosity in cases of diffuse chronic diseases.

**Material and methods:** During the prospective study from April 2019 till September 2019, 47 patients with confirmed diffuse liver disease by various etiologies: virus hepatitis B (n=3) and hepatitis C (n=13), autoimmune hepatitis (n=4), steatohepatosis (n=13) and alcohol-related cirrhosis (n=5) were examined with Canon Aplio i800 ultrasound equipment, 50 healthy patients were taken as control cohort. Multiparametric evaluation by US was performed: US elastography for fibrosis, ATI for steatosis and dispersion imaging for viscosity.

**Results:** There was statistically significant difference among etiology groups and ATI (p=0.035) with predominantly high-attenuation intensity range >0.5-0.9 dB/cm/MHz and median value in steatohepatosis and relatively higher in VHC group up to 0.7. ATI median values had moderate correlation with steatosis qualitative assessment on B mode image (normal or hyperechogenic liver structure) ($r_s$=0.5; p=0.0001). There was no statistically significant difference in viscosity (dispersion) values among etiology groups. Dispersion data revealed alterations in liver tissue viscosity with moderate linear correlation to fibrosis stage ($r_p$=0.40; p=0.005) with higher value tendency towards higher fibrosis stage, but no correlation to steatosis findings (p=0.051).

**Conclusion:** Multiparametric ultrasound of chronic liver disease can provide added value with reliable quantitative measurements in steatohepatosis using ATI. Dispersion imaging has no defined thresholds for liver viscosity alterations among various etiologies, but shows correlation to fibrosis grade.

**9:00 - 10:30** **Room E 105 / 106**

*Scientific Session SS 12*

### State of the art imaging of pancreatic diseases

### SS 12.1

### Preoperative prediction of pancreatic neuroendocrine neoplasm grade using multi-phase MDCT radiological features and texture analysis

H. Yu, B. Song; Chengdu/CN

**Purpose:** To determine MDCT radiological features and texture features that can be used to predict the pathological grade of pancreatic neuroendocrine neoplasm (PNEN) pathological grade.
**Material and methods:** Patients with pathologically proven PNEN were included. Preoperative CT images in the arterial phase (AP) and the portal vein phase (PVP) were obtained. Radiological features, including diameter, margin, location, hepatic metastasis, distal main pancreatic ductal dilation, pancreatic atrophy, calcification in tumor, vascular in tumor, ratio of tumor to pancreatic parenchyma in AP and PVP, were reviewed by two radiologists, independently. 3D regions of interest (ROIs) were used to extract texture features from AP and PVP images. A chi-squared test, Student's t test, the least absolute shrinkage and selection operator (Lasso), kappa statistics, uni- and multivariate logistic regression analyses were used to analyze differences between low- and high-grade PNEN.
**Results:** 163 PNENs were included. Diameter, margin, hepatic metastasis and ratio of tumor to pancreatic parenchyma in AP were significantly different between G1 and G2/3 PNEN. In multivariate analysis, diameter (odds ratio: 1.423) and ill-defined margin (odds ratio: 3.176) were independent significant predictors for G2/3 PNEN. The areas under the receiver operating characteristic curve of the radiological feature model, AP texture model, and PVP texture model, all established by logistic regression, were 0.780, 0.774, and 0.788, respectively.
**Conclusion:** In conclusion, ill-defined margin and diameter >3cm were shown to be helpful in predicting high-grade PNEN. Texture analysis (TA) of multi-phase MDCT images can be used as a beneficial tool in the preoperative prediction of PNEN pathological grade.

### SS 12.2

### Factors influencing the detection rate of transabdominal US for incidentally detected pancreatic cysts: a prospective multicentre study

J.H. Kim, M.H. Yu, E.S. Lee, H.-J. Kang, H.J. Park, Y.S. Park; Seoul/KR

**Purpose:** To investigate the detection rate of transabdominal US (TAUS) for incidentally detected pancreatic cysts on CT or MRI as well as factors influencing detection rates.
**Material and methods:** Fifty-seven patients with incidentally detected pancreatic cysts (n = 77, 5mm to 3cm) on CT or MRI were prospectively enrolled at five institutions. TAUS was performed independently by two radiologists and assessed the detection of cysts, their location and size, and the diameter of the main pancreatic duct (MPD). Cyst detection rates according to size, location, multiplicity, and body mass index (BMI) were compared using the Mann-Whitney test. Kappa statistics and interclass correlation coefficient were used to evaluate the inter-observer agreement of cyst detection and consistency of size and diameter of MPD between TAUS and CT/MRI.
**Results:** Detection rate of pancreatic cysts was 81.8 % (63/77) and 83.1% (64/77) for each radiologist. Detection rate of larger (≥10mm) cysts was significantly higher than smaller cysts (89.0% vs 63.6% for R1; 89.0% vs 68.2% for R2, $p < 0.05$). Non tail location showed significantly higher detection rate than tail location (89.5% vs 65.0% for R2, $p = 0.013$) and detection rate was also significantly higher in single than in multiple cysts (90.9% vs 69.7 %, $p = 0.018$ for R1). However, BMI showed no significant difference. Inter-observer agreement was excellent for cyst size (0.963, 95% CI: 0.943-0.976) and diameter of MPD (0.930, 95% CI: 0.891- 0.956).
**Conclusion:** TAUS could be a useful surveillance imaging tool for incidentally detected pancreatic cysts on CT or MRI, especially, in single pancreatic cysts of larger (≥1cm) size and non-tail location.

### SS 12.3

*Withdrawn by the authors*

### SS 12.4

### MDCT scan with model-based reconstruction algorithm in the detection and characterization of intraductal papillary mucinous neoplasm: comparison with MRCP

D. Ippolito[1], C. Maino[1], A. Pecorelli[1], M. Ragusi[1], T. Giandola[1], M. Porta[1], C. Talei Franzesi[1], S. Sironi[2]; [1]Monza/IT, [2]Milan/IT

**Purpose:** To compare the diagnostic accuracy of low-kV MDCT reconstructed with model-based iterative reconstruction algorithm (IMR) to MRI with MRCP sequences in the detection of intraductal papillary mucinous neoplasm (IPMN) and those features useful for their characterization.
**Material and methods:** We evaluated a total of 90 patients, with no history of pancreatitis, neoplasm and prior surgical procedures in the pancreas, who underwent both low-dose MDCT reconstructed with IMR and MRCP (time interval lower 90 days) for incidental pancreatic cystic lesions. Number of lesions detected in each patient and morphologic features (according to 2018 European Guidelines) of defined target lesion were recorded and analyzed to compare the diagnostic performance of MDCT with IMR algorithm and MRCP.
**Results:** A higher number of pancreatic cysts was identified on MRI than CT examinations (463 vs 227); however, high intraclass correlation coefficient (ICC) value of 0.76 was found, suggesting a good agreement. Very good agreement (ICC 0.98) was found regarding the diameter of target lesions (21.4 mm CT vs 21.8 mm MRI), location of the target lesion ($\kappa=0.90$), detection of MPD dilatation ($\kappa=1.00$), MPD communication ($\kappa=0.73$) and septa ($\kappa=0.86$). Fair agreement with calcifications ($\kappa=0.22$) was found.
**Conclusion:** Low-dose MDCT reconstructed with IMR can be considered equivalent to MRI with MRCP in the evaluation of IPMN, offering detailed morphologic features helpful for their characterization and those correlated with a high risk of malignancy.

### SS 12.5

### Role of MDCT in the assessment of acute pancreatitis and severity grading based on modified CT severity index and its correlation with severity grading as per revised Atlanta classification 2012 and clinical outcome

S. Ps; New Delhi/IN

**Purpose:** To study the role of MDCT in the assessment of the severity of acute pancreatitis using modified CT scoring system and correlate the MDCT scoring with clinical severity of the disease and patient outcome.
**Material and methods:** It was a cross-sectional observational study of 152 patients conducted in the Department of Radiodiagnosis at Dr. Ram Manohar Lohia Hospital. Severity grading of acute pancreatitis was assessed based on modified CT severity index (MCTSI). Clinical severity of acute pancreatitis was evaluated based on revised Atlanta classification (RAC) 2012. Clinical outcomes of patients were noted in terms of duration of hospital admission, ICU stay, evidence of organ failure, evidence of infection, the need for intervention and death.
**Results:** The study group included 152 cases of acute pancreatitis: according to MCTSI, 25 mild, 49 moderate and 78 severe cases; clinically, 27 mild, 76 moderately severe and 49 severe cases based on RAC. MCTSI showed good concordance with RAC grading in 23 (84.18%) mild, 40 (52.6%) moderate and 44 (89.7%) severe cases. Around 43.5% (34/78 cases) clinical moderately severe cases were overestimated by MCTSI as severe cases because of 2 extra points from extrapancreatic complication mainly due to mild pleural effusion and ascites even in the absence of organ failure. MCTSI showed significant statistical correlation ($p < 0.001$) with outcome parameters except for evidence of infection and death.
**Conclusion:** The present study MCTSI showed good concordance with clinical severity grading (RAC). MCTSI grading of acute pancreatitis is helpful for clinicians for prognostication and decision-making in the duration of hospital stay, need for ICU care, intervention and follow-up.

### SS 12.6

### Preoperative CT evaluation for the prediction of acute pancreatitis after pancreaticoduodenectomy and distal pancreatectomy

A. Fighera, M. Chincarini, G.A. Zamboni, G. Mansueto; Verona/IT

**Purpose:** To investigate CT risk factors for postoperative acute pancreatitis after pancreaticoduodenectomy and distal pancreatectomy.

**Material and methods:** We retrospectively reviewed the preoperative multiphasic CT scans of 19 patients with clinically confirmed postoperative acute pancreatitis (14 after pancreaticoduodenectomy and 5 after distal pancreatectomy) and 19 CT scans of patients who did not show acute pancreatitis after surgery (13 after pancreaticoduodenectomy and 6 after distal pancreatectomy). Scans were reviewed by two readers in consensus, measuring pancreatic duct size and parenchymal thickness of the portion of the pancreas which would be the pancreatic remnant after surgery. An ROI was placed in basal and arterial phases in the same portion of the pancreas to measure basal density and arterial enhancement at the level of the future remnant.

**Results:** Pancreatic duct size was significantly smaller in patients who did manifest postoperative acute pancreatitis, with a mean value of 2.1±0.3 mm vs 5.6±0.9 mm (p value<0.05). Pancreatic parenchymal thickness was higher in patients with postoperative pancreatitis: mean value 15.9±0.7 mm vs 10.9±1.1 mm, (p value<0.05). In patients with postoperative pancreatitis, we also noticed a significantly higher basal density with a mean value of 40.9±1.7 HU vs 32.7±2.4 HU (p value<0.05). Difference of enhancement between arterial and basal phases was not significantly different between the two groups.

**Conclusion:** Smaller main pancreatic duct size, higher parenchymal thickness and higher basal parenchymal density at the level of the future remnant pancreas are indexes of major pancreatic tropism. In consequence, they are risk factors for developing acute pancreatitis after pancreaticoduodenectomy or distal pancreatectomy.

### SS 12.7

### Risk assessment for postoperative pancreatic fistula after pancreaticoduodenectomy on preoperative CT

A. Grecchi[1], R. Menghini[1], G.A. Zamboni[1], A.J. Cibulsky[1], M. Gasparetto[1], M.-P. Vullierme[2], G. Marchegiani[1], G. Mansueto[1]; [1]Verona/IT, [2]Clichy/FR

**Purpose:** To assess the value of preoperative CT features for predicting clinically relevant postoperative pancreatic fistula (POPF).

**Material and methods:** This multicenter retrospective study included 88 patients who underwent pancreaticoduodenectomy in two centers. Patients were divided into 2 groups according to clinical data: 44 patients with POPF and 44 patients without POPF. In each center, one reader (≥10 years experience) reviewed the preoperative MDCTs of the patients, measuring at the planned resection plane the main pancreatic duct (MPD) diameter, the parenchymal density in the different phases and the parenchymal thickness. HUven-HUart was calculated, and progressive enhancement was interpreted as a sign of fibrosis. Body composition was analysed by calculating visceral adipose tissue (VAT), subcutaneous adipose tissue (SAT), and skeletal muscle area at the L2 level (ImageJ software). Retrorenal fat thickness and psoas density were measured. Fisher's test and Mann–Whitney test were used as appropriate.

**Results:** MPD diameter was 2.7±2.6mm in the fistula group and 6.3±3.1mm in the non-fistula group (P<0.0001). The mean attenuation difference between venous and arterial phases was 2.6HU in POPF group and -13.2HU in the non-POPF group (P=0.0010). VAT was 19164.2mm2 in the POPF group and 13263.5mm2 in the non-POPF group (P=0.002). SAT was 18018.6mm2 in POPF group and 12269.7mm2 in the non-POPF group (P=0.004). No difference was observed for other parameters.

**Conclusion:** Fibrosis, expressed by increasing enhancement of the normal pancreatic parenchyma, MPD diameter and increased VAT and SAT may express increased risk for pancreatic fistula after pancreaticoduodenectomy.

### SS 12.8

### Inter-observer variability of radiologists for Cambridge Classification for Chronic Pancreatitis using CT and MRCP: results from a large multi-centre study

T. Tirkes[1], A.K. Dasyam[2], J.R. Grajo[3], Z. Shah[4], N. Takahashi[5], S.N. Appana[6], L. Li[6], E.L. Fogel[1], S. Pandol[7], D. Conwell[4], D. Yadav[2]; [1]Indianapolis, IN/US, [2]Pittsburgh, PA/US, [3]Gainesville, FL/US, [4]Columbus, OH/US, [5]Rochester, MN/US, [6]Houston, TX/US, [7]Los Angeles, CA/US

**Purpose:** To determine inter-observer variability among radiologists in assigning Cambridge Classification (CC) for chronic pancreatitis (CP) based on secretin-enhanced MRCP and contrast-enhanced CT.

**Material and methods:** This multi-institutional study included 39 subjects who were selected randomly without reference to their clinical data, from 422 participants enrolled in the PROCEED study (ClinicalTrials.gov ID NCT03099850) between June 2017 and August 2018. These subjects were categorized into three groups based on clinical data and review of imaging by local radiologist (LR) at the enrolling sites: controls (n = 8; CC of 0), suspected CP (n = 22; CC of 1 or 2) and definite CP (n = 9; CC = 3 or 4). Each study was scored by 3 radiologists, who were randomly selected from 5 blinded subspecialized radiologists. We compared the CC score by LRs and a consensus CC score by central radiologists (CRs). The weighted kappa statistic (wKappa) was used to estimate the inter-observer agreement.

**Results:** There was moderate agreement (63% and 67%, respectively) between CRs and LRs in both CT score (wKappa [95% CI] = 0.56 [0.34, 0.78]; p-value = 0.57) and MR score (wKappa [95% CI] = 0.68 [0.49, 0.86]; p-value = 0.72). The composite CT/MRCP score showed moderate agreement (wKappa [95% CI] = 0.62 [0.43, 0.81]; p-value = 0.80). For majority of subjects, the actual cohort assignment by the LR agreed with consensus composite CT/MRCP score by the CRs, with concordance ranging from 75% to 89%.

**Conclusion:** There is a high degree of concordance by radiologists for the assignment of CC using MRCP and CT.

### SS 12.9

### Ultra-small superparamagnetic iron oxide-enhanced MRI for pre-operative lymph node staging in patients with pancreatic and periampullary carcinoma: a feasibility study

G. Litjens[1], A. Nakamoto[2], L. Brosens[1], E.V. Geenen[1], M. Maas[1], M. Prokop[1], T. Scheenen[1], P. Zámecnik[1], K.V. Laarhoven[1], J. Barentsz[1], J. Hermans[1]; [1]Nijmegen/NL, [2]Takatsuki/JP

**Purpose:** An important negative prognostic factor for adenocarcinomas of the pancreas and periampullary region is the presence of para-aortic lymph node (LN) metastases, which are regarded as distant metastases. Determining LN status is challenging but may be possible with MRI with ultra-small superparamagnetic iron oxide (USPIO) particles, which have proven valuable for detecting LN metastases of solid tumors. The aim is to validate USPIO-MRI for pathology in patients with pancreatic or periampullary cancer.

**Material and methods:** Analysis of an ongoing feasibility study in patients undergoing resection of pancreatic or periampullary adenocarcinoma (N=14). LNs are scored on MRI, divided between regional and distant, and matched to histopathology of resected specimens.

**Results:** One patient was excluded due to perioperative peritoneal metastases. In the remaining 13 patients, in total 307 LNs were detected on MRI. In total, 293 LNs were analyzed histopathologically. On MRI, 28/78 regional and 58/229 distant LNs were suspected for metastases. On histopathology, 35/260 regional and 3/33 distant LNs were positive for metastases. Not all suspected distant LNs were resected. The regional LNs were assessed on a per-patient basis. Five patients had suspicious LNs with positive LNs histopathologically, 4 patients had negative LNs on MRI, but positive LNs histopathologically and 4 patients had negative LNs on MRI with negative LNs histopathologically.

**Conclusion:** The preliminary results show that on patient basis it is possible to detect regional LN metastases with USPIO-MRI. Further analysis of the data, including node-to-node analysis and follow-up, will provide more insight into the value of USPIO-MRI in these patients.

### SS 12.10

### Intestinal type ampullary adenocarcinoma vs. primary duodenal adenocarcinoma: role of ampullary MDCT for preoperative differentiation

Z. Kovacevic, Belgrade/RS

**Purpose:** To evaluate the utility of ampullary MDCT in the non-invasive, preoperative differentiation of duodenal carcinoma and intestinal-type ampullary carcinoma.
**Material and methods:** In this retrospective study, 20 [8 females (age 38-77 years)] patients with resected ampullary adenocarcinoma and duodenal adenocarcinoma who underwent preoperative contrast-enhanced ampullary MDCT were included. Two radiologists, blinded to the pathological diagnosis of adenocarcinoma subtype, evaluated the presence of five MDCT features in consensus. Correlation between MDCT findings with ampullary cancer subtypes was performed using $\chi 2$ and Fisher's exact test.
**Results:** When evaluated with ampullary MDCT, intestinal-type ampullary adenocarcinomas were significantly different in terms of lesion morphology ($p<0.0001$), shape of the papilla ($p<0.0001$), common bile duct (CBD) infiltration (p: 0.003) and dilation (p: 0.0004) and duodenopancreatic groove infiltration (p: 0.0009). Duodenal carcinomas were more often infiltrative in morphology (9/10 [90%]), showed retracted papilla (8/10 [80%]), CBD (3/10[30%]) and main pancreatic duct (MPD) infiltration (2/10 [20%]), dilated CBD (3/10 [100%] and MPD (2/10 [20%]), and "fixed" duodenopancreatic groove appearance (5/10 [50%]). Intestinal-type ampullary carcinomas were significantly more frequently nodular in shape (10/10 [100%]), had a bulging papilla (9/10 [90%]), and "free" duodenopancreatic groove appearance (10/10[100]).
**Conclusion:** A tailored ampullary MDCT protocol can be useful to differentiate intestinal adenocarcinoma from duodenal adenocarcinoma preoperatively.

**11:00 - 12:30** **Room E 102**

*Scientific Session SS 13*

### Focal liver tumours: diagnosis and intervention

### SS 13.1

### Growth of hepatocellular adenoma during pregnancy: a prospective study

M.G. Thomeer, M. Gaspersz, A.J. Klompenhouwer, M. Broker, S. van Aalten, E. Steegers, T. Terkivatan, H. de Koning, R. de Man, J. Ijzermans; Rotterdam/NL

**Purpose:** To prospectively assess hepatocellular adenomas (HCA) smaller than 5 cm during pregnancy.
**Material and methods:** This is a multicenter prospective cohort study in pregnant women with suspicion of HCA smaller than 5 cm on imaging. Growth of HCA was closely monitored with US throughout pregnancy. Final diagnosis was based on MRI with hepatobiliary contrast or biopsy, preferably before pregnancy.
**Results:** 18 of 66 women were excluded from analysis because postpartum late contrast enhancement (LCE)-MRI did not confirm the diagnosis of HCA and showed the lesion to be focal nodular hyperplasia (FNH). The remaining 48 with a LCE-MRI confirming HCA were followed during 51 pregnancies. Median age was 30 years (IQR 27-33) and BMI 31.9 kg/m2 (IQR 26.3-36.6). Growth of HCA was seen in 13 of the pregnancies (25.5%); the median increase was 14mm (IQR 8-19). One woman whose HCA grew to >70mm successfully underwent transarterial embolization at week 26 of pregnancy to prevent further growth. The other 50 pregnancies proceeded without complications.
**Conclusion:** This study suggests that a HCA <5cm bears minimal risk for a pregnant woman and none for the child. As in a quarter of the cases the HCA increased in size during pregnancy, we recommend close monitoring with US examinations, enabling intervention if needed. In light of the large proportion of misdiagnosed HCA, LCE-MRI should be performed to prevent unnecessary anxiety in women with a benign liver lesion.

### SS 13.2

### B-mode US is more accurate than contrast-enhanced US for the subtyping of hepatocellular adenomas

J. Grégory[1], A. Paisant[2], L. Paulatto[1], L. Raynaud[1], C. Bertin[1], M.-P. Vullierme[1], V. Vilgrain[1], M. Ronot[1]; [1]Clichy/FR, [2]Angers/FR

**Purpose:** To compare the features of hepatocellular adenoma subtypes (HCAs) in B-mode and contrast-enhanced US (CEUS).
**Material and methods:** Thirty-four patients (31 women (91%), median age 43.5 years [27-67]) with 45 pathologically proven and subtyped HCAs (including 29 inflammatory HCAs (I-HCA) and 15 HNF1-α-inactivated HCAs (H-HCA)) who underwent CEUS were included. The features of HCA on B-mode and CEUS were independently reviewed by two radiologists and compared between HCA subtypes.
**Results:** I-HCAs were either hypoechoic (23/29, 79%) or isoechoic (4/29, 14%) with B-mode and 12 (41.4%) were heterogeneous. All H-HCAs except one demonstrated homogeneous hyperechogenicity (93%) ($p=0.001$). Moderate or marked liver steatosis was only observed in I-HCAs (12/29, 41%) ($p=0.001$). Arterial hyperenhancement was observed on CEUS in 27/29 (93%) I-HCAs and in 14/15 (93%) H-HCAs ($p=0.98$). Washout was present in 6/29 (21%) I-HCAs and 1/15 (7%) H-HCAs ($p=0.27$). A total of 23/29 (79%) I-HCAs and 15/15 (100%) HCAs were homogeneous on portal and delayed phase acquisitions ($p=0.04$). The positive predictive value for identifying an H-HCA was 100% when the lesion was homogeneous and hyperechoic on B-mode, and the negative predictive value was 100% if neither of these two features was present in a liver with obvious steatosis.
**Conclusion:** Most CEUS features, especially enhancement patterns, do not significantly differ between HCAs. CEUS does not seem to provide additional information from B-mode US. The combination of B-mode lesion hyperechogenicity, homogeneity and the absence of obvious liver steatosis is useful to distinguish H-HCAs from I-HCAs.

### SS 13.3

### Gadoxetic acid-enhanced MRI findings of hepatocellular adenomas in glycogen storage disease type 1

F. Vernuccio[1], S. Austin[2], P.S. Kishnani[2], V. Vilgrain[3], D. Marin[2]; [1]Palermo/IT, [2]Durham, NC/US, [3]Clichy/FR

**Purpose:** To investigate the gadoxetic acid-enhanced MRI findings of hepatocellular adenomas (HCAs) in patients with glycogen storage disease type 1 (GSD1).

**Material and methods:** All consecutive GSD1 patients with HCAs studied with gadoxetic acid-enhanced MRI were retrospectively included. Qualitative MRI analysis of the HCAs in the various sequences and contrast phases was performed.

**Results:** Eight consecutive GSD1 patients (mean age 26.6 years ± 7.6; 4 men, 4 women) with 47 seven HCAs (mean size 1.2 cm ± 0.9) were included. Seven of 8 patients (87.5%) had multiple lesions (range 2-16). Of the 47 HCAs, 5 (11%) showed a pseudocapsule, 9 (19%) fat content, 31(66%) hyperintensity on T2-weighted images, but lack of atoll sign, and 26 (55%) hyperintensity on T1-weighted images. Of note, peripheral rim distribution of fat was demonstrated in 2 HCAs. Arterial enhancement was documented in 42 (89%) of 47 HCAs (weak in 35 [83%] of 42), a portal venous washout in 6 (13%), lesion contrast retention in the hepatobiliary phase in 11 (23%), and perilesional contrast retention in the hepatobiliary phase in 16 (34%). HCAs <1 cm (28 of 47) were more homogeneous than HCAs ≥1 cm (19 of 47) in almost all sequences and contrast phases. A peripheral rim of contrast retention was documented in 6 (21%) of 28 HCAs <1 cm and in 10 (53%) of the 19 HCAs ≥1 cm.

**Conclusion:** HCAs in GSD 1 may show different MRI features compared to the general population, including anomalous fat distribution, lack of atoll sign, and weak arterial enhancement.

### SS 13.4

### Stereotactic body radiation therapy for HCC: multiparametric MRI to evaluate response to treatment and progression-free survival

R. Faletti, F. Guarasci, V. Ruggeri, M. Gatti, P. Fonio; Turin/IT

**Purpose:** To outline the role of multiparametric MR (mpMR) in the assessment of HCC response to stereotactic body radiation therapy (SBRT).

**Material and methods:** Twenty-six patients (19M and 7 F; average age 63.7 y.o.) were retrospectively enrolled for a total of 42 lesions. MpMR was performed with 1.5 T scanner and gadoxetic acid. Images were evaluated by two different readers. The continuous variables were subjected to the Shapiro-Wilk normality test; Wilcoxon test was used for the comparison between paired variables, while the Mann-Whitney test was used for the independent variables; progression-free survival (PFS) was evaluated by Kaplan-Meier curves.

**Results:** Comparison between pre-SBRT and post-SBRT shows different significant features at 1st mpMR: arterial tissue enhancement (18.9 vs 8.4 mm; p < 0.0001), diameter (20.3 vs 16.5 mm; p < 0.0001), T2w intensity (21 vs 8 lesions; p 0.006), DWI intensity (26 vs 8 lesions; p < 0.0001) and ADC value (1.0 vs 1.4; p < 0.0001). The only feature significantly changed at 2nd mpMR was diameter (16.5 vs 12.4 mm; p 0.008). According to mRECIST at 1st mpMR were found 2 cases of infield progression (5%) and 9 cases of outfield progression (21%). The Kaplan-Meier curve demonstrates a progressive reduction in the PFS, which reaches a plateau around 24 months.

**Conclusion:** The role of SBRT for the treatment of HCC is still not clearly defined. MpMR can provide early indications of treatment efficacy, in particular through DWI. SBRT is a valid alternative to other locoregional therapies, demonstrating excellent performance in terms of local disease control.

### SS 13.5

### Can intravoxel incoherent motion diffusion-weighted imaging be used for preoperative assessment of microvascular invasion in HCC?

T. Zhang, Y. Wei, B. Song; Chengdu/CN

**Purpose:** To prospectively evaluate the potential role of intravoxel incoherent motion (IVIM) and conventional radiologic features for preoperative prediction of microvascular invasion (MVI) in patients with HCC.

**Material and methods:** A cohort comprising 115 patients with 135 newly diagnosed HCCs were evaluated. For all examinations, studies were carried out using a 3.0 T MR system. The IVIM-diffusion-weighted (DW) MR imaging was performed before the injection of contrast agents. Twelve b values from 0 to 1000 sec/mm$^2$ (0, 10, 20, 40, 80, 100, 150, 200, 400, 600, 800 and 1000 sec/mm$^2$) were obtained. All the IVIM-DW images were analyzed by two independent radiologists blindly; the whole tumor volume was selected for the region of interest (ROI) measurement. The apparent diffusion coefficient (ADC), $ADC_{slow}$, $ADC_{fast}$ and f value were automatically calculated by the workstation, and the averaged value was used for further statistical analysis. Interobserver agreement was checked, and univariate and multivariate logistic regression were used for screening the risk factors. Receiver operating characteristics (ROC) curves analyses were performed to evaluate the diagnostic performance.

**Results:** Features significantly related to MVI of HCC at univariate analysis were reduced ADC (odds ratio, 0.341; p<0.001), $ADC_{slow}$ (odds ratio, 0.141; p<0.001) and irregular circumferential enhancement (odds ratio, 9.908; p<0.001). At multivariate analysis, only $ADC_{slow}$ (odds ratio, 0.096; p<0.001) was the independent risk factor for MVI of HCC. The mean $ADC_{slow}$ value for MVI of HCC showed an area under ROC curves of 0.815 (95% CI: 0.740-0.877).

**Conclusion:** The results of the preliminary study have demonstrated that the decreased ADCslow value was an independent risk factor for predicting MVI of HCC.

### SS 13.6

### Evaluation of a serum tumour marker-based recurrence prediction model after radiofrequency ablation for very early/early-stage HCC

D.H. Lee, J. Yoo; Seoul/KR

**Purpose:** A recent study showed that serum tumor marker-based MoRAL score (11×√[PIVKA-II] + 2×√[AFP]) can reflect the aggressiveness of HCCin explant liver. The present study aimed to evaluate whether baseline MoRAL score could predict tumor recurrence after radiofrequency ablation (RFA) for very early/early-stage HCC.

**Material and methods:** A total of 576 HCC patients who underwent RFA as initial treatment were enrolled from two tertiary-referral hospitals (256 patients in the development cohort and 320 patients in the validation cohort). The primary endpoint was recurrence-free survival (RFS) and the secondary endpoints included cumulative risks of intrahepatic distant recurrence (IDR) and extrahepatic metastasis (EM).

**Results:** In development cohort, MoRAL score was an independent prognostic factor of RFS (p=0.02). The optimal cutoff-MoRAL score for predicting RFS was 68. Patients with high MoRAL score (>68) showed significantly shorter RFS than did those with low MoRAL score (hazard ratio [HR]=2.09, p<0.001). The five-year RFS rates were 32.3% and 53.2% in high- and low-MoRAL groups, respectively. The risks of both IDR (HR=1.76, p=0.003; 5-year incidences=50.2% vs. 40.5%)) and EM (HR=8.25, p=0.006; 5-year incidences=19.7% vs. 3.4%) were also significantly higher in high-MoRAL group. These results were reproduced in the validation cohort: RFS (HR=1.81, p<0.001; 5-year RFS rates=27.7% vs 53.6%) was significantly shorter and risks of IDR (HR=1.59, p=0.003; 5-year incidences=42.7% vs. 63.9%) and EM (HR=6.19, p=0.004; 5-year incidences=11.8% vs. 1.4%) were significantly higher in high-MoRAL group.

**Conclusion:** A high MoRAL score of >68 was a significant predictive factor of tumor recurrence after RFA for very early/early-stage HCC. Moreover, it might be warranted to evaluate EM in patients with high baseline-MoRAL scores.

### SS 13.7

### Public availability of trial results assessing transarterial chemoembolisation for treating HCC

J. Grégory[1], V. Vilgrain[1], I. Boutron[2], M. Ronot[1]; [1]Clichy/FR, [2]Paris/FR

**Purpose:** To evaluate to what extent the results of registered randomized controlled trials (RCTs) assessing transarterial chemoembolization (TACE) for the treatment of HCC are publicly available.

**Material and methods:** We searched the Cochrane Central Register of Controlled Trials, the International Clinical Trials Registry Platform and primary registries for RCTs assessing TACE for the treatment of HCC, registered between January 2008 and August 2018, that had exceeded their completion date by more than one year. We searched PubMed, EMBASE and Google Scholar for a publication as well as the registry for results. The main outcomes were the availability of results, and the time to the first availability of results (i.e., posted on the registry or published). Secondary outcomes were the proportion of results publicly available at 12, 24 and 36 months after completion.

**Results:** Among 68 identified RCTs (11,751 participants), 26 had publicly available results (38%, i.e. 42% of participants). Results of 25 RCTs (37%) were published, with only three with results posted on the registry, and two with both published and posted results. The median (Q1-Q3) time from completion to the first public availability of results was 19 months (12-35). The cumulative percentage of RCTs with results available, either posted on the registry or published, was 10% (95% CI, 3% to 17%) at 12 months after completion of the RCTs, and 29% (95% CI, 17% to 39%) at 36 months.

**Conclusion:** Despite the ethical commitments and societal expectations for disclosure of results, the availability of results of RCTs on TACE for HCC is very limited.

### SS 13.8

### Prediction of response to trans-arterial chemoembolisation in HCC using a model based on pretreatment CT texture features

M.A. Tipaldi, E. Ronconi, D. Caruso, M. Rossi, A. Laghi; Rome/IT

**Purpose:** To determine whether texture features on pretreatment contrast material-enhanced CT images can predict treatment response to trans-arterial chemoembolization (TACE) in patients with HCC.

**Material and methods:** 97 patients with HCC treated with TACE, between September 2012 and August 2018, were retrospectively evaluated. Inclusion criteria for this study were available pretreatment laboratory exams and clinical data, pre-treatment CT performed in our hospital, absence of portal vein thrombosis and available post-treatment CT at 1-6 months from the procedure with no other pharmacological or interventional intercorring treatments. 41 pts met the inclusion criteria and were enrolled in the study. CT texture analysis was performed on pretreatment portal venous images and CT texture first-level features at different anatomic scales, ranging from fine to coarse texture, were analyzed. Post-treatment CT images were evaluated using modified RECIST criteria. A univariate analysis was performed via an appropriate test (Chi2, Mann-Whitney U, two-tailed paired T test) to identify those variables which differed significantly ($p<0.05$) in the two populations of complete responders and partial/non-responders. Optimal cut-off values were defined using ROC curves. Statistical analysis was performed with SPSS 24.

**Results:** Standard deviation, entropy, mpp, skewness and kurtosis variables resulted statistically significant between the two groups. A prediction model for complete response, using multivariate logistic regression with forward stepwise selection, was efficiently developed (AUC=0.702; $p<0.001$).

**Conclusion:** Pretreatment CT texture analysis features alone can help to predict complete responders from partial/non-responder patients to TACE treatment for HCC.

### SS 13.9

### Liver surface nodularity: a novel predictor of post-hepatectomy liver failure in patients with colorectal liver metastases following chemotherapy

A. Perrot, T. Yoh, F. Cauchy, A. Beaufrère, V. Paradis, O. Soubrane, V. Vilgrain, M. Ronot; Clichy/FR

**Purpose:** To (i) evaluate the relationship between liver surface nodularity (LSN) and chemotherapy-associated liver injury (CALI) in patients undergoing hepatectomy for colorectal liver metastases (CLM), and (ii) assess the relationship between LSN and postoperative liver failure (PHLF).

**Material and methods:** This is a single-center retrospective analysis of all patients with preoperative CT scans within 6 weeks before surgery and who were undergoing chemotherapy followed by hepatectomy for CLM between 2010 and 2017. LSN was determined on CT. The association between LSN, CALI and clinically relevant PHLF (i.e. ≥ grade B) was analyzed and optimal cut-off values were determined by ROC curve analysis. Multivariate analysis and propensity score matching analysis were performed to determine the influence of LSN on PHLF ≥ grade B.

**Results:** A total of 26 patients (10%) developed PHLF≥grade B. The mean LSN was 2.25 (1.51-3.34). On univariate analysis, LSN was associated with a higher rate of PHLF≥grade B ($p<0.001$). The optimal LSN cut-off value for detecting PHLF≥grade B was 2.5 (sensitivity, 57.7%; specificity, 77.0%, accuracy 75%, $p<0.001$). Among chemotherapy-related variables, LSN≥2.5 was significantly associated with prolonged chemotherapy (>6 cycles, p=0.018), but not with CALI. After propensity score matching, LSN remained significantly associated with an increased risk of PHLF≥grade B (p=0.006). Multivariate analysis identified LSN≥2.5 (OR: 4.554; 95%CI:1.986-10.754; p=0.004) as the best preoperative predictor of PHLF≥grade B in patients undergoing major hepatectomy.

**Conclusion:** LSN is an independent surrogate of clinically relevant PHLF in patients who undergo chemotherapy followed by hepatectomy for CLM and may provide a valuable additional tool in the preoperative assessment of these patients.

### SS 13.10

### Hepatobiliary MR contrast agents are useful to diagnose HCC in patients with Budd-Chiari syndrome

M. Van Wettere, L. Paulatto, O. Bruno, P.-E. Rautou, A. Plessier, V. Paradis, V. Vilgrain, M. Ronot; Clichy/FR

**Purpose:** To evaluate hepatobiliary phase (HBP) images for the discrimination between benign and malignant lesions in patients with Budd-Chiari syndrome (BCS).

**Material and methods:** The study included all patients admitted to our institution with a diagnosis of BCS and focal liver lesions on hepatobiliary contrast agent-enhanced MRI (HBCA-MRI) from 2000 to 2019. MR images were reviewed by two radiologists blinded to the diagnosis of the lesions. Patient and lesion characteristics were recorded, with a focus on the appearance on HBP. Lesions were compared using Chi-square, Fisher, Student's t or Mann-Whitney tests.

**Results:** 26 patients (mean 35±11 yr [range 13-65], 21 women [81%]) with 99 benign liver lesions and 12 HCC were analyzed. Patients with HCC were significantly older than those with benign lesions (mean 50±10 vs. 33±9 yr, p =.003), with higher alpha-fetoprotein (AFP) levels (3/4 [75%] vs. 1/22 [5%] with AFP>15ng/mL, $p<0.001$). Homogeneous lesion signal hypointensity on HBP was depicted in 14 lesions, including 12/12 (100%) HCC and 2/99 (2%) benign lesions ($p<0.001$). The majority of benign liver lesions showed either peripheral (n=52/99 [53%]) or homogeneous hyperintensity (n=23/99 [23%]) on HBP. Lesions with signal hypointensity on HBP in patients with an AFP serum level >15 ng/mL were all found to be HCCs.

**Conclusion:** The majority of benign lesions showed homogeneous or peripheral hyperintensity on HBP while all HCCs showed homogeneous signal hypointensity. HBP images are helpful for the differentiation between benign lesions and HCCs and outperform other sequences. They should systematically be acquired for the characterization of focal lesions in patients with BCS.

11:00 - 12:30 Room E 105 / 106

*Scientific Session SS 14*
### Technical advances in gastro-intestinal and abdominal imaging

### *SS 14.1*
### Opportunistic screening for osteoporosis by abdominal CT in a British population
S. Vadera, T.M. Osborne, V. Shah, J.A. Stephenson; Leicester/UK

**Purpose:** It has previously been shown that CT scans performed for other indications can be used to identify patients with osteoporosis. This has not yet been tested in a British population. We sought to evaluate the use of vertebral CT attenuation measures for predicting osteoporosis in a British cohort, using dual-energy X-ray absorptiometry (DEXA) as a reference standard.
**Material and methods:** Patients who underwent abdominal CT in 2018, and concomitantly underwent DEXA within a six-month interval, were retrospectively included. CT attenuation values in Hounsfield units (HU) were measured on the sagittal reconstruction by the placement of a region-of-interest at the central portion of the L1 vertebral body, and then compared to their corresponding DEXA score. Receiver operating characteristic (ROC) curves were generated to evaluate the performance of a logistic regression model, and to determine sensitivity and specificity thresholds.
**Results:** 536 patients (394 females, mean age 65.8) were included, of which 174 had DEXA-defined osteoporosis. L1 attenuation measures were significantly different ($p<0.01$) between the three DEXA-defined groups of osteoporosis (118HU), osteopenia (143HU) and normal bone density (178HU). The area under the ROC curve was 0.74 (95% CI 0.69 – 0.78). A threshold of 169HU was 90% sensitive and a threshold of 104HU was 90% specific for diagnosing osteoporosis.
**Conclusion:** Routine abdominal CT can be used to opportunistically screen for osteoporosis without additional cost or radiation exposure. The thresholds identified in this study are comparable with previous studies in other populations. We recommend radiologists engage with primary care and rheumatology providers to determine appropriate cut-off values for further investigation.

### *SS 14.2*
### Improved display of hepatic arterial anatomy using differential subsampling with Cartesian ordering with gadoxetic acid-enhanced MRI: comparison with single arterial phase MRI and CT angiography
Y. Wei, B. Song; Chengdu/CN

**Purpose:** To prospectively evaluate whether multiple arterial phase (MAP) images from patients who underwent differential subsampling with Cartesian ordering (DISCO) acquisition would improve the hepatic arterial display compared with single arterial phase (SAP) and CT angiography (CTA).
**Material and methods:** From 08/2017 to 08/2018, prospectively acquired data of 130 patients who were divided into two study parts were analyzed. Two readers independently assessed the hepatic arterial display on a four-point scale. The kappa test was used to determine the interobserver agreement. Chi-square or Fisher's exact test was used for the categorical variables. Student's t test or the Mann-Whitney U test was used for the comparison of the motion artifacts. Kruskal-Wallis test was used to compare the difference between arterial displays.
**Results:** A moderate-to-excellent interobserver agreement was obtained for hepatic arterial display (all kappa values > 0.65). For study part I, the mean arterial display scores for the common hepatic artery (CHA), proper hepatic artery (PHA), left hepatic artery (LHA), right hepatic artery (RHA), left gastric artery (LGA) and gastroduodenal artery (GDA) obtained with DISCO were higher than those obtained with SAP imaging (all $p<0.01$). For study part II, comparable image quality for the arterial display of CHA ($p=0.798$), PHA ($p=0.935$), LHA ($p=0.122$), RHA ($p=0.809$), LGA ($p=0.710$) and GDA ($p=0.659$) was obtained with DISCO and CTA.
**Conclusion:** In conclusion, the use of MAP acquisition with DISCO is superior to the use of SAP in hepatic arterial display and compares favorably with CTA; in the future, DISCO possibly can replace the latter ionization-related method to provide a more comprehensive evaluation of the liver lesions.

### *SS 14.3*
### Image quality analysis of two different techniques for multi-arterial phase dynamic study in liver MRI with gadoxetic acid: results of a monocentric prospective randomised observational study
F. Castagnoli[1], V. Ruggeri[2], M. Bertuletti[1], M. Gatti[2], R. Inchingolo[3], R. Faletti[2], L. Grazioli[1]; [1]Brescia/IT, [2]Turin/IT, [3]Matera/IT

**Purpose:** To evaluate the technical performance of two different multi-arterial phase techniques in liver MRI.
**Material and methods:** From February 2017 to December 2018, we enrolled 273 randomized patients who underwent liver MRI using CAIPIRINHA (Controlled Aliasing in Parallel Imaging Results in Higher Acceleration) or TWIST-VIBE (Siemens, Erlangen, Germany) before and after the administration of gadoxetic acid (Gd-EOB-DTPA) (dose: 0.025ml/kg; injection rate: 1ml/s). The acquisition time of both sequences is superimposable (<20s). The protocol includes 3 arterial phases (6.5s each for CAIPIRINHA; 9s, 4s, 4s for TWIST-VIBE) with the possibility of reducing them depending on the patient's fitness level. The images were evaluated by two dedicated radiologists, who quantify Gibbs artifacts and noise (present/absent), breath artifacts and general quality of images using a score from 1 to 5 (1 "non-diagnostic" and 5 "absence of artifacts"/"optimal exam quality").
**Results:** There is no statistically significant difference between the demographic data of CAIPIRINHA and TWIST-VIBE cohort. CAIPIRINHA has always a better quality score ($p>0.02$) but we could not obtain a three-arterial-phase study in all patients (3%). TWIST-VIBE has worse Gibbs and breath score artifact but better noise score.
**Conclusion:** CAIPIRINHA always allows to obtain a diagnostic image, rarely at the expense of the three arterial phases (0% with one phase, 3% with two phases, 97% with three phases). TWIST-VIBE always allows to obtain three arterial phases. CAIPIRINHA better tolerates artifacts at the detriment of image resolution; TWIST-VIBE has a better signal-to-noise ratio but greater susceptibility to artifacts. The choice of one or the other must take into account patient's compliance and physical characteristics.

### *SS 14.4*
### Mapping of the liver function: impact of MR field strength on T1 reduction rate
D. Catucci, V. Obmann, A. Berzigotti, J. Hrycyk, W. Kajdi, C. Gräni, L. Ebner, J. Heverhagen, A. Christe, A.T. Huber; Bern/CH

**Purpose:** To determine whether T1 reduction rate is dependent on field strength in patients with and without liver cirrhosis.
**Material and methods:** 85 consecutive gadoxetic acid (Gd-EOB-DTPA) liver MRI scans with available T1 mapping pre- and post-contrast administration in the equilibrium phase were analyzed between September 2018 and January 2019. 44 exams were performed on a 1.5T system and 41 exams on a 3T system. Each of these two groups was then divided into patients with and without liver cirrhosis. T1 reduction rates were calculated as (native T1 – post-contrast T1)/native T1. Groups were compared using a Mann-Whitney U test.
**Results:** At 1.5T 23 patients had cirrhosis, while 21 patients had no cirrhosis. At 3T 17 patients had cirrhosis, while 24 patients had no cirrhosis. At both 3T and 1.5T T1 reduction rate discriminated between patients with and without cirrhosis ($p=0.001$ and $p>0.001$). T1 reduction rates did not differ significantly between 3T and 1.5T in patients with cirrhosis (median 60% vs. 59%; $p=0.991$) and without cirrhosis (median 75% vs. 75%; $p=0.787$).
**Conclusion:** This study shows that T1 reduction rate is comparable between 3T and 1.5T and may discriminate patients with and without liver cirrhosis as an imaging surrogate to map liver function.

### SS 14.5

### Effect of scanner selection and tube parameters on CT texture analysis: evaluation on ex vivo pig liver

R. Valletta[1], G.A. Zamboni[1], M. Bonatti[2], F. Lombardo[3], G. Mansueto[1]; [1]Verona/IT, [2]Bolzano/IT, [3]Negrar/IT

**Purpose:** To assess if tube parameters and scanner type affect the results of CT texture analysis parameters performed on pig liver parenchyma.
**Material and methods:** We performed 15 consecutive CT scans of a pig liver, using two different scanners (Brilliance64 and Brilliance6, Philips). Ten scans were performed on Brilliance64 using every possible combination of tube current (25, 50, 100, 150 or 200mAs) and voltage (80 or 120kVp), whereas five scans were performed on Brilliance6 at 120kVp varying only the tube current. CT texture analysis was performed on each scan with LifeX software (http://www.lifexsoft.org) by drawing 4 volumetric ROIs. Texture features of each scan were compared using Welch's ANOVA test and Brown-Forsythe ANOVA test.
**Results:** Several texture parameters were significantly different between the various scans performed on the two scanners. Regarding first-order texture parameters, skewness, energy and entropy were significantly different on scans acquired with the same voltage and current settings on different scanners, whereas entropy and energy were significantly different on scans acquired with different voltage or tube current settings on the same scanner. The large majority of second and higher order texture parameters appeared to be significantly affected by voltage, current and scanner selection.
**Conclusion:** CT textural parameters showed significant variability, depending on both CT scanner and scanning parameters. Our results need to be confirmed on a larger series, but they instill many doubts about the reproducibility and the reliability of texture analysis parameters.

### SS 14.6

### Correlation between medical outcome and US, shear-wave elastography and MRI findings in native liver survivor patients with biliary atresia after Kasai portoenterostomy

M. Caruso, R. Cuocolo, F. Di dato, C. Mollica, G. Vallone, V. Romeo, M. Petretta, R. Liuzzi, P.P. Mainenti, R. Iorio, A. Brunetti, S. Maurea; Naples/IT

**Purpose:** Biliary atresia (BA) is an obliterative cholangiopathy and Kasai portoenterostomy (KP) represents its first-line treatment. Clinical and laboratory parameters together with abdominal US are usually performed during the follow-up. Shear-wave elastography (SWE) is able to evaluate liver parenchyma stiffness and MRI has also been proposed to study these patients. The aim of our study is to correlate US, SWE and MRI findings with medical outcomes in native liver survivor patients with BA after KP.
**Material and methods:** Twenty-four patients were retrospectively enrolled and divided into two groups based on "ideal" (n=15) or "non-ideal" (n=9) medical outcome defined according to clinical and laboratory parameters. US, SWE and MRI exams were analyzed qualitatively and quantitatively for imaging signs suggestive of chronic liver disease (CLD).
**Results:** Significant differences were found in terms of liver surface (p= 0.007) and morphology (p= 0.013), portal vein diameter (p= 0.012) and spleen size (p= 0.002) by US, liver signal intensity (p= 0.013), portal vein diameter (p= 0.010), presence of portosystemic collaterals (p= 0.042) and spleen size (p= 0.001) by MRI. The evaluation of portal vein diameter (moderate, κ= 0.44), portosystemic collaterals (good, κ= 0.78) and spleen size (very good, κ= 0.92) showed the best agreement between US and MRI. A significant (p= 0.01) difference in liver parenchyma stiffness by SWE was also found between the two groups (cut-off=9.6 kPa, sensitivity=55.6%, specificity=100%, area under the ROC curve=0.82).
**Conclusion:** US, SWE and MRI findings correlate with the medical outcome in native liver survivor patients with BA after KP.

### SS 14.7

### Biomarkers derived from 18F-fluorodeoxyglucose positron emission tomography and conventional imaging provide prognostic information in patients with resected colorectal liver metastases

J. Franklin[1], C. Kartsonaki[2], F.V. Gleeson[2]; [1]Bournemouth/UK, [2]Oxford/UK

**Purpose:** Patients with oligometastatic colorectal liver metastases (CRLM) may be considered for metastasectomy. Outcomes are variable, and there is interest in developing prognostic biomarkers in this setting. Biomarkers derived from 18F-fluorodeoxyglucose positron emission tomography (18F-FDG-PET) have been found to be prognostically relevant in other tumour types. The purpose of this study was to test whether FDG-PET-derived parameters are prognostically relevant following metastasectomy for CRLM.
**Material and methods:** 96 patients who underwent metastasectomy for CRLM were included. Clinical parameters and the size, number and distribution of metastases on baseline imaging were recorded. FDG-PET parameters of CRLM and background liver parenchyma were derived. Cox proportional hazards models were used to estimate the associations between clinical and imaging variables with the endpoints disease-free survival (DFS) and hepatic-progression-free survival (hPFS).
**Results:** At univariate analysis, greater number of metastases (HR=1.15/metastasis, p=0.016) and bilobar metastases (HR=2.09, p=0.035) were associated with poorer DFS, and a higher number of metastases (HR=1.25/metastasis, p<0.001) and bilobar metastases (HR=3.80, p<0.001) were associated with poorer hPFS. There was a borderline significant effect of higher tumour SUVmean (HR=1.41, p=0.068) and higher background liver SUVmax (HR=2.04, p=0.069) on worse hPFS. At multivariate analysis, of the imaging-derived variables, a greater number of metastases, smaller metastases, and a higher tumour SUVmean (all p<0.05) were associated with worse DFS, and a greater number of metastases (p<0.001) was associated with worse hPFS.
**Conclusion:** The number of metastases by imaging was consistently associated with poorer outcomes in patients with resected CRLM. FDG-PET-derived biomarkers from the tumour and background liver were associated with poorer outcomes, which may reflect adverse tumour biology.

### SS 14.8

### Cryoablation for abdominal tumoral implants: a case series

B.M. Aarts, T.R. Baetens, A. Solouki, J. Vanrusselt, F. Imani, R.G.H. Beets-Tan, E.G. Klompenhouwer, F. Gómez Muñoz; Amsterdam/NL

**Purpose:** Percutaneous cryoablation (CA) is widely used for the treatment of primary cancers and metastases with excellent outcomes. Results of CA for abdominal tumoral implants are limited. The purpose of this report is to show our preliminary results of CA of abdominal implants.
**Material and methods:** A retrospective analysis was performed of metastatic patients treated by means of CA for an abdominal tumoral implant between November 2018 and October 2019. All patients were discussed in a multidisciplinary tumour board. Complete ablation was defined as no local tumour enhancement on the first follow-up imaging. Adverse events (AE) were registered according to the SIR classification.
**Results:** Eight patients received CA for an abdominal tumoral implant of their renal cancer (n=3), colorectal cancer (n=2), endometrial cancer (n=2), granulosa cell cancer (n=1) and lung cancer (n=1). Abdominal implants were located retroperitoneally (n=4), in the abdominal wall (n=2), pancreas (n=1) and anterior of the stomach (n=1). Median size of the implants was 1.7 cm (R 1.1-3.7cm). Complete ablation was achieved in all lesions. One patient developed an AE grade 2 consisting of psoas muscle pain that was successfully treated with medication.
**Conclusion:** Cryoablation in the abdomen can be safely and effectively used for tumour control in an oligometastatic setting.

### *SS 14.9*

*Withdrawn by the authors*

### *SS 14.10*

**Is there an association between the CT aspect of porto-systemic collaterals in cirrhotic patients and the risk of variceal haemorrhage?**

C. Caraiani, B. Petresc, A. Pop; Cluj-Napoca/RO

**Purpose:** To determine if the aspect of the collateral, porto-caval circulation, as detected by CT can be associated with the risk of developing variceal hemorrhage.

**Material and methods:** CT scans of 105 cirrhotic patients were retrospectively included in the study. Patients with unequivocal diagnosis of liver cirrhosis (established by clinical, biological, imaging or histopathological arguments) were admitted for the follow-up of liver cirrhosis. Images were retrospectively analyzed by two readers with experience in the field of abdominal radiology. Diameter of the left gastric vein, presence or absence and dimensions of oesophageal and gastric varices, paraumbilical veins and splenorenal vein, respectively, were the indicators assessed by CT.

**Results:** The diameter of the paraoesophageal veins was significantly higher in patients with upper GI bleeding (4 mm vs. 2.5 mm, p=0.02). Moreover, there was a significant association between the diameters of the paragastric veins and the presence of variceal hemorrhage (p=0.04). The logistic regression analysis showed that only the diameter of the left gastric vein can independently be associated with the risk of upper GI bleeding (OR=1.6 [95%CI: 1.17-2.19], p=0.003). We found an optimal cut-off value of 4 mm for the diameter of the left gastric vein, useful to discriminate among patients with variceal hemorrhage from the ones without it, with a good diagnostic performance (AUC=0.78, Se=97%, Sp=45.8%, PPV=55.2%, NPV=95.7%).

**Conclusion:** There is a strong association between CT parameters and the risk of upper gastrointestinal bleeding. Diameter of the left gastric vein has good sensitivity and negative predictive value for variceal hemorrhage.

### A

Aarts B.M.: **SS 14.8**
Abyzov A.: SS 1.8
Adnerhill I.: SS 6.10
Aerts H.J.W.L.: SS 5.4
Ahmad R.: SS 2.3
Allocca M.: SS 6.4, SS 6.7
Ambrosini I.: **SS 4.2**
An J.Y.: SS 10.2
Andrade F.: **SS 11.5**
Appana S.N.: SS 12.8
Armstrong E.M.: SS 3.6
Aroori S.: SS 3.6
Aubé C.: SS 9.3
Austin S.: SS 13.3
Azamat I.F.: SS 8.9
Azamat S.: **SS 8.9**

### B

Ba-Ssalamah A.: SS 11.8
Bae J.S.: SS 2.9
Baetens T.R.: SS 14.8
Bagnacci G.: **SS 9.2**
Bakers F.: SS 5.4
Bakir B.: SS 8.9
Balbo Mussetto A.: SS 8.8, SS 11.7
Baldwin R.: SS 2.2
Banz V.: SS 7.1
Barentsz J.: SS 12.9
Barnes D.: SS 2.5
Bartolotta T.V.: SS 4.1, SS 4.2
Bassett P.: SS 2.2
Bastati-Huber N.: SS 11.8
Batch K.: SS 5.10
Beaufrère A.: SS 13.9
Beer L.: **SS 11.8**
Beets G.: SS 8.10
Beets-Tan R.G.H.: SS 4.4, SS 4.7, SS 4.9, SS 5.1, SS 5.4, SS 5.6, SS 8.1, SS 8.10, SS 14.8
Beleù A.: SS 3.1, SS 3.8, SS 7.5, **SS 7.10**
Bellini D.: SS 2.4
Bertin C.: SS 13.2
Bertuletti M.: SS 14.3
Berzigotti A.: SS 1.1, **SS 7.7**, SS 14.4
Besnier L.: **SS 9.3**
Bezuidenhout A.F.: **SS 3.9**
Bican Y.: SS 11.8
Billimoria V.: SS 2.3
Boekestijn B.: **SS 3.4**
Boellaard T.: SS 8.10
Bonatti M.: SS 14.5
Booker M.T.: SS 10.2
Bordin D.S.: SS 3.3
Borrelli S.: SS 6.9
Bos P.: SS 4.7
Bosch J.: **SS 7.7**
Bouattour M.: SS 10.4
Boulay Coletta I.: SS 9.7
Bouma G.: SS 6.8
Boutron I.: SS 13.7
Brancatelli G.: SS 10.6, SS 10.10
Bredenoord A.: SS 6.8
Brogna B.: SS 6.9
Broker M.: SS 13.1
Brosens L.: SS 12.9
Brunetti A.: SS 14.6
Brunisng R.L.: SS 10.2
Bruno O.: SS 11.9, SS 13.10
Buchpiguel C.A.: SS 2.7
Burling D.: SS 2.2, SS 6.6
Buskens C.: SS 6.1

### C

Calendra A.: SS 1.6
Cannataci C.: **SS 7.8**
Cannella R.: **SS 4.1**, SS 4.2, **SS 10.6**, SS 10.10
Capelli P.: SS 3.7
Capozzi N.: SS 6.2
Cappello G.: SS 4.1, SS 4.2, SS 8.7
Cappendijk V.: SS 5.4, SS 8.1
Caraiani C.: **SS 14.10**
Carbone I.: SS 2.4
Cardobi N.: SS 3.7, SS 3.8
Caruso D.: SS 2.8, SS 5.9, SS 13.8
Caruso M.: **SS 14.6**
Caruso S.: SS 7.8
Casal-Dujat L.: SS 6.10
Casotti V.: SS 11.1
Castagnoli F.: **SS 14.3**
Castera L.: SS 11.5
Castier Y.: SS 9.4
Castro-Poceiro J.: SS 6.2
Cathomas M.: SS 7.9
Catucci D.: **SS 14.4**
Cauchy F.: SS 10.4, SS 11.3, SS 13.9
Chan C.: SS 7.2
Chan M.V.: SS 7.2
Chandramohan A.: **SS 8.4**
Chang W.: SS 4.8
Chincarini M.: SS 12.6
Chiu W.H.K.: SS 5.2
Cho J.: SS 5.10
Choi J.: SS 1.10
Choi S.-Y.: SS 10.8
Choi S.H.: SS 10.3
Christe A.: SS 1.1, SS 14.4
Christensen B.: SS 6.4
Cibulsky A.J.: SS 12.7
Cirillo S.: SS 8.7, SS 8.8, SS 11.7
Colledan M.: SS 11.1
Connor A.: SS 5.3
Conwell D.: SS 12.8
Corcos O.: SS 9.4
Corno L.: SS 9.7
Corr A.: **SS 6.6**
Costanza C.: SS 8.7
Cuocolo R.: SS 14.6

### D

D'Antiga L.: SS 11.1
D'Arrigo B.: SS 5.9
D'Haens G.R.A.M.: SS 6.5, SS 6.7
D'Onofrio M.: SS 3.1, SS 3.7, SS 3.8, SS 7.5, SS 7.10
Dagoglu Kartal M.G.: SS 8.9
Dagoglu Sakin R.N.: SS 8.9
Dallongeville A.: **SS 9.7**
Das A.: SS 11.4
Dasyam A.K.: SS 12.8
Dautt-Medina P.M.: SS 10.2
De Bie S.: SS 5.4
De Domenico R.: SS 9.9
de Jonge C.: SS 6.8
de Koning H.: SS 13.1
De Lucia S.: SS 6.9
de Man R.: SS 13.1
De Robertis R.: **SS 3.7**, SS 3.8, SS 7.5
De Ville De Goyet J.: SS 11.1
De Voogd F.: **SS 6.4**, **SS 6.5**, **SS 6.7**
Debray D.: SS 11.1
Defeudis A.: SS 4.2, SS 8.7
Delli Pizzi A.: SS 8.10
Denys A.: SS 4.10
Desana B.: SS 8.8, SS 11.7
Dewit L.: SS 4.4, SS 4.7
Di dato F.: SS 14.6
Di Piazza A.: SS 7.8
Digklia A.: SS 4.10
Dioguardi Burgio M.: **SS 1.8**, SS 11.5
Do R.K.G.: SS 5.10
Donker M.: SS 4.4, SS 4.7
Dromain C.: SS 4.10
Drudi A.: SS 3.1, SS 7.5, SS 7.10
Dubtsova E.A.: SS 3.3
Duczkowska M.: **SS 1.7**
Duran R.: SS 4.10
Durmaz Ugurcan O.: SS 11.1
Duseja A.: SS 11.4

### E

Ebner L.: SS 1.1, SS 14.4
Elkrief L.: SS 11.5
Erbs E.: SS 2.6
Ernst O.: SS 9.6

### F

Fabris C.: SS 8.10
Faletti R.: **SS 4.5**, SS 8.7, **SS 13.4**, SS 14.3
Fayeulle V.: **SS 9.6**
Fernandez-Clotet A.: SS 6.2
Festa A.: SS 6.9
Fighera A.: **SS 12.6**
Fink N.: SS 7.3, **SS 7.6**
Fischer S.: SS 5.3
Fleischmann D.: SS 9.5
Fogel E.L.: SS 12.8
Fonio P.: SS 4.5, SS 13.4
Fontanella G.: **SS 6.9**
Fork F.-T.: **SS 6.10**
Fornari A.: SS 8.8, SS 11.7
Fowler K.J.: SS 10.2
Franchi M.: **SS 8.8**, SS 11.7
Franchi-Abella S.: **SS 11.1**
Franklin J.: **SS 14.7**

### G

Gallinger S.: SS 5.3
Gangai N.: SS 5.10
Garteiser P.: SS 1.8, SS 11.5
Gasparetto M.: SS 12.7
Gaspersz M.: SS 13.1
Gatti M.: SS 4.5, SS 8.7, SS 13.4, SS 14.3
Gazit L.: SS 5.10
Gecse K.: SS 6.5, SS 6.7
Geenen E.V.: SS 12.9
Geenen R.: SS 5.4, SS 8.1
Germain A.: SS 8.5
Giandola T.: SS 12.4
Giannini V.: SS 4.2, SS 8.7
Giannotti G.: SS 3.1, **SS 3.8**
Giaretta A.: SS 3.1, **SS 7.5**, SS 7.10
Gleeson F.V.: SS 14.7
Goller S.: SS 7.6
Gonzales E.: SS 11.1
Gozzo C.: SS 4.1, SS 4.2
Grajo J.R.: SS 12.8
Gräni C.: SS 1.1, SS 14.4
Grazioli L.: SS 14.3
Grecchi A.: **SS 12.7**
Greco S.: SS 10.6, SS 10.10
Grégory J.: **SS 13.2**, **SS 13.7**
Gómez Muñoz F.: SS 5.1, SS 14.8
Guarasci F.: SS 13.4
Guerin F.: SS 11.1
Guerrini S.: SS 9.2
Guido G.: SS 2.8
Gulati A.: SS 11.4
Gulbay M.: **SS 1.5**

### H

Haak H.E.: **SS 8.10**
Haberlay M.: SS 9.6
Haghnejad V.: SS 8.5
Halligan S.: SS 2.2
Hamel J.F.: SS 9.3
Hamy A.: SS 9.3
Han J.K.: SS 3.10, SS 4.8, SS 5.5, SS 5.8
Han S.: **SS 3.10**
Hansen T.F.: SS 2.6
Hart A.: SS 6.6
Harun I.: SS 7.3
Hayoz R.H.: **SS 4.10**
Hermans J.: **SS 12.9**
Heverhagen J.: SS 7.1, SS 7.9, SS 14.4
Hierro L.: SS 11.1
Hocquelet A.: SS 4.10
Hodnett R.M.: **SS 3.6**
Hollings N.P.: SS 9.8
Horsthuis K.: SS 6.8
Hrycyk J.: SS 14.4
Huber A.T.: **SS 1.1**, SS 14.4
Huo Y.R.: **SS 7.2**
Hur B.Y.: SS 2.9, SS 10.3

### I

Iannicelli E.: SS 2.8
Ibrahim R.A.K.: SS 3.6
Idilman I.S.: SS 1.4
Ijzermans J.: SS 13.1
Imani F.: SS 5.1, SS 14.8
Imbriani M.: SS 9.9
Inchingolo R.: SS 14.3
Ingenerf M.K.: **SS 7.3**, SS 7.6
Iorio R.: SS 14.6
Ippolito D.: SS 12.4
Ivanovic A.: **SS 3.5**

### J

Jang S.: SS 3.10
Jankovic A.: **SS 8.1**
Jenkins I.: SS 6.6
Jensen L.H.: SS 2.6
Jeon S.K.: **SS 5.5**, **SS 10.3**
Jepson S.: SS 2.3
Jhaveri K.: SS 5.3
Jiang H.-Y.: SS 1.9, SS 10.1
Jo S.J.: SS 8.2
Joo I.: SS 3.2, SS 4.8
Joshi H.: SS 6.5
Julea F.: SS 1.8
Jurczyk K.: SS 1.7

### K

Kajdi W.: SS 14.4
Kalra N.: **SS 11.4**
Kamaya A.: SS 9.5
Kanavaki I.: SS 11.1
Kang H.-J.: SS 4.3, **SS 4.8**, SS 10.3, SS 12.2
Kang J.H.: **SS 1.10**
Kang K.A.: **SS 1.2**
Karaman Ş.: SS 8.9
Kartsonaki C.: SS 14.7
Keskin M.: SS 8.9
Khaled W.: SS 9.7
Kim D.H.: SS 1.10, **SS 9.8**
Kim J.H.: SS 3.2, SS 3.10, SS 4.8, SS 5.5, **SS 12.2**
Kim K.W.: SS 1.10
Kim S.H.: **SS 2.9**, **SS 4.3**, **SS 8.2**, SS 10.8
Kim S.Y.: SS 1.10
Kim-Fuchs C.: SS 7.9
Kishnani P.S.: SS 13.3
Klompenhouwer A.J.: SS 13.1
Klompenhouwer E.G.: SS 5.1, SS 14.8
Knebel J.-F.: SS 4.10
Kochhar R.: SS 4.6
Koh D.-M.: SS 5.3
Korff S.: SS 11.1
Kovacevic Z.: **SS 12.10**
Kucharzik T.: SS 6.4
Kuleznewa J.V.: SS 3.3
Kulle C.B.: SS 8.9

### L

Laarhoven K.V.: SS 12.9
Lachenmayer A.: SS 7.1, SS 7.9
Laghi A.: SS 2.4, SS 2.8, SS 5.9, SS 13.8
Lahaye M.J.: SS 5.4, SS 8.1, SS 8.10
Lambregts D.M.J.: SS 4.4, SS 4.7, SS 5.4, SS 5.6, SS 8.1, SS 8.10
Lampichler K.: SS 11.8
Landolfi F.: **SS 2.8**
Lario C.V.: SS 8.8, SS 11.7
Lauber D.: SS 11.8
Laurent V.: SS 8.5

Layyous N.: SS 10.2
Leander P.: SS 6.10
Leclerc J.: SS 8.5
Lee D.H.: **SS 1.3**, SS 7.4, **SS 13.6**
Lee E.S.: SS 12.2
Lee J.: SS 10.8
Lee J.B.: SS 1.10
Lee J.M.: SS 5.8, SS 10.3
Lee S.S.: SS 1.10, SS 10.3
Lesage C.: SS 9.3
Lesko K.A.: **SS 3.3**
Li L.: SS 12.8
Lim Y.-S.: SS 1.10
Lindebjerg J.: SS 2.6
Lioznovs A.: SS 11.10
Lipsich J.: SS 11.1
Litjens G.: SS 12.9
Liuzzi R.: SS 14.6
Lombardo F.: SS 14.5
Long M.G.: SS 9.8
Longo C.: SS 7.10
Lopez-Santamaria M.: SS 11.1
Low H.M.: SS 1.4
Lu C.: SS 6.4, SS 6.7
Luca A.: **SS 7.7**
Lucarelli A.: **SS 3.1**, SS 7.5, SS 7.10
Lung P.: SS 6.6
Lupton K.: SS 5.10
Lurz E.: SS 11.1

### M

Maas M.: SS 4.4, SS 4.7, SS 4.9, SS 5.1, SS 8.1, SS 8.10, SS 12.9
Maaser C.: SS 6.4, SS 6.7
Macera A.: SS 8.8, SS 11.7
Maconi G.: SS 6.4
Maggiori L.: SS 9.4
Magnusson M.: SS 11.1
Mainenti P.P.: SS 14.6
Maino C.: **SS 12.4**
Mamidipalli A.: SS 10.2
Mamone G.: SS 7.8
Mancinelli M.: SS 6.9
Mandorfer M.: SS 11.8
Manganiello C.A.T.: SS 6.9
Manoharan P.: SS 4.6
Mansueto G.: SS 12.6, SS 12.7, SS 14.5
Marchegiani G.: SS 12.7
Marin D.: SS 13.3
Maruzzelli L.: **SS 7.7**, SS 7.8
Masih D.: SS 8.4
Maskell G.F.: SS 9.8
Maurea S.: SS 14.6
Maurer M.: **SS 7.1**, **SS 7.9**
Mazzarotto R.: SS 3.7
Mazzei M.A.: SS 9.2
Mazzetti S.: SS 8.7
Mclin V.: SS 11.1
Meek D.: SS 5.1
Melenhorst J.: SS 8.10
Menghini R.: SS 12.7
Mercer J.: **SS 4.6**
Mertineit N.: SS 7.9
Micilotta M.: **SS 8.7**, SS 8.8, SS 11.7
Midiri M.: SS 10.6, SS 10.10
Miętkiewski J.: SS 1.7
Mihl C.: SS 8.10
Milazzo M.: SS 7.8
Miles G.: SS 3.6
Millet I.: SS 9.7
Min J.H.: **SS 10.8**
Min L.A.: **SS 4.4**, **SS 4.7**, SS 5.6
Miraglia R.: **SS 7.7**, SS 7.8
Mistry H.: SS 4.6
Mittal R.: SS 8.4
Molinar D.: SS 8.8, SS 11.7
Mollica C.: SS 14.6
Monetti F.: SS 9.9
Monraats M.: SS 6.1
Montemezzi S.: SS 3.7
Mortele K.J.: SS 3.9
Moulton C.-A.: SS 5.3
Mozer-Glassberg Y.: SS 11.1
Mulcahy K.: SS 2.3
Mullineux J.: SS 2.3
Muscogiuri E.: SS 5.9

### N

Nahas S.C.: SS 2.7
Najdawi M.: **SS 9.4**
Nakamoto A.: SS 12.9
Naves A.: SS 2.7
Nguyen H.: SS 5.10
Nikitin B.S.: SS 3.3
Nivolli A.: SS 11.9
Novak K.: SS 6.4, SS 6.7
Nuzzo A.: SS 9.4
Nylund K.: SS 6.4

### O

Obaro A.: **SS 2.2**
Obmann V.: SS 1.1, SS 14.4
Ocalan D.T.: SS 1.5
Ordás I.: SS 6.2
Orlandi P.E.: SS 9.9
Orlova N.V.: SS 3.3
Orry X.: SS 8.5
Ortega C.D.: **SS 2.7**
Orton M.: SS 5.3
Osano S.: SS 8.8
Osborne T.M.: **SS 2.3**, **SS 2.5**, SS 14.1

### P

Paiella S.: SS 3.7
Paisant A.: SS 9.3, SS 13.2
Palmela C.: SS 6.4
Pandol S.: SS 12.8
Panes J.: SS 6.2
Panvini N.: **SS 2.4**
Paradis V.: SS 1.8, SS 10.4, SS 11.9, SS 13.9, SS 13.10
Park E.J.: SS 8.2
Park H.J.: SS 12.2
Park J.: SS 5.8
Park S.: **SS 5.8**
Park S.J.: **SS 3.2**, **SS 7.4**
Park Y.S.: SS 12.2
Patra A.: SS 8.4
Paulatto L.: SS 13.2, SS 13.10
Pavlovics S.: SS 11.10
Pecorelli A.: **SS 10.2**, SS 12.4
Pelini V.: SS 9.2
Pellegrino S.: SS 11.9
Perelli T.: SS 4.5
Perrone A.: SS 9.9
Perrot A.: **SS 13.9**
Petersen F.: SS 6.4
Petracchini M.: SS 8.8, SS 11.7
Petresc B.: SS 14.10
Petretta M.: SS 14.6
Petridis I.: **SS 7.7**, SS 7.8
Pilipovic Grubor J.: **SS 6.3**
Pilozzi E.: SS 2.8
Plessier A.: SS 1.6, SS 11.9, SS 13.10
Plumb A.: SS 2.2
Polidori T.: SS 5.9
Ponce M.D.: SS 11.1
Pop A.: SS 14.10
Pop T.L.: SS 11.1
Porrello G.: SS 4.1
Porta M.: SS 12.4
Pötter-Lang S.: SS 11.8
Prieditis P.: SS 11.10
Prior J.: SS 4.10
Prokop M.: SS 12.9
Ps S.: **SS 12.5**
Pucciarelli F.: **SS 5.9**
Puckett M.: SS 3.6

### Q

Queiroz M.A.: SS 2.7

### R

Radzina M.: **SS 11.10**
Rafaelsen S.: **SS 2.6**
Ragusi M.: SS 12.4
Rautou P.-E.: SS 1.6, SS 10.4, SS 11.3, SS 11.5, SS 11.9, SS 13.10
Raynaud L.: SS 9.4, SS 13.2
Regge D.: SS 4.1, SS 4.2, SS 8.7
Reiberger T.: SS 11.8
Remen T.: SS 8.5
Renehan A.: SS 4.6
Rengo M.: SS 2.4
Reza L.: SS 6.6
Ricke J.: SS 7.3, SS 7.6
Rimola J.: **SS 6.2**
Rizzo G.: SS 3.1, SS 7.10
Rocha M.S.: SS 2.7
Rock N.: SS 11.1
Romeo V.: SS 14.6
Ronconi E.: SS 13.8
Rondenet C.: SS 9.7
Ronot M.: **SS 1.6**, SS 9.4, SS 10.4, **SS 11.3**, SS 11.5, **SS 11.9**, SS 13.2, SS 13.7, SS 13.9, **SS 13.10**
Rossi M.: SS 13.8
Ruggeri V.: SS 13.4, SS 14.3

### S

Sandhu M.S.: SS 11.4
Sartoris R.: SS 1.6, SS 10.4, SS 11.3, SS 11.5
Sayin B.: SS 1.5
Schaeffer N.: SS 4.10
Scheenen T.: SS 12.9
Schmid-Tannwald C.: SS 7.3, SS 7.6
Schurink N.W.: SS 4.4, SS 5.4, **SS 5.6**, SS 8.1
Semmler G.: SS 11.8
Seror M.: **SS 10.4**
Shah V.: SS 2.3, SS 2.5, SS 14.1
Shah Z.: SS 12.8
Shahbazi Feshtali S.: SS 3.4
Shen J.: SS 9.5
Shetty D.: SS 9.8
Shur J.: **SS 5.3**
Sibert A.: SS 9.4
Simbrunner B.: SS 11.8
Simoni N.: SS 3.7
Simpson A.L.: SS 5.10
Singh B.: SS 2.5
Singh J.: SS 11.4
Siopis E.: **SS 9.9**
Sirlin C.: SS 10.2
Sironi S.: SS 12.4
Smith I.M.: SS 9.8
Smout A.: SS 6.8
Sofia C.: SS 4.7
Solouki A.: SS 14.8
Solskaya Y.: SS 11.10
Son S.Y.: SS 2.9
Song B.: SS 1.9, SS 5.7, SS 10.1, SS 10.5, SS 10.9, SS 12.1, SS 13.5, SS 14.2
Soubrane O.: SS 10.4, SS 11.3, SS 13.9
Souhami A.: SS 11.3
Staal F.: **SS 4.9**, SS 5.1
Staka A.: SS 11.10
Stathis G.: SS 6.10
Steegers E.: SS 13.1
Stephenne X.: SS 11.1
Stephenson J.A.: SS 2.3, SS 2.5, SS 14.1
Stojanovic S.: SS 6.3
Stoker J.: SS 6.1, SS 6.8
Strugnell M.J.: SS 9.8
Suh J.: SS 4.3
Suh Y.S.: SS 2.9
Sun S.: **SS 5.10**
Syczewska M.: SS 1.7

### T

Tabone E.: SS 4.1, SS 4.2
Taghavi M.: SS 4.9, **SS 5.1**
Takahashi N.: SS 12.8
Talei Franzesi C.: SS 12.4
Taourel P.: SS 9.7
Tedesco G.: SS 3.8
Terkivatan T.: SS 13.1
Tesselaar M.: SS 4.9
Thomeer M.G.: **SS 13.1**
Thurston M.: SS 9.8
Tielbeek J.: SS 6.8
Timpani A.: SS 4.2
Tinguely P.: SS 7.1
Tipaldi M.A.: **SS 13.8**
Tirane M.: SS 11.10
Tirkes T.: **SS 12.8**
Tozer P.: SS 6.6
Trauner M.: SS 11.8
Tse J.R.: **SS 9.5**
Tuzzolino F.: **SS 7.7**
Tyulyaeva E.Y.: SS 3.3

### U

Ugarte-Cano C.: SS 2.2
Urraro F.: SS 3.1

### V

Vacher Y.J.L.: SS 4.4, SS 4.7
Vadera S.: **SS 14.1**
Valainathan S.: SS 11.9
Valletta R.: **SS 14.5**
Vallone G.: SS 14.6
van Aalten S.: SS 13.1
Van Beers B.: SS 1.8, SS 11.5
Van der Heide U.A.: SS 5.1
van der Sande M.: SS 8.10
van der Zee D.: SS 8.10
van Griethuysen J.: **SS 5.4**, SS 5.6, SS 8.1
van Kranen S.: SS 5.6
Van Lunenburg J.T.J.: **SS 5.2**
van Praag E.M.: SS 6.1
Van Rijn K.: **SS 6.1**, **SS 6.8**
van Treijen M.: SS 4.9
van Triest B.: SS 4.4, SS 4.7
van Wassenaer E.: SS 6.5
Van Wettere M.: SS 13.10
Vanrusselt J.: SS 14.8
Varma S.: SS 11.1
Vaughan R.: SS 6.4
Venara A.: SS 9.3
Venkatesh S.K.: **SS 1.4**
Vercelli L.: SS 8.8, **SS 11.7**
Verma R.: SS 2.3, SS 2.5
Vernuccio F.: SS 10.6, **SS 10.10**, **SS 13.3**
Vicini S.: SS 2.4
Vilgrain V.: SS 1.6, SS 1.8, SS 9.4, SS 10.4, SS 11.3, SS 11.5, SS 11.9, SS 13.2, SS 13.3, SS 13.7, SS 13.9, SS 13.10
Vinokurova L.V.: SS 3.3
Vliegen R.F.A.: SS 5.4
Vogel W.V.: SS 4.4
Vogrig C.: **SS 8.5**
Volpes R.: **SS 7.7**, SS 7.8
Volterrani L.: SS 9.2
Vullierme M.-P.: SS 12.7, SS 13.2

### W

Wasser M.: SS 3.4
Wawrzynowicz-
Weber S.: SS 7.1
Wei H.: **SS 1.9**, **SS 10.1**
Wei Y.: SS 13.5, **SS 14.2**
Westwood T.: SS 4.6
Wildhaber B.: SS 11.1
Wilkens R.: SS 6.4, SS 6.7
Wit H.: SS 9.8
Wu B.: SS 8.3, SS 8.6

### Y

Yadav D.: SS 12.8
Yang L.: **SS 8.3**, **SS 8.6**
Ye Z.: **SS 10.5**
Yoh T.: SS 13.9
Yoo J.: SS 1.3, SS 3.10, SS 5.5, SS 13.6
Yoon J.H.: SS 2.9
Yu H.: **SS 12.1**
Yu M.H.: SS 12.2

### Z

Zamboni G.A.: SS 12.6, SS 12.7, SS 14.5
Zámecnik P.: SS 12.9
Zappa M.: SS 11.5
Zerunian M.: SS 5.9
Zhang T.: **SS 13.5**
Zhang Z.: **SS 5.7**, SS 10.1, **SS 10.9**
Zheng T.: SS 10.1
Zins M.: SS 9.7
Zipoli G.: SS 9.2
Zulkernine F.: SS 5.10
